# De Novo Design of Imidazopyridine-Tethered Pyrazolines That Target Phosphorylation of STAT3 in Human Breast Cancer Cells

**DOI:** 10.3390/bioengineering10020159

**Published:** 2023-01-24

**Authors:** Akshay Ravish, Rashmi Shivakumar, Zhang Xi, Min Hee Yang, Ji-Rui Yang, Ananda Swamynayaka, Omantheswara Nagaraja, Mahendra Madegowda, Arunachalam Chinnathambi, Sulaiman Ali Alharbi, Vijay Pandey, Gautam Sethi, Kwang Seok Ahn, Peter E. Lobie, Basappa Basappa

**Affiliations:** 1 Laboratory of Chemical Biology, Department of Studies in Organic Chemistry, University of Mysore, Manasagangotri, Mysore 570006, India; 2 Shenzhen Bay Laboratory, Shenzhen 518055, China; 3 Department of Science in Korean Medicine, Kyung Hee University, Seoul 02447, Republic of Korea; 4 Tsinghua Berkeley Shenzhen Institute, Tsinghua Shenzhen International Graduate School, Tsinghua University, Shenzhen 518055, China; 5 Department of Studies in Physics, University of Mysore, Manasagangotri, Mysore 570006, India; 6 Department of Botany and Microbiology, College of Science, King Saud University, P.O. Box 2455, Riyadh 11451, Saudi Arabia; 7 Institute of Biopharmaceutical and Health Engineering, Tsinghua Shenzhen International Graduate School, Tsinghua University, Shenzhen 518055, China; 8 Department of Pharmacology, Yong Loo Lin School of Medicine, National University of Singapore, Singapore 117600, Singapore

**Keywords:** human breast cancer, STAT3, imidazopyridine, pyrazolines, DFT, de novo design

## Abstract

In breast cancer (BC), STAT3 is hyperactivated. This study explored the design of imidazopyridine-tethered pyrazolines as a de novo drug strategy for inhibiting STAT3 phosphorylation in human BC cells. This involved the synthesis and characterization of two series of compounds namely, 1-(3-(2,6-dimethylimidazo [1,2-a]pyridin-3-yl)-5-(3-nitrophenyl)-4,5-dihydro-1H-pyrazol-1-yl)-2-(4-(substituted)piperazin-1-yl)ethanone and N-substituted-3-(2,6-dimethylimidazo[1,2-a]pyridin-3-yl)-5-(3-nitrophenyl)-4,5-dihydro-1H-pyrazoline-1-carbothioamides. Compound **3f** with 2,3-dichlorophenyl substitution was recognized among the tested series as a lead structure that inhibited the viability of MCF-7 cells with an IC_50_ value of 9.2 μM. A dose- and time-dependent inhibition of STAT3 phosphorylation at Tyr705 and Ser727 was observed in MCF-7 and T47D cells when compound **3f** was added in vitro. Calculations using density functional theory showed that the title compounds HOMOs and LUMOs are situated on imidazopyridine-pyrazoline and nitrophenyl rings, respectively. Hence, compound **3f** effectively inhibited STAT3 phosphorylation in MCF-7 and T47D cells, indicating that these structures may be an alternative synthon to target STAT3 signaling in BC.

## 1. Introduction

Breast cancer (BC) is the most common cancer in women [1,2,3,4]. As shown in numerous studies, various drugs such as tamoxifen, vinflunine, anastrazole, 5-fluorouracil, Doxorubicin, paclitaxel/docetaxel, ribociclib, olaparib, and other drugs have been shown to possess therapeutic potential for women with BC depending on the stage and the specific molecular subtype [5,6,7,8,9,10,11,12]. Genes that encode transcription factors are directly involved in breast cancer progression, proliferation, apoptosis, metastasis, and chemotherapy resistance [13,14]. STAT3 is one such transcription factor that harbors six functional domains, including the terminal-NH_2_ domain, the coiled-coil domain, the DNA-binding domain, the SRC homology 2 domain, and the transactivation domain [15,16]. STAT3 is activated by both tyrosine and serine phosphorylation and translocates into the nucleus to regulate transcription [17,18,19].

**Figure 1:**
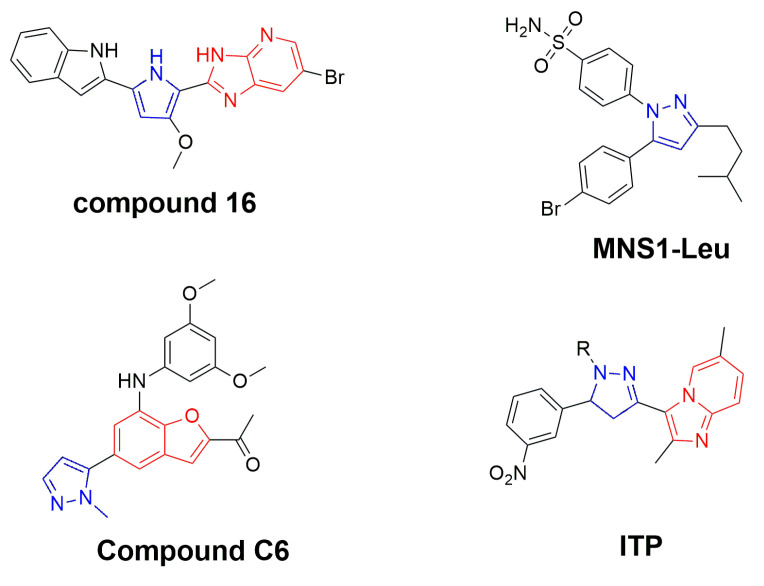
Conventional de novo design of novel ITPs as STAT3 inhibitors in cancer cells.

De novo drug design retains the potential for the discovery of new and potent lead molecules for oncology [20]. Pharmacology has heavily utilized imidazopyridine scaffolds in drug development [21]. Ten approved drugs contain imidazopyridine, and another 12 are in active clinical development [22,23]. A synthetic imidazopyridine compound 16, was synthesized, tested, and determined to reduce the level of phospho-STAT3 and downstream signaling cascades in hepatocellular carcinoma cells, which was attributed to an increase in SHP-1A [24]. Furthermore, the design and synthesis of an imidazopyridine scaffold-bearing compound (P3971) has been identified as a potent STAT3 inhibitor with an IC50 value of 350 nM and demonstrated significant antiproliferative activity against a variety of cancerous cell lines including HCT116 and H460 [25]. Additionally, pyrazoles may provide better pharmacological effects, and have also generated a number of drugs such as pyrazofurin, celecoxib, ramifenazone, lonazolac, and rimonabant [26,27,28,29,30]. We reported the synthesis of pyridine-fused pyrazoles as a STAT3 inhibitor and an inhibitor of cancer cell growth [31]. Additionally, the pyrazole-based compound MNS1-Leu inhibited IL-6-induced STAT3 phosphorylation in patient-derived HGG cells without adversely affecting Akt, STAT1, JAK2, or ERK1/2 phosphorylation [32]. Compound C6 was also discovered to be a STAT3-specific inhibitor that had the strongest anti-proliferation activities against breast cancer cells with an IC_50_ value of 160 nM [33]. In light of this, we developed a conventional de novo design and identified the essential output structures comprising imidazopyridine, pyrazole, pyrrole, and proposed a series of imidazopyridine-tethered pyrazolines (ITP) that could target STAT3 in breast cancer cells based on synthetic accessibility and chemical stability (Figure 1).

## 2. Results and Discussion

**Scheme 1:**
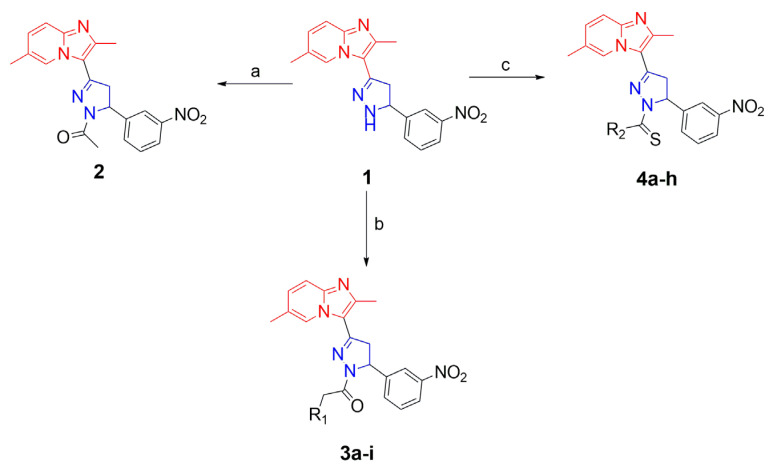
Synthesis of 2-pyrazolines: reagents and conditions: (**a**) AcOH, EtOH, reflux; (**b**) (i) chloroactetyl chloride, toluene, 0–5 °C, (ii) substituted piperazines, acetone, NEt_3_, reflux; (**c**) substituted isothiocyanates, toluene, NEt_3_, reflux.

Using the literature method, we initially synthesized an intermediate (1-[2-methyl-imidazo[1,2-α] pyridine-3-yl]ethanone) by reacting 2-aminopridine with 3-bromopentane-2,4-dione [34]. Further, the chalcone [(E)-1-(2,6-dimethylimidazo[1,2-α]pyridin-3-yl)-3-(3-nitrophenyl)prop-2-en-1-one] was synthesized by the condensation reaction between the intermediateate and 3-nitro-benzaldehyde in presence of alcoholic NaOH, using the literature protocol [35]. The 2-pyrazoline compound **1** [(2,6-dimethyl-3-(5-(3-nitrophenyl)-4,5-dihydro-1H-pyrazol-3-yl)imidazo[1,2-a]pyridine)] was synthesized by refluxing the chalcone and hydrazine hydrate in ethanol solvent. The synthesis of compound **2** [(1-(3-(2,6-dimethylimidazo[1,2-a]pyridin-3-yl)-5-(3-nitrophenyl)-4,5-dihydro-1H-pyrazol-1-yl)] was synthesized by reacting compound **1** with ethanol via acidic dehydration reaction. Furthermore, the synthesis of 2-pyrazoline compounds (**3a–i**) [1-(3-(2,6-dimethylimidazo[1,2-a]pyridin-3-yl)-5-(3-nitrophenyl)-4,5-dihydro-1H-pyrazol-1-yl)-2-(4-substitutted-piperazin-1-yl)ethanones] was prepared by reacting the compound **1** with chloroactetyl chloride, and substituted-piperazines under basic condition. The compounds **4**(**a–h**) [3-(2,6-dimethylimidazo[1,2-a]pyridin-3-yl)-N-substituted-5-(3-nitrophenyl)-4,5-dihydro-1H-pyrazole-1-carbothioamide] were synthesized by reacting compound **1** with substituted-isothiocyanates under basic condition (Scheme 1, Table 1). All synthesized compounds were characterized by ^1^H NMR, ^13^C NMR, and LCMS techniques. The NMR spectral peaks were assigned to all the compounds, which were consistent with the theoretical calculations.

**Figure 2:**
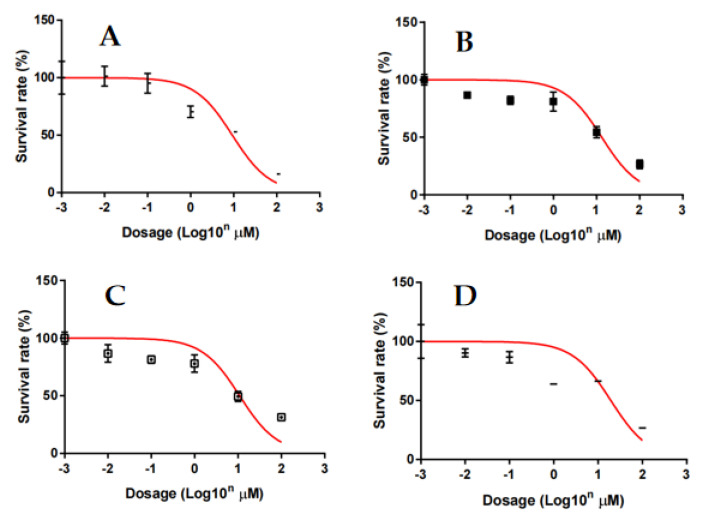
Inhibitory effect of compounds **3f** (**A**), **3e** (**B**), **4g** (**C**), and **3g** (**D**) on the viability of MCF-7 cells. MCF-7 cells were treated with various concentrations of 2-pyrazolines for 72 h and the viability of cells was analyzed by Alamar Blue assays. The results are presented as mean ± S.E.M. of triplicate determinations.

The goal herein was to assess the ability of imidazopyridine-tethered pyrazolines to inhibit cell viability in mammary carcinoma cells (using the Alamar Blue assay), since pyridine-fused pyrazoles were previously shown to inhibit cell viability in various breast cancer cells [36,37]. Results of the study indicated that compounds such as (**3f**), (**3e**), (**4g**), and **3g** inhibited MCF-7 cell viability with IC_50_ values of 9.27, 13.24, and 10.90 μM, respectively (Figure 2). These compounds, which contained substitutions of 2,3-dichlorophenyl, 4-chlorophenyl, 2,5-disubstituted trifluorophenyl, 2-fluoro,4-chloro-phenyl groups as the side chain, exhibited relative inhibition of MCF-7 cell viability. The viability of T47D, BT-474, and SK-BR-3 cells was inhibited in a dose-dependent manner by the lead compound **3f** indicating that the compound was efficacious to decrease the viability of human breast cancer cells (Table 2; Supplementary Figures). The low chemical stability of thio-urea-based compounds may account for the inactivity of most thiourea-conjugated pyrazolines (excluding 4g) against TNBC cells. Further, compounds **3f**, **3h**, and **4b** showed significant effect against TNBC cells, but were not toxic to the normal MCF-10A cells, indicating their selectivity towards cancer cell cytotoxicity.

Further, the in silico mode-of-action analysis was performed for lead compound **3f** using CHEMBL’s latest version as described by kalakoti et al., [38]. For this purpose, the smile format of compound **3f** was added into the similarity searching engine of CHEMBL, which yielded 14,856 bio-activity profiles and which gave the choice of organism, cell type, and also the predicted human targets in the similarity ranking order [39]. The analysis of the results sheet identified STAT3 as a target for compound **3f** with a ranking of 4783 indicating the predicted score would be reasonable to test in in vitro functional studies.

**Figure 3:**
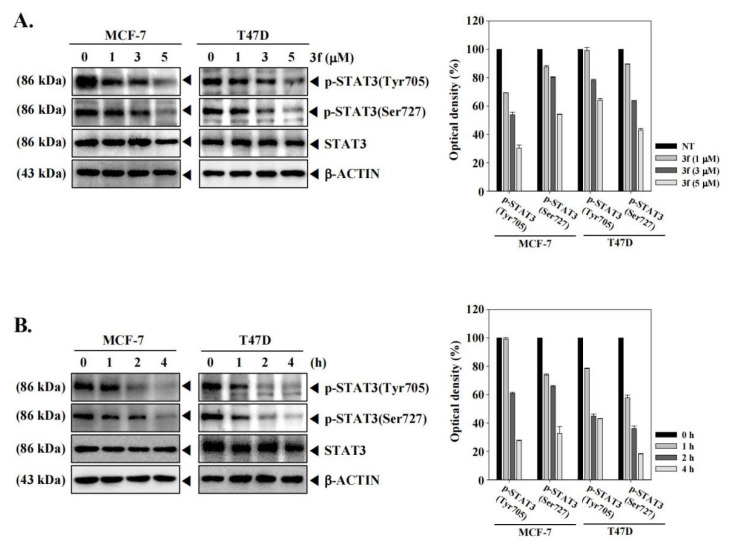
The lead compound **3f** decreases the phosphorylation of STAT3 in MCF-7 and T47D cells in a concentration (**A**) and time-dependent (**B**) manner. MCF-7 and T47D cells were treated with the indicated concentration of **3f** for 4 h. MCF-7 and T47D cells were treated with 5 µM of compound for the indicated time intervals. Thereafter, equal amounts of lysates were analyzed by Western blot analysis using antibodies against p-STAT3(Tyr705), p-STAT3(Ser727), or STAT3. The expression of each protein was quantified. β-ACTIN was used as input control.

Therefore, in vitro experiments were performed to evaluate the effect of compound **3f** on the phosphorylation of STAT3 in MCF-7 and T47D cells, utilizing our laboratory protocol [40]. For this purpose, Western blot analysis was performed after the treatment of MCF-7 and T47D cells with compound **3f** (0, 1, 3, 5 μM) and using p-STAT3 (Tyr705), p-STAT3 (Ser727), and STAT3 antibodies for blotting. Analysis of the results indicated that compound **3f** reduced the phosphorylation level of STAT3 at Tyr705 and Ser727 in a dose-dependent manner without affecting the total STAT3 protein expression (Figure 3A). Furthermore, compound **3f** also reduced the phosphorylation of STAT3 at Tyr705 and Ser727 in a time-dependent manner in both MCF-7 and T47D cells (Figure 3B). These results clearly suggest that compound **3f** decreased the constitutive phosphorylation of STAT3 in ER+ breast cancer cell lines.

**Figure 4:**
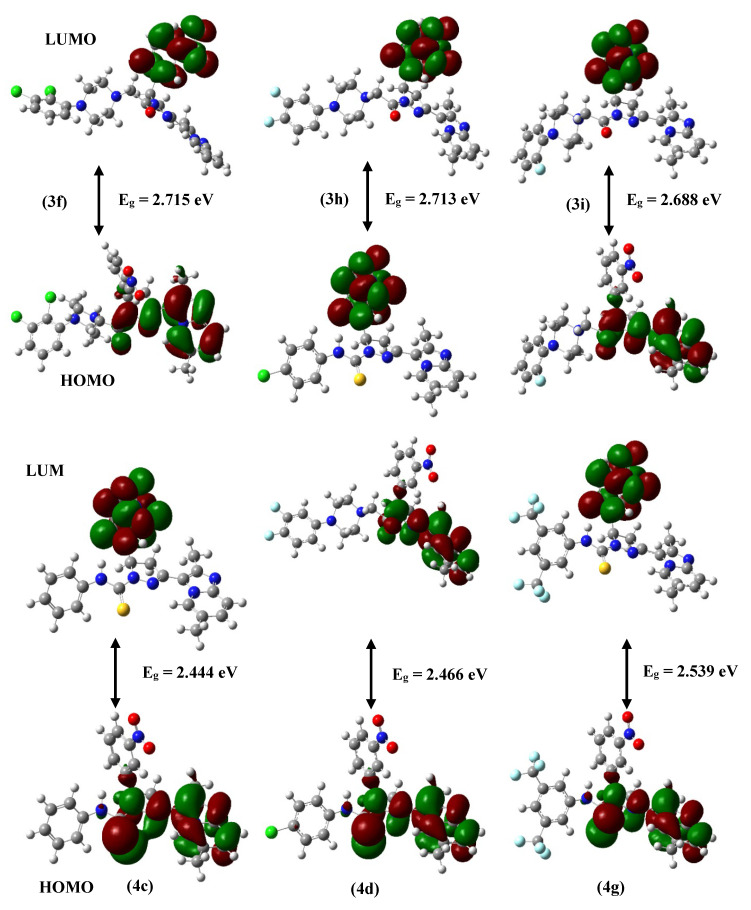
Computed molecular orbital plots for synthesized molecules.

In order to understand the lead structure specificity in the bioactivity of the ITP compounds, DFT calculations were performed. From the frontier molecular orbital (FMOs) theory, HOMO and LUMO are the most influential factors in bioactivity. HOMO has the priority to provide electrons, while LUMO can accept electrons. Moreover, the difference in energy between these two FMOs can be used to predict the strength and stability of molecular complexes [41]. Figure 4 shows the molecular orbital of compounds, while Table 2 lists the calculated global chemical reactivity descriptor parameters of compounds.

HOMO-LUMO levels indicate the interactions between the compound and the protein target. Usually, the HOMO of the compound interacts with the LUMO of the target for binding, and vice versa. A higher HOMO and lower LUMO energies of the molecule imply greater target stability and binding. The lower HOMO-LUMO gap indicates that the lead has lower kinetic stability or higher chemical reactivity and polarizability. Compounds **4g** and **3f** have the most considerable ionization potential among the compounds, 6.905 eV and 6.764 eV, respectively. All of the synthesized molecules are stable within the permitted limits. Molecules with high polarizability are chemically more soft or reactive, related to chemical hardness. Figure 4 shows contour plots of the HOMO and LUMO for compounds **3f** and **4g**. The green and red contours surrounding the atoms represent the negative and positive lobes of wave functions, respectively. The green and red contours encircling the atoms depict the wave function’s negative and positive lobes. It is clear from the plots that the HOMO is localized on the dimethylimidazole-pyridin, pyrazoline sites, and O and S atoms of all the molecules. In contrast, LUMO is localized on the nitrophenyl ring in all the molecules.

**Figure 5:**
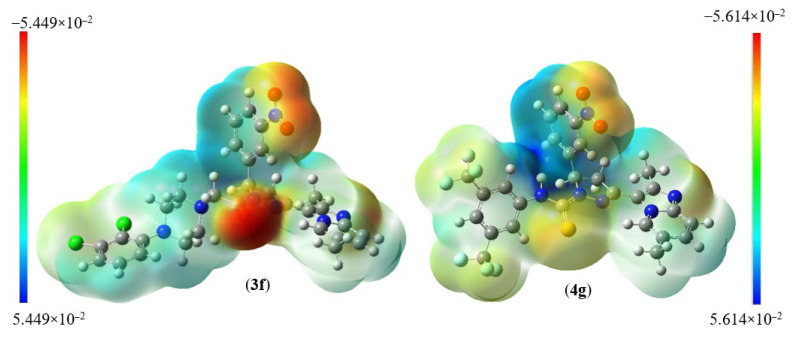
Molecular electrostatic charge distribution plots for **3f** and **4g**.

The distribution of electrostatic potential (EP) over atomic sites is represented by the molecular electrostatic potential (MEP) profile, which can be connected to the partial charge distribution, the electronegativity of atoms in lead molecules, and their interactions. The MEP plots of compounds **3f** and **4g** are shown in Figure 5, where the EP varies from the negative (red) to the positive value (blue) in the sequence given by the color spectrum: red (negative) < orange < yellow < green < blue (positive). The negative EPs are located on the O atomic sites of C=O and N-O2, which indicate that these sites are electron-rich. The positive EPs are seen on H atoms, particularly for H attached to the N, indicating this is an electron-deficient site. Depending on the nature of EPs, these sites would prefer to bind to sites having the opposite potential in the binding pocket or hydrogen bonding interaction (Table 3). For example, the electron-rich C=O should combine with positively charged protons of amino acid residues present in the binding pocket.

The rigid docking method analyzed the synthesized compounds **3f** and **4g** [42]. AutoDock4.2 was used to determine the orientation of inhibitors bound to STAT3 (PDB ID: 1BG1) and the conformation with the highest binding energy value for each molecule. The binding modes of STAT3 inhibitors were analyzed using the PyMOL software to identify new STAT3 inhibitors. The binding site at the SH2 domain of STAT3 was described by Becker et al. [43]. It was used to elucidate the interactions that contributed to the compounds’ binding affinity to STAT3.

**Figure 6:**
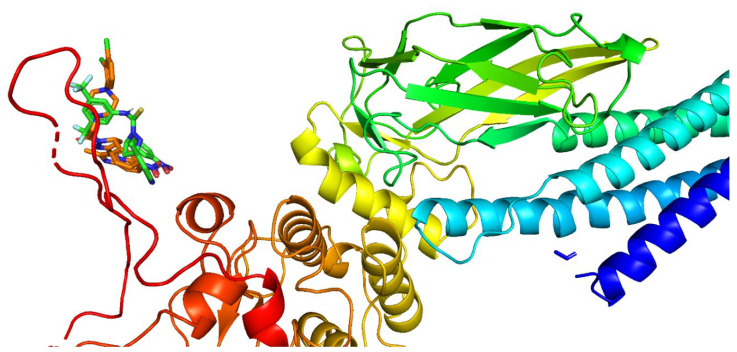
Cartoon representation of the SH2 domain of STAT3 with compounds **3f** and **4g** at the binding pocket.

**Figure 7:**
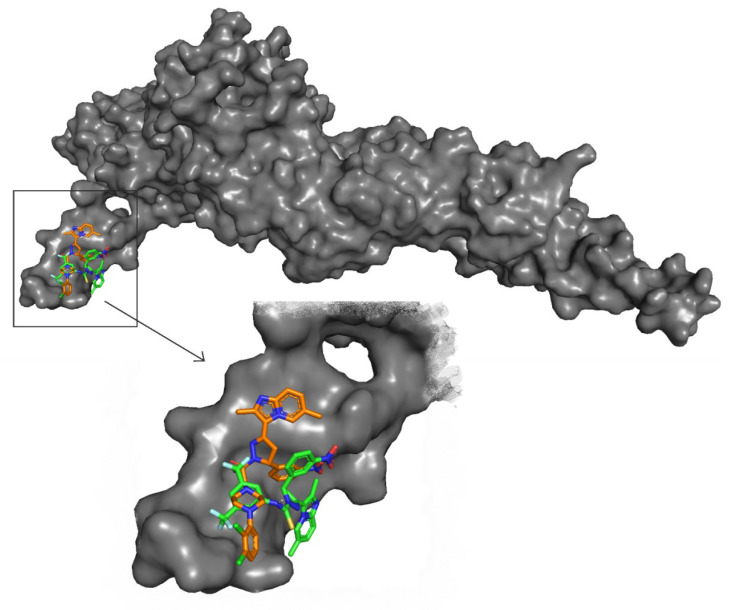
Surface view of STAT3 and the molecules (**3f** and **4g**) and a zoomed view of the molecules in the SH2 domain of STAT3.

**Figure 8:**
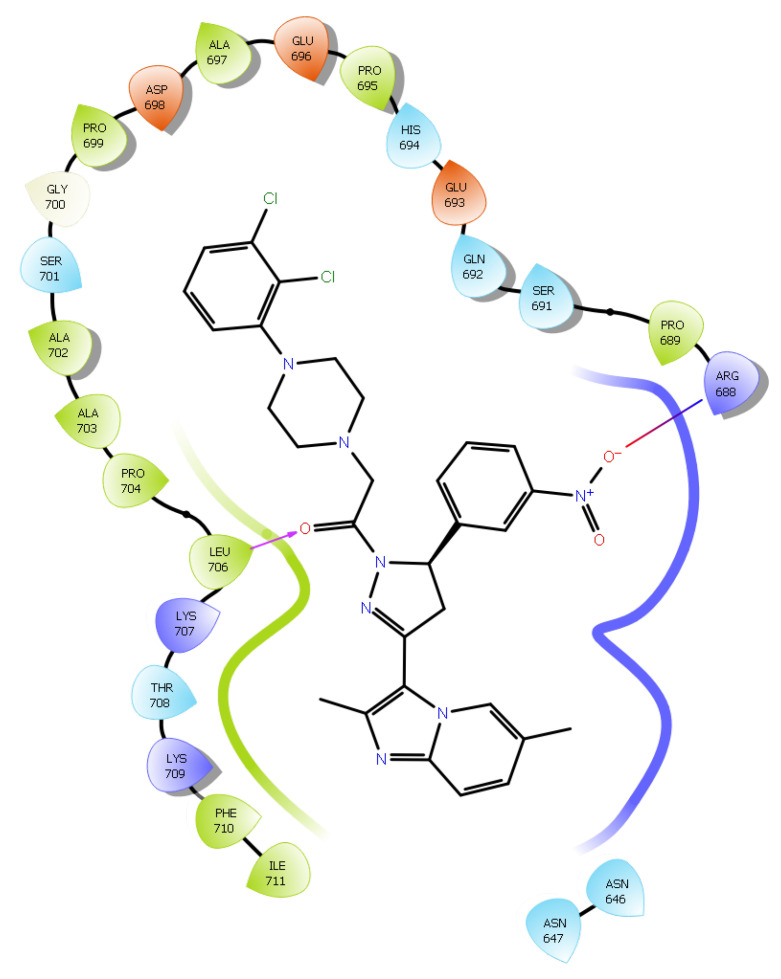
Two-dimensional (2D) interaction diagram of compound **3f** in the SH2 domain of STAT3.

**Figure 9:**
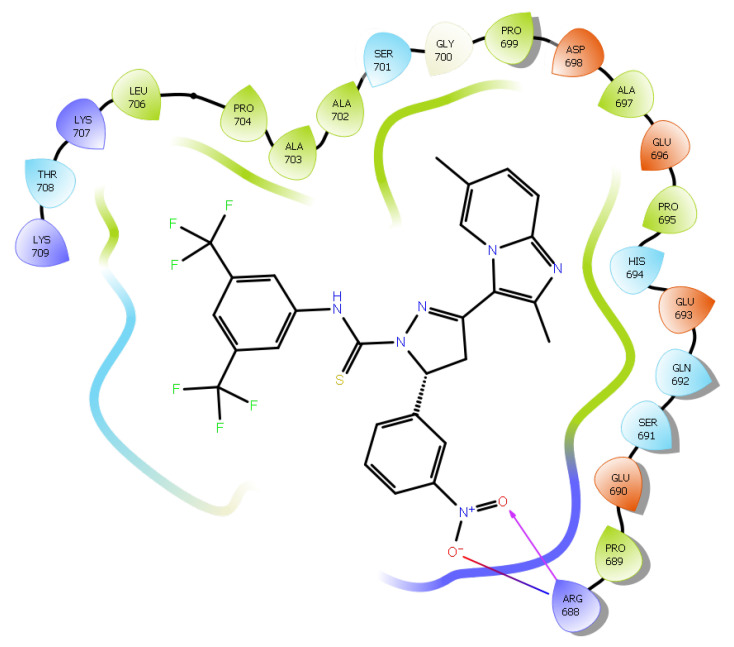
Two-dimensional (2D) interaction diagram of compound **4g** in the SH2 domain of STAT3.

The promising binding modes of **3f** and **4g** at the SH2 domain of the STAT3 protein were analyzed. Figure 6 and Figure 7 show the ligand and receptor complex poses with the highest binding energy. The binding energies of **3f** and **4g** to the SH2 domain of STAT3 were observed to be −9.27 kcal/mol and −6.95 kcal/mol, respectively, indicating that the molecule has a high affinity for the target. The binding patterns of the lead molecules **3f** and **4g** were studied. Both molecules bound to the same site on the receptor molecule (STAT3) and exhibited similar interactions with the vital amino acids of the SH2 domain of STAT3. The docking results showed that the ketone group of **3f** forms a hydrogen bond with Leu706 of the SH2 domain, and the nitro group of the same molecule interacts with ARG688 via a salt bridge. One of the oxygens in the nitro group of **4g** forms a hydrogen bond and the other oxygen participated in the interaction through a salt bridge with the same ARG688 (Figure 8). To summarize, the presence of nitro groups (Figure 8 and Figure 9) in the molecules and structural flexibility facilitates its interaction with the SH2 domain of STAT3.

**Figure 10:**
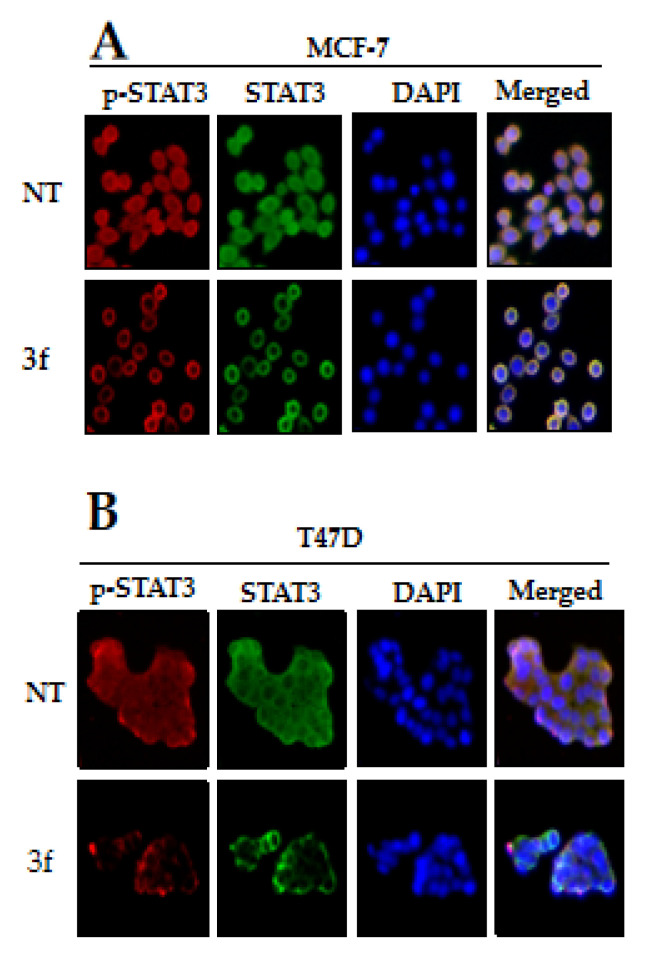
The nuclear localization of STAT3 in MCF-7 (**A**) and T47D (**B**) cells was inhibited by compound **3f**. MCF-7 and T47D cells were treated with the compound **3f** (5 μM), and then analyzed for intracellular distribution by immunocytochemistry. The results shown are representative of three independent experiments. The fluorescence intensity of p-STAT3 and STAT3 was performed. The merged image indicates the overlapping of p-STAT3/STAT3/DAPI images. The results shown are representative of three independent experiments.

Finally, we attempted to understand the effect of compound **3f** on the blocking of pSTAT3 into nuclear functionally since pSTAT3 dimers could enter the nucleus to give transcription. For this purpose, we conducted an immunocytochemistry assay using MCF-7 and T47D cells. We observed that compound **3f** inhibited the nuclear translocation of STAT3 in MCF-7 and T47D cells. We also analyzed the distribution of phospho-STAT3 in the nucleus and cytoplasm using fluorescent-labeled antibodies. Compound **3f** could block the nuclear translocation of pSTAT3 in MCF-7 and T47D cells, as shown in Figure 10A,B, respectively.

## 3. Materials and Methods

All chemicals and solvents for chemistry were purchased from Sigma-Aldrich and TCI chemicals, INDIA. The completion of the reaction was monitored by pre-coated silica gel TLC plates. ^1^H and ^13^C NMR were recorded on an Agilent NMR spectrophotometer (400 MHz); TMS was used as an internal standard and CDCl_3_ was used as a solvent, chemical shifts are expressed as ppm.

### 3.1. General Procedure for the Synthesis of Imidazole-Pyridine Substituted Pyrazoline Derivatives

1-(2,6-dimethylimidazo[1,2-a]pyridin-3-yl)ethenone reacting with 3-nitrobenzaldehyde in 30% KOH gives chalcone which on treating with hydrazine hydrate gives 2-pyrazolines. The synthesis of the compound was reported in earlier reports and complies with the reported molecule [34,35].

### 3.2. Synthesis of 2-Pyrazoline Derivatives (***3a–i***) from 2,6-Dimethyl-3-(5-(3-nitrophenyl)-4,5-dihydro-1H-pyrazol-3-yl)imidazo[1,2-a]pyridine

One mmol of (**1**) was dissolved in toluene and kept in an ice bath. After 15 min triethylamine (1.5 mmol) was added in portions followed chloroacetyl chloride (1.5 mmol) and the reaction was monitored by TLC. After the completion of the reaction, toluene was distilled off under reduced pressure and the residue was extracted with chloroform. The crude solid was used directly in the next reaction without further purification. The crude solid (1 mmol) and substituted piperazines (1 mmol) were dissolved in acetone and refluxed overnight with triethylamine (1 mmol). TLC was monitored and acetone was distilled off, the crude obtained was extracted with chloroform and recrystallized using DCM-Hexane to afford substituted pyrazoline derivatives(**3a–i**).

### 3.3. Synthesis of 2-Pyrazoline Derivatives (***4a–h***) from 2,6-Dimethyl-3-(5-(3-nitrophenyl)-4,5-dihydro-1H-pyrazol-3-yl)imidazo[1,2-a]pyridine

Pyrazoline derivative (**1**) (1 mmol) and substituted isothiocyanates (1.2 mmol) were dissolved in toluene and refluxed overnight with triethylamine (1 mmol). The reaction was monitored by TLC and reaction mass was filtered, washed with hexane to remove unreacted isothiocyanate, and dried in hot air oven to yield pure substituted thiourea derivatives (**4a–h**).

### 3.4. 1-(3-(2,6-Dimethylimidazo[1,2-a]pyridin-3-yl)-5-(3-nitrophenyl)-4,5-dihydro-1H-pyrazol-1-yl)ethanone (***2***)

Yellow solid; MP: 166–168 °C; yield: 92%;^1^H NMR (400 MHz, CDCl_3_): δ 9.28 (s, 1H), 8.14–8.11 (m, 2H), 7.62 (d, J = 7.6 Hz, 1H), 7.56–7.52 (m, 2H), 7.30–7.23 (m, 1H), 5.65 (dd, J = 11.7, 4.7 Hz, 1H, CH), 4.06 (dd, J = 11.6, 5.6 Hz, 1H, CH_2_), 3.35 (dd, J = 17.2, 4.8 Hz, 1H, CH_2_), 2.59 (s, 3H, CH_3_), 2.50 (s, 3H, CH_3_), 2.44 (s, 3H, CH_3_); ^13^C NMR (100 MHz, CDCl_3_): δ 173.40, 153.78, 153.32, 151.49, 150.77, 148.72, 137.10, 135.07, 134.93, 131.57, 128.63, 127.91, 125.82, 121.01, 118.30, 62.92(CH), 49.65(CH_2_), 27.07(CH_3_), 23.77(CH_3_), 21.48(CH_3_); MS = 377.1488, *m/z* = 378.1891 [M + 1]^+^

### 3.5. 1-(2-(3-(2,6-Dimethylimidazo[1,2-a]pyridin-3-yl)-5-(3-nitrophenyl)-4,5-dihydro-1H-pyrazol-1-yl)-2-oxoethyl)piperidin-4-one (***3a***)

Yellow solid; ^1^H NMR (400 MHz, CDCl_3_): δ 9.21 (s, 1H), 8.21–8.06 (m, 2H), 7.64 (d, J = 7.4 Hz, 1H), 7.54–7.53 (m, 2H), 7.27 (d, J = 9.8 Hz, 1H), 5.66 (dd, J = 11.5, 4.6 Hz, 1H, CH), 4.05 (dd, J = 17.1, 11.9 Hz, 1H, CH), 3.96 (d, J = 16.2 Hz, 1H, COCH_2_), 3.83 (d, J = 16.2 Hz, 1H, COCH_2_), 3.36 (dd, J = 17.2, 4.6 Hz, 1H, CH), 3.09–2.86 (m, 4H, (CH_2_)_2_), 2.59 (s, 3H, CH_3_), 2.51 (t, J = 5.6 Hz, 4H, (CH_2_)_2_), 2.44 (s, 3H, CH_3_); ^13^C NMR (100 MHz, CDCl_3_): δ 213.42, 172.00, 153.82, 150.90, 148.42, 137.20, 135.18, 135.07, 131.44, 128.70, 128.05, 125.73, 121.17, 118.15, 82.39, 82.07, 81.76, 63.71, 63.19, 58.41, 49.26, 46.30((CH_2_)_2_), 23.84(CH_3_), 21.58(CH_3_); MS = 474.2016, *m/z* = 475.2095 [M + 1]^+^

### 3.6. Tert-butyl-4-(2-(3-(2,6-dimethylimidazo[1,2-a]pyridin-3-yl)-5-(3-nitrophenyl)-4,5-dihydro-1H-pyrazol-1-yl)-2-oxoethyl)piperazine-1-carboxylate (***3b***)

Yellow solid; ^1^H NMR (400 MHz, cdcl3): δ 9.21 (s, 1H), 8.13 (d, J = 8.2 Hz, 1H), 8.09 (s, 1H), 7.62 (d, J = 7.8 Hz, 1H), 7.59–7.47 (m, 2H), 7.29–7.25 (m, 1H), 5.64 (dd, J = 11.8, 4.9 Hz, 1H, CH), 4.04 (dd, J = 17.3, 11.9 Hz, 1H, CH_2_), 3.82 (d, J = 16.0 Hz, 1H, COCH_2_), 3.69 (d, J = 16.0 Hz, 1H, COCH_2_), 3.48 (s, 4H, (CH_2_)_2_), 3.35 (dd, J = 17.3, 5.0 Hz, 1H, CH_2_), 2.65–2.59 (m, 4H, (CH_2_)_2_), 2.59 (s, 3H, CH_3_), 2.44 (s, 3H, CH_3_), 1.44 (s, 9H, (CH_3_)_3_); ^13^C NMR (100 MHz, CDCl_3_): δ 171.94, 159.69, 153.80, 153.68, 151.91, 150.87, 148.48, 137.18, 135.12, 135.03, 131.48, 128.67, 127.99, 125.79, 121.12, 118.20, 84.70(C(CH_3_)), 64.68(CH_2_), 63.15(CH), 58.33(CH_2_)_2_, 50.48(CH_2_)_2_, 49.23(CH_2_), 33.45(CH_3_)_3_, 23.80(CH_3_), 21.53(CH_3_).

### 3.7. 1-(3-(2,6-Dimethylimidazo[1,2-a]pyridin-3-yl)-5-(3-nitrophenyl)-4,5-dihydro-1H-pyrazol-1-yl)-2-(piperazin-1-yl)ethanone (***3c***)

Yellow; ^1^H NMR (400 MHz, CDCl_3_): δ 9.20 (s, 1H), 8.14 (d, *J* = 7.5 Hz, 1H), 8.10 (s, 1H), 7.70–7.48 (m, 3H), 7.28 (s, 1H), 5.65 (d, *J* = 7.2 Hz, 1H, CH), 4.04 (dd, *J* = 12.0, 16.4 Hz, 1H, CH_2_), 3.78 (dd, *J* = 16.0, 16.0 Hz, 2H, COCH_3_), 3.36 (d, *J* = 16.8 Hz, 1H, CH_2_), 3.08 (s, 2H, (CH_2_)_2_), 2.81 (d, *J* = 18.0 Hz, 2H, (CH_2_)_2_), 2.60 (s, 3H, CH_3_), 2.47 (s, 3H, CH_3_), 1.26 (s, 1H, NH).

### 3.8. 2-(4-Acetylpiperazin-1-yl)-1-(3-(2,6-dimethylimidazo[1,2-a]pyridin-3-yl)-5-(3-nitrophenyl)-4,5-dihydro-1H-pyrazol-1-yl)ethanone (***3d***)

Brown solid; ^1^H NMR (400 MHz, CDCl_3_): δ 9.20 (s, 1H), 8.20–8.05 (m, 2H), 7.62–7.53 (m, 3H), 7.28–7.26 (m, 1H), 5.64 (d, J = 7.3 Hz, 1H, CH), 4.09–3.99 (m, 1H, CH_2_), 3.85 (d, J = 15.9 Hz, 1H, COCH_2_), 3.67 (t, J = 26.2 Hz, 4H, (CH_2_)_2_), 3.51 (s, 1H, COCH_2_), 3.39–3.32 (m, 1H, CH_2_), 2.66 (d, J = 14.7 Hz, 4H, (CH_2_)_2_), 2.59 (s, 3H, COCH_3_), 2.44 (s, 3H, CH_3_), 2.07 (s, 3H, CH_3_); ^13^C NMR (100 MHz, CDCl_3_): δ 168.90, 166.74, 148.78, 148.66, 146.99, 145.81, 143.35, 132.13, 130.08, 130.03, 126.38, 123.67, 122.96, 120.66, 116.07, 113.10, 59.34(CH), 58.11(CH_2_), 53.31(CH_2_)_2_, 46.18(CH_2_)_2_, 41.35(CH_2_), 21.17(CH_3_), 18.74(CH_3_), 16.42(CH_3_); MS = 503.2281, *m/z* = 504.2384 [M + 1]^+^

### 3.9. 2-(4-(4-Chlorophenyl)piperazin-1-yl)-1-(3-(2,6-dimethylimidazo[1,2-a]pyridin-3-yl)-5-(3-nitrophenyl)-4,5-dihydro-1H-pyrazol-1-yl)ethanone (***3e***)

Brown solid; ^1^H NMR (400 MHz, CDCl_3_): δ 9.25 (s, 1H), 8.14 (d, *J* = 8.0 Hz, 1H), 8.11 (s, 1H), 7.64 (d, *J* = 7.2 Hz, 1H), 7.63–7.46 (m, 2H), 7.26 (s, 1H), 7.17 (d, *J* = 8.0 Hz, 2H), 6.82 (d, *J* = 8.4 Hz, 2H), 5.66 (d, *J* = 7.2 Hz, 1H, CH), 4.05 (dd, *J* = 12.4, 16.0 Hz, 1H, CH_2_), 3.90 (d, *J* = 15.6 Hz, 1H, COCH_2_), 3.74 (d, *J* = 16.0 Hz, 1H, COCH_2_), 3.37 (dd, *J* = 2.8, 16.8 Hz, 1H, CH), 3.23 (s, 4H, (CH_2_)_2_), 2.86 (d, *J* = 16..0 Hz, 4H, (CH_2_)_2_), 2.60 (s, 3H, CH_3_), 2.46 (s, 3H, CH_3_); ^13^C NMR (100 MHz, CDCl_3_): δ 166.80, 154.41, 148.67, 148.40, 146.71, 145.68, 143.38, 132.11, 129.99, 126.42, 124.35, 123.63, 122.86, 122.44, 122.36, 120.72, 118.90, 116.06, 115.95, 115.86, 113.11, 59.60(CH), 58.06(CH_2_), 53.65(CH_2_)_2_, 50.23(CH_2_)_2_, 44.12(CH_2_), 18.69(CH_3_), 16.34(CH_3_); MS = 571.21, *m/z* = 572.26 [M + 1]^+^

### 3.10. 2-(4-(2,3-Dichlorophenyl)piperazin-1-yl)-1-(3-(2,6-dimethylimidazo[1,2-a]pyridin-3-yl)-5-(3-nitrophenyl)-4,5-dihydro-1H-pyrazol-1-yl)ethanone (***3f***)

Yellow solid; ^1^H NMR (400 MHz, cdcl3): δ 9.26 (s, 1H), 8.15–8.13 (m, 2H), 7.65 (d, J = 7.4 Hz, 1H), 7.55–7.51 (m, 2H), 7.26–7.9 (m, 1H), 7.13 (d, J = 6.2 Hz, 2H), 6.95 (d, J = 6.3 Hz, 1H), 5.67 (dd, J = 11.6, 4.6 Hz, 1H, CH), 4.12–4.01 (m, 1H, CH_2_), 3.90 (d, J = 15.9 Hz, 1H, COCH_2_), 3.75 (d, J = 15.8 Hz, 1H, COCH_2_), 3.37 (dd, J = 17.2, 4.6 Hz, 1H, CH_2_), 3.12 (s, 4H, (CH_2_)_2_, 2.88 (d, J = 18.6 Hz, 4H, (CH_2_)_2_), 2.60 (s, 3H, CH_3_), 2.47 (s, 3H, CH_3_); ^13^C NMR (100 MHz, CDCl_3_): δ 167.01, 151.21, 148.77, 148.58, 146.78, 145.82, 143.50, 133.96, 132.17, 130.05, 129.96, 127.51, 127.43, 126.48, 124.60, 123.63, 122.92, 120.76, 118.68, 116.06, 113.19, 59.67(CH_2_), 58.12(CH_2_), 53.74(CH_2_)_2_, 51.18(CH_2_)_2_, 44.19(CH_2_), 18.78(CH_3_), 16.48(CH_3_); MS = 605.17, *m/z* = 606.23 [M + 1]^+^

### 3.11. 2-(4-(4-Chloro-2-fluorophenyl)piperazin-1-yl)-1-(3-(2,6-dimethylimidazo[1,2-a]pyridin-3-yl)-5-(3-nitrophenyl)-4,5-dihydro-1H-pyrazol-1-yl)ethanone (***3g***)

White solid; ^1^H NMR (400 MHz, CDCl_3_): δ 9.25 (s, 1H), 8.18–8.08 (m, 2H), 7.64 (d, J = 7.6 Hz, 1H), 7.59–7.47 (m, 2H), 7.29–7.26 (m, 1H), 7.03–7.00 (m, 2H), 6.84 (t, J = 8.7 Hz, 1H), 5.66 (dd, J = 11.7, 4.8 Hz, 1H, CH_2_), 4.05 (dd, J = 17.2, 11.9 Hz, 1H, CH_2_), 3.88 (d, J = 15.9 Hz, 1H, COCH_3_), 3.73 (d, J = 15.9 Hz, 1H, COCH_3_), 3.36 (dd, J = 17.3, 4.9 Hz, 1H, CH_2_), 3.13 (s, 4H, (CH_2_)_2_), 2.86 (td, J = 10.7, 5.9 Hz, 4H, (CH_2_)_2_), 2.60 (s, 3H, CH_3_), 2.45 (s, 3H, CH_3_); ^13^C NMR (100 MHz, CDCl_3_): δ 166.92, 148.74, 148.61, 146.81, 145.82, 143.46, 138.96, 138.88, 132.19, 130.06, 129.95, 126.47, 124.45, 123.60, 122.93, 120.75, 119.59, 116.87, 116.63, 116.07, 113.17,59.68(CH), 58.12(CH_2_), 53.60(CH_2_)_2_, 50.32(CH_2_)_2_, 44.19(CH_2_), 18.76(CH_3_), 16.50(CH_3_); MS = 589.2004, *m/z* = 590.2119 [M + 1]^+^

### 3.12. 2-(4-(3,4-Difluorophenyl)piperazin-1-yl)-1-(3-(2,6-dimethylimidazo[1,2-a]pyridin-3-yl)-5-(3-nitrophenyl)-4,5-dihydro-1H-pyrazol-1-yl)ethanone (***3h***)

Brown solid; ^1^H NMR (400 MHz, CDCl_3_): δ 9.24 (s, 1H), 8.11 (s, 2H), 7.63–7.53 (m, 3H), 7.25 (s, 1H), 7.30–7.27 (m, 1H), 6.72–6.64 (m, 1H), 6.56 (d, J = 8.2 Hz, 1H), 5.66 (dd, J = 11.8, 4.9 Hz, 1H, CH), 4.05 (dd, J = 17.3, 11.9 Hz, 1H, CH_2_), 3.90 (d, J = 15.9 Hz, 1H, COCH_3_), 3.75 (d, J = 15.9 Hz, 1H, COCH_3_), 3.39–3.33 (m, 1H, CH_2_), 3.19 (s, 4H, (CH_2_)_2_), 2.86 (d, J = 15.5 Hz, 4H, (CH_2_)_2_), 2.60 (s, 3H, CH_3_), 2.46 (s, 3H, CH_3_);^13^C NMR (100 MHz, CDCl_3_): δ 166.60, 148.71, 148.51, 146.83, 145.71, 143.30, 132.10, 130.01, 129.98, 126.38, 123.60, 122.90, 122.89, 120.63, 117.15, 116.98, 116.00, 113.07, 111.44, 105.40, 105.20, 77.21, 76.90, 76.58, 59.34, 58.05, 53.20, 49.28, 44.13, 18.70, 16.37.

### 3.13. 1-(3-(2,6-Dimethylimidazo[1,2-a]pyridin-3-yl)-5-(3-nitrophenyl)-4,5-dihydro-1H-pyrazol-1-yl)-2-(4-(2-fluorophenyl)piperazin-1-yl)ethanone (***3i***)

Brown solid; ^1^H NMR (400 MHz, CDCl_3_): δ 9.25 (s, 1H), 8.14–8.12 (m, 2H), 7.65–7.49 (m, 3H), 7.28–7.24 (m, 1H), 7.04–6.90 (m, 4H), 5.66 (dd, J = 11.5, 4.7 Hz, 1H, CH), 4.04 (dd, J = 17.1, 11.9 Hz, 1H, CH_2_), 3.89 (d, J = 15.9 Hz, 1H, COCH_3_), 3.75 (d, J = 15.9 Hz, 1H, COCH_2_), 3.36 (dd, J = 17.1, 4.4 Hz, 1H, CH_2_), 3.17 (s, 4H, (CH_2_)_2_), 2.88 (d, J = 17.8 Hz, 4H, (CH_2_)_2_), 2.60 (s, 3H, CH_3_), 2.45 (s, 3H, CH_3_); ^13^C NMR (100 MHz, CDCl_3_): δ 166.80, 154.41, 148.67, 148.40, 146.71, 145.68, 143.38, 132.11, 129.99, 126.42, 124.35, 123.63, 122.86, 122.44, 122.36, 120.72, 118.90, 116.06, 115.95, 115.86, 113.11, 59.60(CH), 58.06(CH_2_), 53.65(CH_2_)_2_, 50.23(CH_2_)_2_, 44.12(CH_2_), 18.69(CH_3_), 16.34(CH_3_).

### 3.14. 3-(2,6-Dimethylimidazo[1,2-a]pyridin-3-yl)-5-(3-nitrophenyl)-N-phenyl-4,5-dihydro-1H-pyrazoline-1-carbothioamide (***4a***)

White solid; ^1^H NMR (400 MHz, cdcl3): δ 9.13 (s, 1H), 9.04 (s, 1H), 8.18–8.09 (m, 2H), 7.66 (d, J = 7.7 Hz, 3H), 7.56 (dd, J = 17.5, 8.5 Hz, 2H), 7.37 (t, J = 7.8 Hz, 2H), 7.29 (d, J = 8.8 Hz, 1H), 7.19 (s, 1H), 6.25 (dd, J = 11.6, 3.6 Hz, 1H, CH), 4.15 (dd, J = 17.2, 11.7 Hz, 1H, CH_2_), 3.39 (dd, J = 17.2, 3.7 Hz, 1H, CH_2_), 2.60 (s, 3H, CH_3_), 2.46 (s, 3H, CH_3_); ^13^C NMR (100 MHz, CDCl_3_): δ 173.18, 149.96, 148.64, 147.58, 146.05, 143.87, 138.53, 132.10, 130.31, 129.86, 128.73, 125.92, 125.55, 124.00, 123.73, 122.69, 120.75, 116.29, 112.82, 61.22(CH), 44.57(CH_2_), 18.81(CH_3_), 16.72(CH_3_); MS = 470.1525, *m/z* = 471.1626 [M + 1]^+^

### 3.15. 3-(2,6-Dimethylimidazo[1,2-a]pyridin-3-yl)-5-(3-nitrophenyl)-N-(4-nitrophenyl)-4,5-dihydro-1H-pyrazole-1-carbothioamide (***4b***)

Orange solid; ^1^H NMR (400 MHz, CDCl_3_): δ 10.13 (s, 1H), 9.37 (s, 1H), 9.13 (s, 1H), 8.14 (s, 1H), 8.01-7.94 (m, 3H), 7.54–7.60 (t, *J* = 10.0 Hz, 2H), 7.34 (d, 2H), 6.23 (dd, *J* = 2.0, 2.0 Hz, 1H, CH), 4.19 (dd, *J* = 1.0, 1.0 Hz, 1H, CH_2_), 3.84–3.76 (m, 1H, CH_2_), 2.63 (s, 3H, CH_3_), 2.51 (s, 3H, CH_3_); ^13^C NMR (100 MHz, CDCl_3_): δ 173.18, 149.96, 148.64, 147.58, 146.05, 143.87, 138.53, 132.10, 130.31, 129.86, 128.73, 125.92, 125.55, 124.00, 123.73, 122.69, 120.75, 116.29, 112.82, 61.22(CH), 44.57(CH_2_), 18.81(CH_3_), 16.72(CH_3_); MS = 515.1376, *m/z* = 516.2025 [M + 1]^+^

### 3.16. N-Cyclohexyl-3-(2,6-dimethylimidazo[1,2-a]pyridin-3-yl)-5-(3-nitrophenyl)-4,5-dihydro-1H-pyrazoline-1-carbothioamide (***4c***)

Yellow solid; ^1^H NMR (400 MHz, CDCl_3_): δ 9.10 (s, 1H), 8.08 (s, 2H), 7.64–7.48 (m, 3H), 7.27–7.29 (m, 1H), 7.21 (d, J = 8.1 Hz, 1H), 6.15 (dd, J = 11.7, 3.8 Hz, 1H, CH), 4.07 (dd, J = 17.1, 11.7 Hz, 1H, CH_2_), 3.34 (dd, J = 17.1, 3.9 Hz, 1H, CH_2_), 2.58 (s, 3H, CH_3_), 2.46 (s, 3H, CH_3_), 2.09 (s, 1H, CH), 1.73 (s, 4H, (CH_2_)_2_), 1.54–1.45 (m, 2H, CH_2_), 1.45–1.29 (m, 4H, (CH_2_)_2_); ^13^C NMR (100 MHz, CDCl_3_): δ 168.90, 166.74, 148.78, 148.66, 146.99, 145.81, 143.35, 132.13, 130.09, 126.38, 123.67, 122.96, 120.66, 116.07, 113.10, 59.34(CH), 58.11(CH), 53.31(CH_2_)_2_, 46.18(CH_2_)_2_, 44.18(CH_2_), 41.35(CH_2_), 18.74(CH_3_), 16.42(CH_3_); MS = 476.20, *m/z* = 477.25 [M + 1]^+^

### 3.17. N-(4-Chlorophenyl)-3-(2,6-dimethylimidazo[1,2-a]pyridin-3-yl)-5-(3-nitrophenyl)-4,5-dihydro-1H-pyrazoline-1-carbothioamide (***4d***)

Yellow solid; ^1^H NMR (400 MHz, CDCl_3_): δ 9.11 (s, 1H), 8.97 (s, 1H), 8.15 (d, J = 6.1 Hz, 2H), 7.67 (d, J = 7.6 Hz, 1H), 7.63–7.52 (m, 4H), 7.34 (d, J = 8.7 Hz, 2H), 7.25 (s, 1H), 6.23 (dd, J = 11.5, 3.5 Hz, 1H, CH), 4.16 (dd, J = 17.2, 11.7 Hz, 1H, CH_2_), 3.41 (dd, J = 17.2, 3.6 Hz, 1H, CH_2_), 2.61 (s, 3H, CH_3_), 2.47 (s, 3H, CH_3_); ^13^C NMR (100 MHz, CDCl3): δ 173.18, 149.96, 148.64, 147.58, 146.05, 143.87, 138.53, 132.10, 130.31, 129.86, 128.73, 125.92, 125.55, 124.00, 123.73, 122.69, 120.75, 116.29, 112.82, 61.22(CH), 44.57(CH_2_), 18.81(CH_3_), 16.72(CH_3_); MS = 476.1681, *m/z* = 485.2469 [M + 1]^+^

### 3.18. N-(3-Chlorophenyl)-3-(2,6-dimethylimidazo[1,2-a]pyridin-3-yl)-5-(3-nitrophenyl)-4,5-dihydro-1H-pyrazoline-1-carbothioamide (***4e***)

Yellow solid; ^1^H NMR (400 MHz, CDCl_3_): δ 9.11 (s, 1H), 9.03 (s, 1H), 8.17–8.11 (m, 2H), 7.77 (t, J = 2.0 Hz, 1H), 7.66 (d, J = 7.8 Hz, 1H), 7.60 (d, J = 9.0 Hz, 1H), 7.55 (dd, J = 7.0, 1.7 Hz, 2H), 7.31 (dd, J = 13.8, 4.8 Hz, 2H), 7.18–7.12 (m, 1H), 6.22 (dd, J = 11.6, 3.8 Hz, 1H, CH), 4.16 (dd, J = 17.3, 11.7 Hz, 1H, CH_2_), 3.40 (dd, J = 17.3, 3.9 Hz, 1H, CH_2_), 2.61 (s, 3H, CH_3_), 2.48 (s, 3H, CH_3_).; ^13^C NMR (100 MHz, CDCl_3_): δ 167.01, 151.21, 148.77, 148.58, 146.78, 145.82, 143.50, 133.96, 132.17, 130.05, 129.96, 127.51, 127.43, 126.48, 124.60, 123.63, 122.92, 120.76, 118.68, 116.06, 113.19, 53.74(CH), 51.18(CH_2_), 18.78(CH_3_), 16.48(CH_3_).

### 3.19. 3-(2,6-Dimethylimidazo[1,2-a]pyridin-3-yl)-5-(3-nitrophenyl)-N-(p-tolyl)-4,5-dihydro-1H-pyrazoline-1-carbothioamide (***4f***)

White solid; ^1^H NMR (400 MHz, CDCl_3_): δ 9.13 (s, 1H), 8.93 (s, 1H), 8.17–8.12 (m, 2H), 7.67 (d, J = 7.7 Hz, 1H), 7.59 (d, J = 9.0 Hz, 1H), 7.55 (d, J = 7.9 Hz, 1H), 7.49 (d, J = 8.3 Hz, 2H), 7.30 (dd, J = 9.1, 1.5 Hz, 1H), 7.19 (d, J = 8.2 Hz, 2H), 6.26 (dd, J = 11.6, 3.8 Hz, 1H, CH), 4.15 (dd, J = 17.2, 11.7 Hz, 1H, CH_2_), 3.39 (dd, J = 17.2, 3.8 Hz, 1H, CH_2_), 2.61 (s, 3H, CH_3_), 2.46 (s, 3H, CH_3_), 2.34 (s, 3H, CH_3_); ^13^C NMR (100 MHz, CDCl_3_): δ 173.72, 149.77, 148.71, 147.53, 145.98, 144.00, 135.94, 135.71, 132.21, 130.44, 129.93, 129.41, 125.99, 124.40, 124.11, 122.77, 120.80, 116.30, 112.92, 77.32, 77.00, 76.69, 61.32, 44.62, 21.01, 18.88, 16.74.

### 3.20. N-(3,5-Bis(trifluoromethyl)phenyl)-3-(2,6-dimethylimidazo[1,2-a]pyridin-3-yl)-5-(3-nitrophenyl)-4,5-dihydro-1H-pyrazoline-1-carbothioamide (***4g***)

Yellow solid; ^1^H NMR (400 MHz, CDCl_3_): δ 9.31 (s, 1H), 9.13 (s, 1H), 8.26 (s, 2H), 8.15 (s, 1H), 7.66 (s, 1H), 7.64-7.53 (m, 2H), 7.34 (d, J = 9.0 Hz, 1H), 7.26 (t, J = 3.7 Hz, 1H), 7.18 (s, 1H), 6.21 (dd, J = 11.5, 3.8 Hz, 1H, CH), 4.20 (dd, J = 17.3, 11.6 Hz, 1H, CH_2_), 3.45 (dd, J = 17.3, 3.8 Hz, 1H, CH_2_), 2.63 (s, 3H, CH_3_), 2.49 (s, 3H, CH_3_); ^13^C NMR (100 MHz, CDCl_3_): δ 172.21, 150.70, 148.74, 148.46, 146.34, 143.25, 140.10, 132.24, 131.85, 130.74, 130.06, 129.00, 128.18, 125.98, 125.25, 124.33, 123.03, 122.65, 121.68, 120.79, 118.36, 116.52, 112.64, 61.33(CH), 44.73(CH_2_), 18.73(CH_3_), 16.83(CH_3_); MS = 606.13, *m/z* = 607.19 [M + 1]^+^

### 3.21. N-Butyl-3-(2,6-dimethylimidazo[1,2-a]pyridin-3-yl)-5-(3-nitrophenyl)-4,5-dihydro-1H-pyrazoline-1-carbothioamide (***4h***)

White solid; ^1^H NMR (400 MHz, CDCl_3_): δ 9.09 (s, 1H), 8.16–8.06 (m, 2H), 7.56 (ddd, J = 25.3, 17.7, 7.8 Hz, 3H), 7.28 (dd, J = 9.1, 1.5 Hz, 1H), 7.20 (t, J = 5.1 Hz, 1H), 6.15 (dd, J = 11.7, 3.9 Hz, 1H CH), 4.08 (dd, J = 17.2, 11.8 Hz, 1H, CH_2_), 3.69 (qd, J = 13.2, 6.3 Hz, 2H, CH_2_), 3.34 (dd, J = 17.2, 4.0 Hz, 1H, CH_2_), 2.58 (s, 3H, CH_3_), 2.46 (s, 3H, CH_3_), 1.70 (dd, J = 8.5, 7.1 Hz, 2H, CH_2_), 1.50 (dd, J = 15.1, 7.4 Hz, 2H, CH_2_), 1.00 (t, J = 7.3 Hz, 3H, CH_3_); ^13^C NMR (100 MHz, CDCl_3_): δ 175.43, 149.31, 148.69, 146.88, 145.85, 144.44, 132.15, 130.15, 129.88, 125.94, 123.79, 122.66, 120.69, 116.25, 113.04, 61.30(CH), 44.53(CH_2_), 44.50(CH_2_), 31.22(CH_2_), 20.27(CH_2_), 18.70(CH_3_), 16.63(CH_3_), 13.8(CH_3_); MS = 450.1838, *m/z* = 451.2311 [M + 1]^+^

### 3.22. Cell Viability Assay

MCF-7 cells were procured from Procell Life Science and Technology Co. LTD, Wuhan, China. Human breast cancer T47D, BT474, and SK-BR-3 cells were obtained from American Type Culture Collection (Washington, DC, NW, USA). Cells (2000) were cultured in MEM or Leibovitz’s L-15 medium enriched with 2% FBS and maintained in a humidified atmosphere of 5% CO_2_ at 37 °C. A stock solution of DMSO-dissolved compounds was prepared, and this solution was diluted with culture medium as required. MCF-7 Cells (4 × 10^3^) were cultured in 96-well plates for 12 h and then treated for 72 h with compounds at concentrations of 0, 0.01, 0.1, 10, 100, and 1000 mM. Using Alamar Blue, the compounds were evaluated for their inhibitory effects [44,45]

### 3.23. Preparation of Whole Cell Lysates

As previously reported, whole-cell lysates from cells treated with compound **3f** were prepared to detect protein expression and phosphorylation [46] using a lysis buffer [Tris (20 mM, pH 7.4), NaCl (250 mM), EDTA (2 mM, pH 8.0), Triton X-100 (0.1%), aprotinin (0.01 mg/mL), leupeptin (0.005 mg/mL), phenylmethane sulfonyl fluoride (0.4 mM), and NaVO4 (4 mM)]. To remove insoluble material, lysates were centrifuged at 13,000 rpm for 15 min.

### 3.24. Western Blot Analysis

Equal protein concentrations of cell lysate were resolved on sodium dodecyl sulfate-polyacrylamide gel electrophoresis, followed by transfer to nitrocellulose membranes as reported previously [47]. Incubation was carried out overnight at 4 °C with antibodies after treatment with 5% skim milk. Afterward, the membranes were washed, probed with HRP-conjugated secondary antibodies for 2 h, and then visualized using chemiluminescence.

### 3.25. In Silico DFT Calculations

The theoretical calculations were performed using Gaussian 09 [48] and Gaussview 5 program. The polarized and diffused basis set 6-311+G(d, p) provides accurate values for all theoretical calculations. The computational studies utilized the most useful and precise hybrid method of B3LYP [49]. The structures of the compounds were fully optimized with no constraint. The global chemical reactivity descriptors (GCRD) were evaluated to understand the chemical properties of a molecule, such as ionization potential, electron affinity, chemical hardness (*η*), softness (S), potential (*μ*), electronegativity (*χ*), and electrophilic index (ψ). The global hardness [*η* = (E_LUMO_ − E_HOMO_)/2], softness (S = 1/2*η*), chemical potential [*μ* = (E_HOMO_ + E_LUMO_)/2], electronegativity [*χ* = (I+A)/2], and electrophilic index (ψ *= μ*^2^/2*η*) were calculated by taking the energies of HOMO as ionization potential (I) and LUMO as electron affinity (A). Chemical hardness, softness, and potential were used to understand the chemical reactivity of the molecular system [50].

### 3.26. Docking Simulation

AUTODOCK4.0 [51] software was employed for molecular docking studies. The docking receptor STAT3 (PDB ID: 1BG1) was retrieved from the RCSB Protein Data Bank. The graphical user interface AUTODOCK TOOLS was utilized to build up the protein molecule. The water molecules were removed from the protein crystal and only polar hydrogens were applied. The predicted gasteigers charge was found to be −25.9962. For both dockings, the grid box size was 127 × 127 × 85 with a grid spacing of 0.55Å. The receptor and the complex were saved in the pdbqt file format. Using Lamarckian genetic algorithm searches, twenty runs were performed. The default parameters were employed, with a maximum of 2.5 × 10^6^ energy assessments and an initial population of 50 randomly placed individuals [52]. The autogrid4.exe and autodock4.exe functions were executed at the end of the docking process to generate glg and dlg files. Maestro (v2020.4) [53] and PyMOL (v2.5.2) [54] were used to generate the interaction pictures and visualization plots.

### 3.27. Immunocytochemistry Assay

As described earlier, STAT3 phosphorylation in cells was quantified [55]. After compound **3f** treatment (10 μM for 4 h), cells were fixed for 20 min with paraformaldehyde (4%). Thereafter, cells were treated with 0.2% Triton X-100 in phosphate-buffered saline for permeabilization, followed by blocking with 5% bovine serum albumin for 1 h. Then, the preparation was incubated overnight at 4 °C with a rabbit polyclonal anti-human STAT3 antibody (dilution, 1:100). The next day, slides were subjected to washing and incubation with Alexa Fluor 594 (dilution, 1:1000) anti-Rabbit IgG1 for 1 h at room temperature in the dark. In the next step, DAPI (5 μg/mL) was used for counterstaining the nuclei. The slides were mounted and analyzed under an Olympus FluoView FV1000 confocal microscope (Tokyo, Japan).

## 4. Conclusions

A series of imidazopyridine-tethered-purazoles were synthesized and screened for loss of viability of breast cancer cells. The lead compound **3f** inhibited STAT3 phosphorylation in MCF-7 and T47D cells. The DFT calculations and molecular docking experiments showed a theoretical bioactivity correlation for compound **3f** towards STAT3. In conclusion, compound **3f** effectively inhibited the phosphorylation of STAT3 in MCF-7 and T47D cells, indicating that ITPs may be an alternative method to target STAT3 in BC.

## Figures and Tables

**Table 1:** Physical data of 2-pyrazolines.

Compound Code	R_1_ or R_2_	Melting Point in °C	Yield in %
3a	Piperidone	170–172	97
3b	1-Boc piperazine	166–168	96
3c	Piperazine	176–178	35
3d	1-acetyl piperazine	208–210	98
3e	1-(4-chlorophenyl)piperazine	180–182	96
3f	1-(2,3-dichlorophenyl)piperizine	122–124	95
3g	1-(4-chloro-2-fluorophenyl)piperazine	196–198	95
3h	1-(3,4-difluorophenyl)piperazine	192–194	96
3i	1-(2-fluorophenyl)piperazine	160–162	98
4a	Aniline	210–212	98
4b	4-nitrophenylaniline	230–232	95
4c	Cyclohexylamine	204–206	98
4d	4-chlorophenylaniline	240–242	98
4e	3-chlorophenylaniline	242–244	98
4f	4-methylphenylaniline	256–258	97
4g	3,5-bis(triflourophenyl)aniline	230–232	98%
4h	n-butylamine	246–248	96%

**Table 2:** Effect of newly synthesized imidazopyridine-tethered pyrazolines on various breast cancer/carcinoma cell viability.

Entry	IC50 (μM)				
	MCF-7	T47D	BT-474	SK-BR-3	MCF-10A
2	>100	>100	97.82±1.76	>100	>100
3a	22.81 ± 1.36	23.31 ± 1.28	4.109 ± 0.41	>100	>100
3b	22.29 ± 1.35	15.15 ± 1.13	21.29 ± 1.32	28.28 ± 1.34	>100
3c	29.27 ± 1.47	39.95 ± 1.49	26.75 ± 1.37	95.17 ± 1.79	>100
3d	21.03 ± 1.32	>100	8.04 ± 0.47	32.14 ± 1.46	>100
3e	13.24 ± 1.12	29.9 ± 1.05	17.6 ± 0.87	39.13 ± 1.46	38.34 ± 1.44
3f	9.27 ± 0.97	23.51 ± 1.25	13.91 ± 1.32	>100	>100
3g	19.40 ± 1.29	86.55 ± 1.95	63.16 ± 1.75	99.96 ± 1.98	8.69 ± 0.69
3h	19.56 ± 1.29	12.5 ± 1.01	3.061 ± 0.32	38.81 ± 1.42	6.27 ± 0.26
3i	63.69 ± 1.80	>100	>100	>100	>100
4a	81.37 ± 1.91	>100	>100	>100	>100
4b	65.36 ± 1.82	45.61 ± 1.43	7.259 ± 0.54	69.02 ± 1.41	>100
4c	46.08 ± 1.66	>100	52.83 ± 1.54	>100	>100
4d	>100	>100	>100	>100	>100
4e	>100	>100	>100	>100	>100
4f	56.92 ± 1.39	>100	>100	>100	>100
4g	10.90 ± 1.01	>100	>100	>100	>100
4h	>100	54.42 ± 1.68	>100	>100	90.59 ± 1.98
Olaparib	3.28 ± 0.68				

**Table 3:** FMOs energy with global chemical parameters values of synthesized compounds.

Global Parameters	3f	3h	3i	4c	4d	4g
E_HOMO_ (eV)	−5.764	−5.799	−5.704	−5.645	−5.733	−5.905
E_LUMO_ (eV)	−3.049	−3.086	−3.016	−3.201	−3.267	−3.366
ΔE_LUMO–HOMO_ (eV)	2.715	2.713	2.688	2.444	2.466	2.539
Ionization potential (*I*) (eV)	5.764	5.799	5.704	5.645	5.733	5.905
Electron affinity (A) (eV)	3.049	3.086	3.016	3.201	3.267	3.366
Hardness (*η*) (eV)	1.357	1.356	1.344	1.222	1.233	1.269
Softness (S) (eV)^−1^	0.368	0.368	0.372	0.409	0.405	0.393
Chemical potential (μ) (eV)	−4.406	−4.442	−4.360	−4.423	−4.500	−4.635
Electronegativity (χ) (eV)	4.406	4.442	4.360	4.423	4.500	4.635
Electrophilicity (ψ) (eV)	7.151	7.274	7.072	8.004	8.211	8.463

## Data Availability

Not applicable.

## Supplementary Materials

The following are available online at https://www.mdpi.com/article/10.3390/bioengineering10020159/s1. Supplementary data for newly synthesized molecules and their IC50 values determined against human breast cancer cells.

## Sample Availability

The synthesized compounds are available at Basappa Laboratory.
