# Supplementary material for: De Novo Design of Imidazopyridine-Tethered Pyrazolines That Target Phosphorylation of STAT3 in Human Breast Cancer Cells
Source: Bioengineering (Basel). 2023 Jan 24;10(2):159. doi: 10.3390/bioengineering10020159 (PMC9952374; doi:10.3390/bioengineering10020159)
Supplement: Supplementary file 1 [file bioengineering-10-00159-s001.zip › bioengineering-2130275-supplementary.pdf]

*De novo* drug design of imidazopyridine tethered pyrazoles that Targets Phosphorylation of STAT3 in Human Breast Cancer Cells

Akshay Ravish[1,#], Rashmi Shivakumar[1,#], Zhang Xi[2,#], Min Hee Yang[3], Ji-Rui Yang[4], Ananda Swamynayaka[5], Omantheswara Nagaraja[5], Mahendra Madegowda[5], Arunachalam Chinnathambi[6], Sulaiman Ali Alharbi[6], Vijay Pandey[4,7], Gautam Sethi[8,*], Kwang Seok Ahn[2,9,*], Peter E. Lobie[3,4,7,*], Basappa Basappa[1*]

**Supplementary data for newly synthesized molecules and their IC50 values determined against human breast cancer cells**

**NMR DATA**

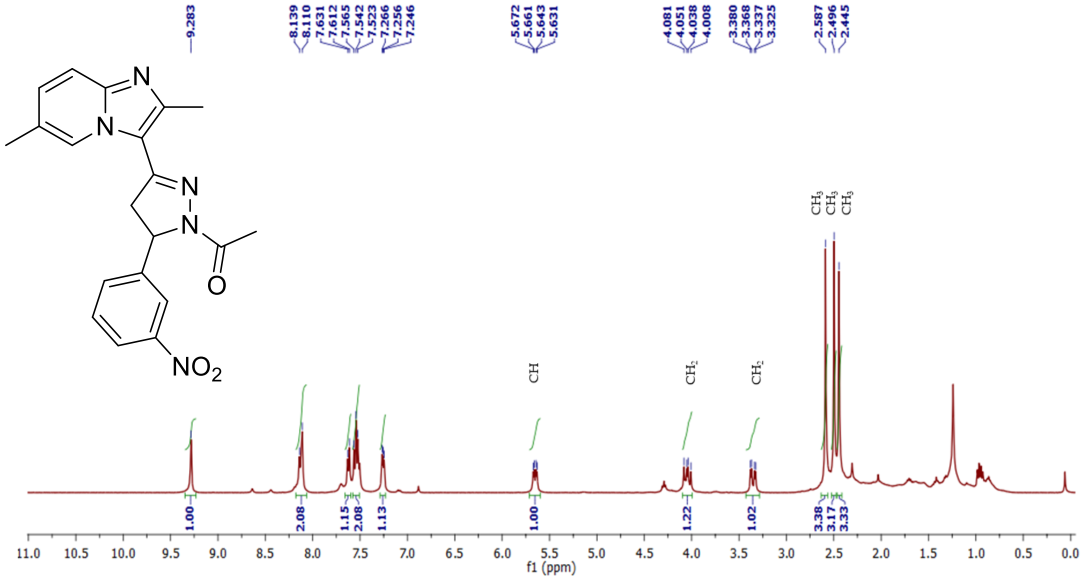

$^1$H NMR of **2**

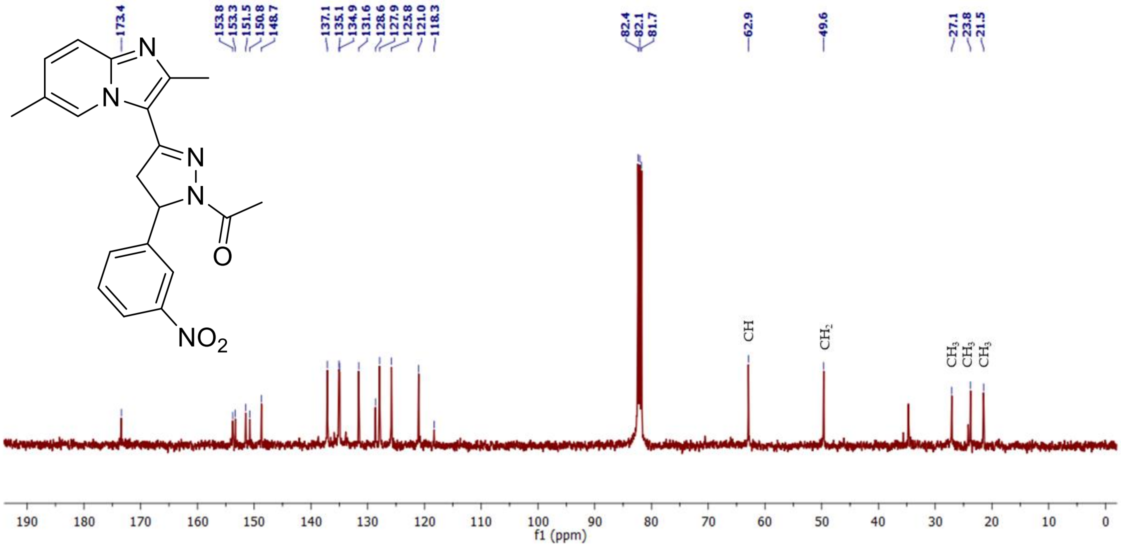

$^{13}$H NMR of **2**

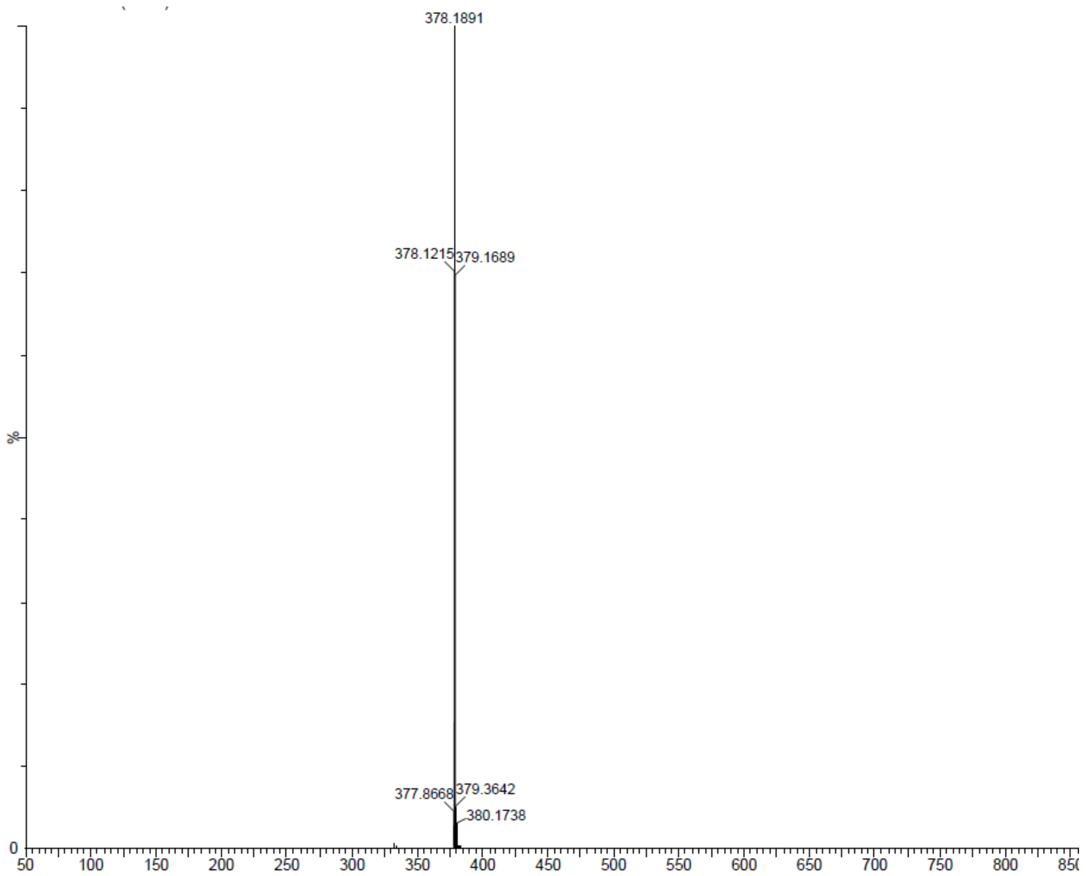


Mass spectra of **2**

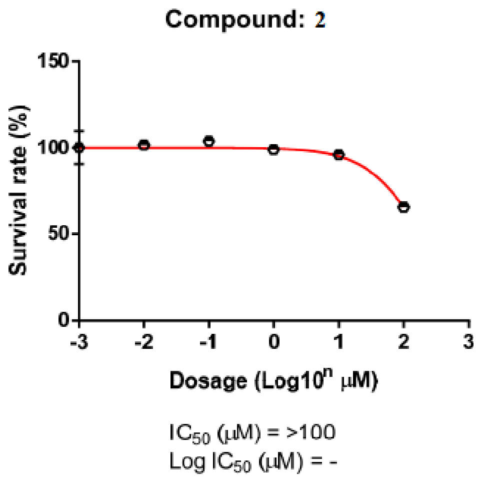


- **Cell line: MCF7 (2000 cells/per well[96])**
- **Treated time: 72hrs**
- **Assay: MTT (90mins incubated)**
- **Data:**

| Conc. (μM) | Viability | |
|---|---|---|
| | AVE. | ± SD. |
| **0** | 100.00 | 6.79 |
| **0.01** | 101.59 | 1.41 |
| **0.1** | 103.75 | 0.44 |
| **1** | 98.89 | 0.47 |
| **10** | 95.88 | 1.28 |
| **100** | 65.53 | 1.39 |

MCF-7 Log curve of **2**

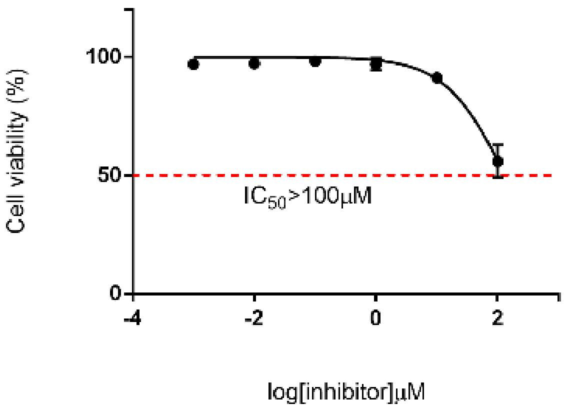


T47D Log curve of 2

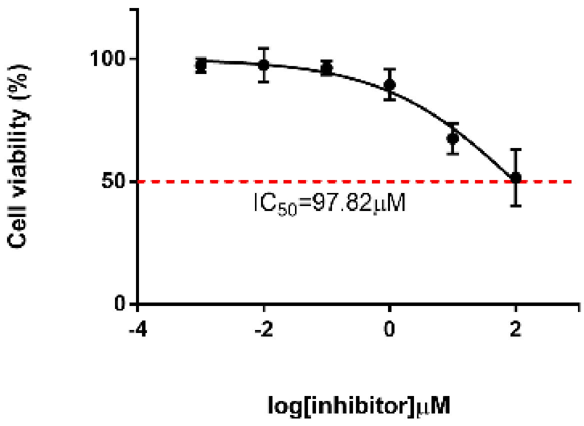


BT474 Log curve of 2

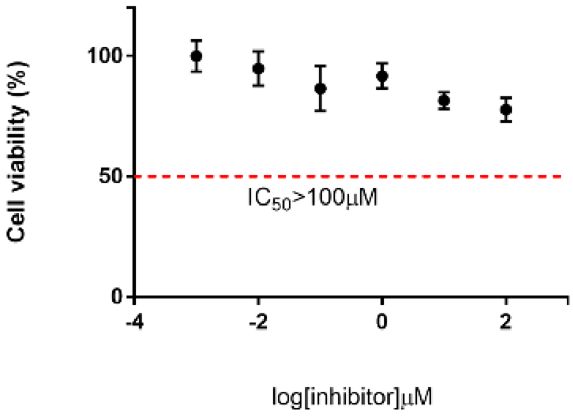


SK-BR-3 log curve of 2

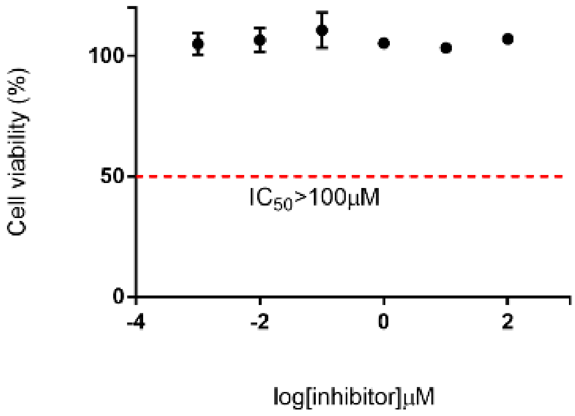


MCF-10A log curve of 2

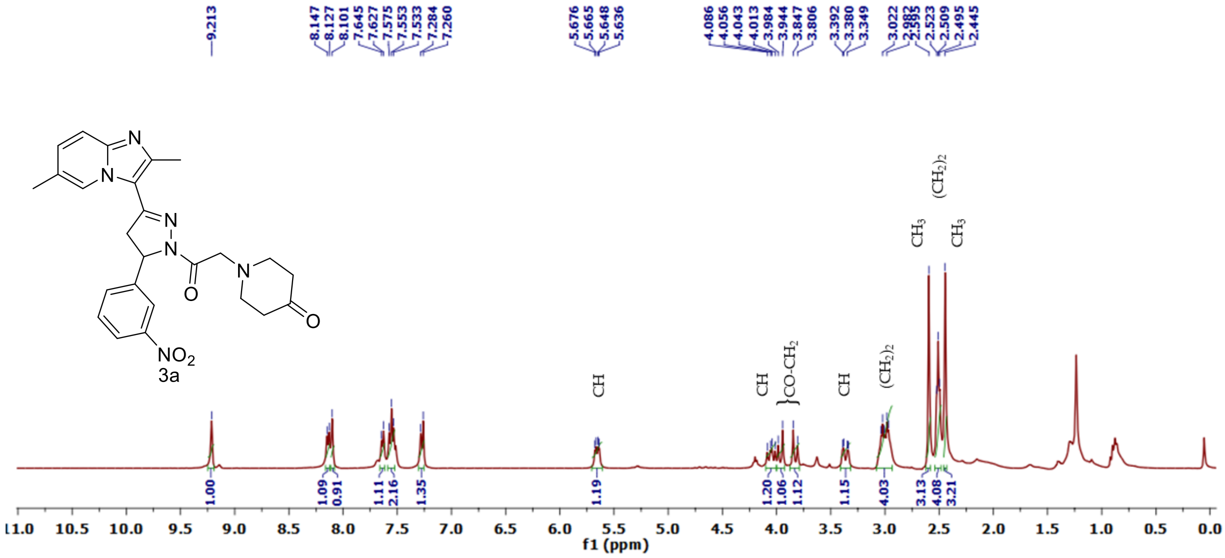


$^1$H NMR of **3a**

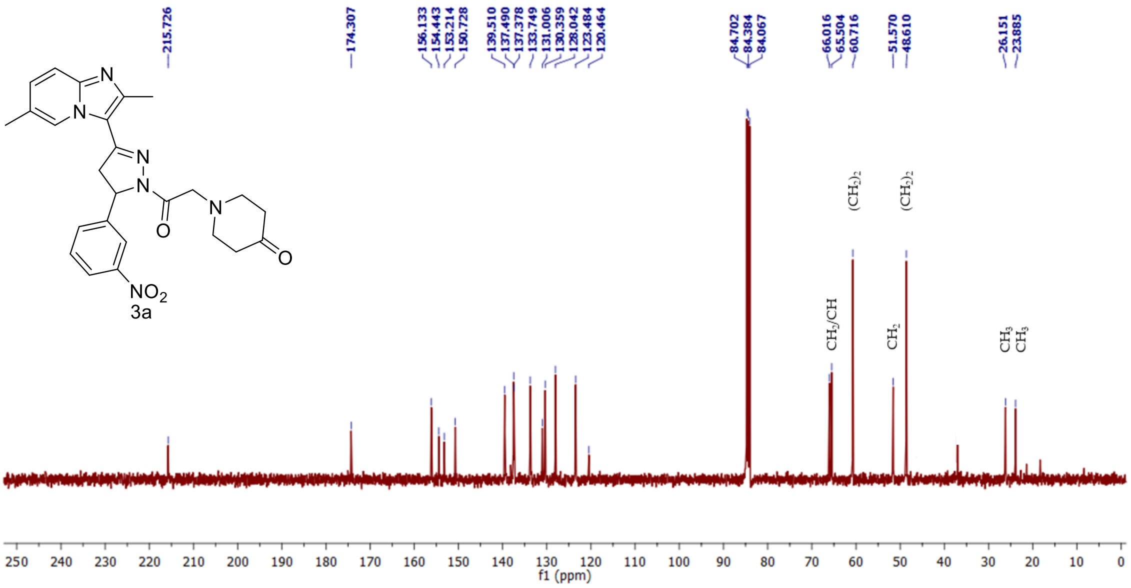


13H NMR of **3a**

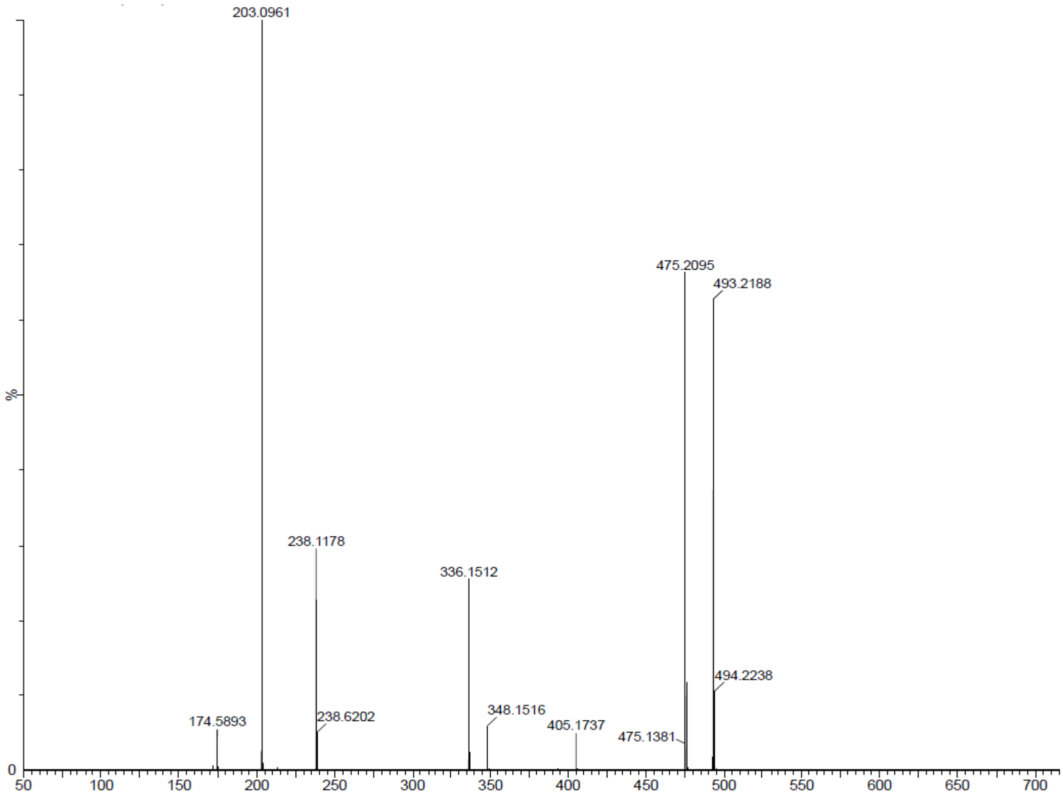


Mass spectra of **3a**

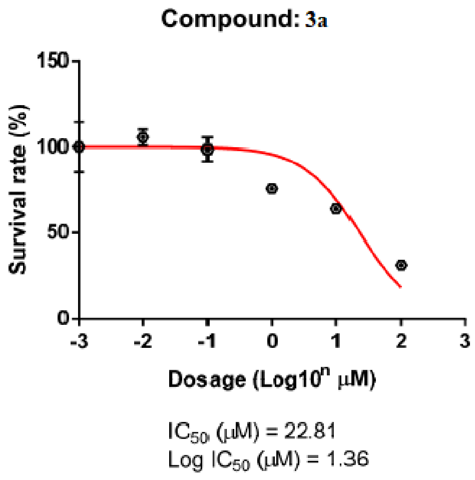


- Cell line: MCF7 (2000 cells/per well[96])
- Treated time: 72hrs
- Assay: MTT (90mins incubated)
- Data: 3a

| Conc. (μM) | Viability | |
|---|---|---|
| | AVE. | ± SD. |
| 0 | 100.00 | 10.10 |
| 0.01 | 105.50 | 4.69 |
| 0.1 | 98.61 | 6.78 |
| 1 | 75.99 | 1.24 |
| 10 | 64.01 | 0.42 |
| 100 | 31.59 | 0.04 |

MCF-7 Log curve of **3a**

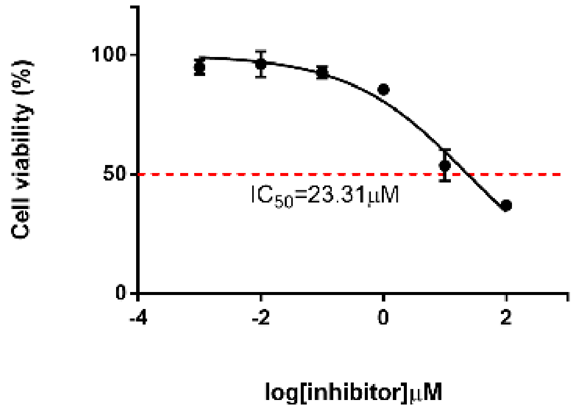


T47D Log curve of 3a

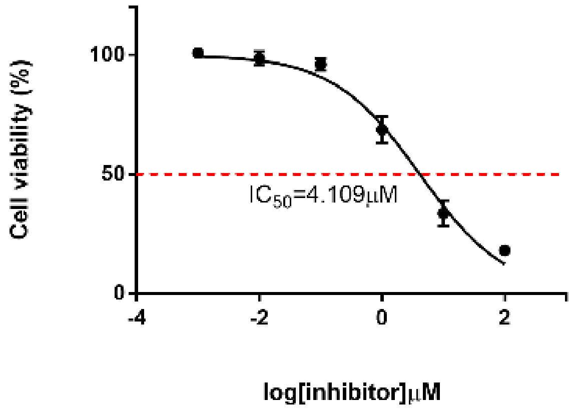


BT474 Log curve of 3a

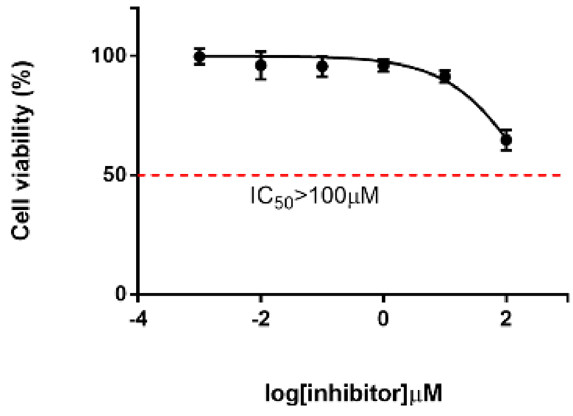


SK-BR-3 log curve of 3a

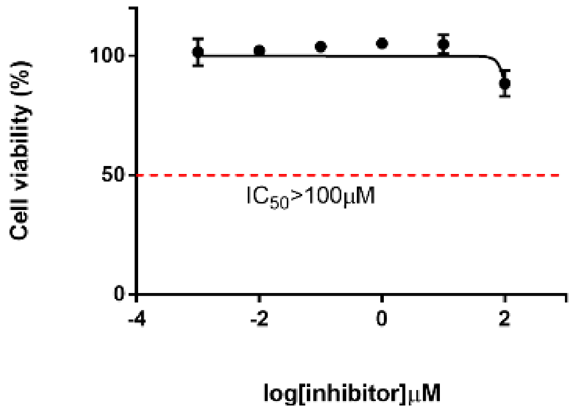


MCF-10A log curve of 3a

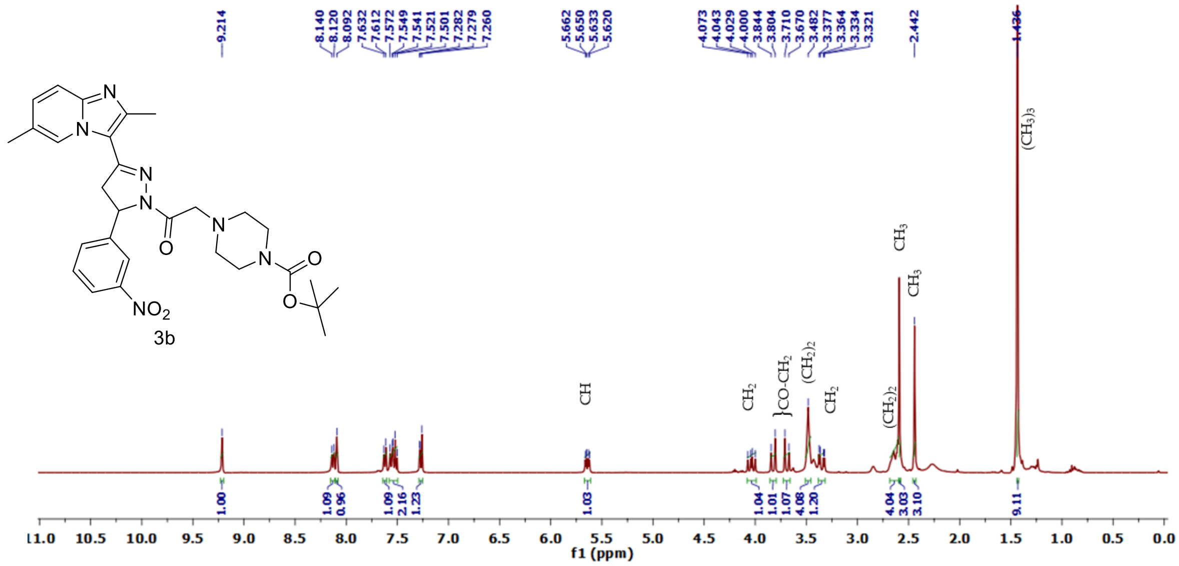


$^{1}H$ NMR of **3b**

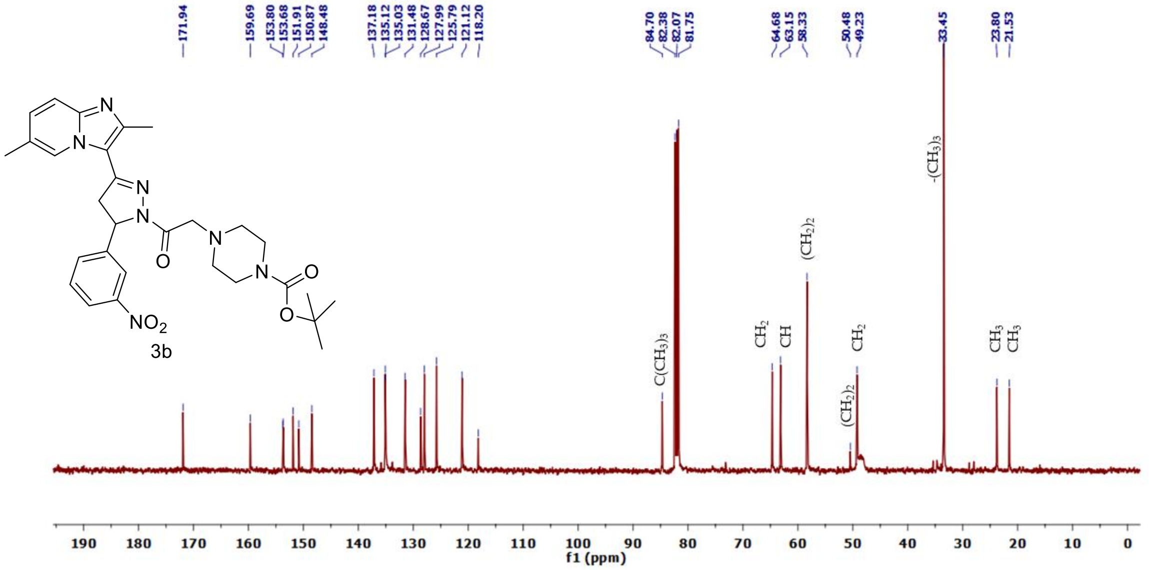


$^{13}C$ NMR of **3b**

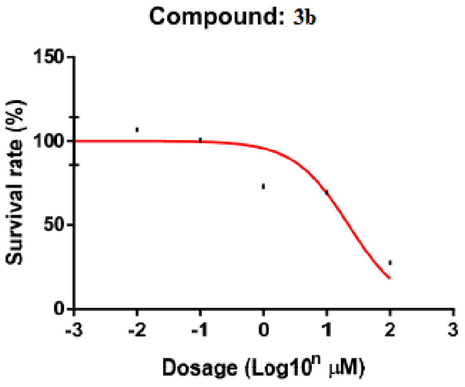


$IC_{50}$ (μM) = 22.29
Log $IC_{50}$ (μM) = 1.35

- Cell line: MCF7 (2000 cells/per well[96])
- Treated time: 72hrs
- Assay: MTT (90mins incubated)
- Data: 3b

| Conc. (μM) | Viability AVE. | Viability ± SD. |
|---|---|---|
| **0** | 100.00 | 10.10 |
| **0.01** | 105.31 | 0.45 |
| **0.1** | 99.02 | 0.39 |
| **1** | 71.53 | 1.51 |
| **10** | 68.11 | 2.70 |
| **100** | 26.22 | 0.70 |

MCF-7 Log curve of **3b**

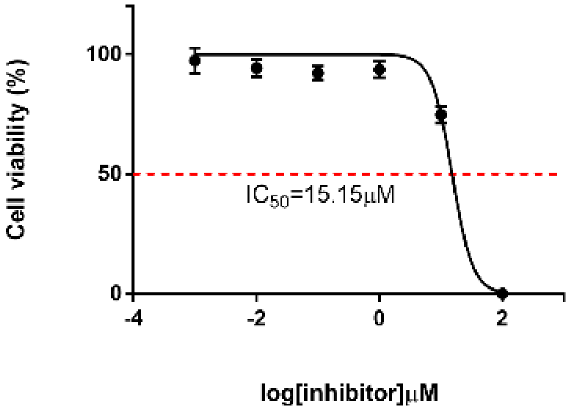


T47D Log curve of 3b

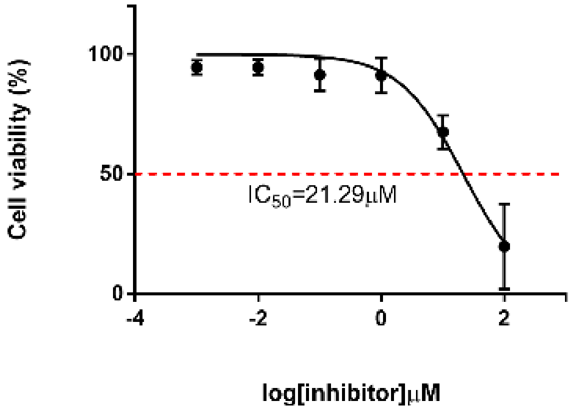


BT474 Log curve of 3b

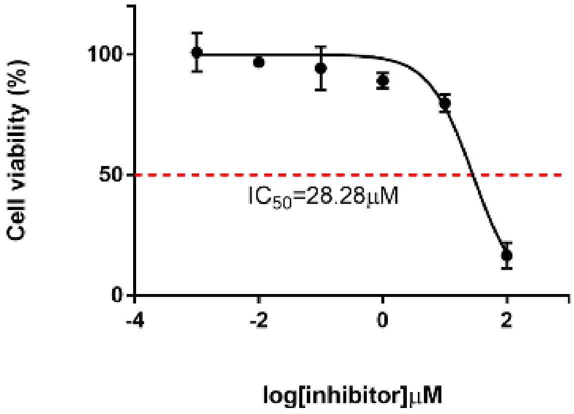


SK-BR-3 log curve of 3b

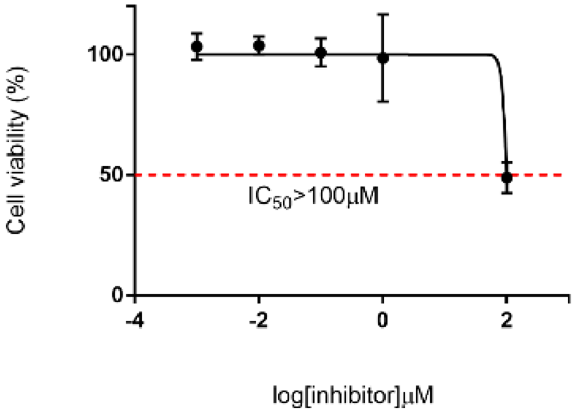


MCF-10A log curve of 3b

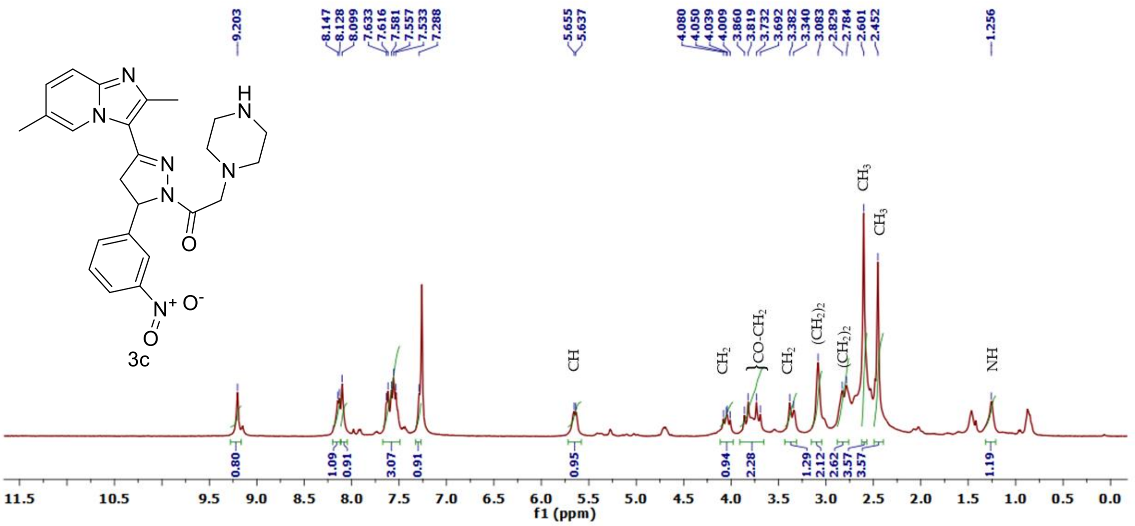


$^{1}$H NMR of **3c**

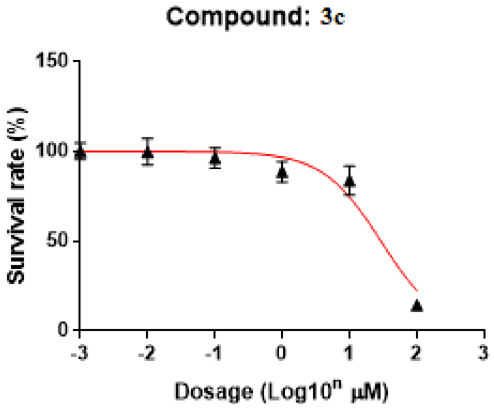


IC$_{50}$ (μM) = 29.27
Log IC$_{50}$ (μM) = 1.47

- Cell line: MCF7 (2000 cells/per well[96])
- Treated time: 72hrs
- Assay: alamarBlue (4hrs incubated)
- Data: 3c

| Conc. (μM) | Viability | |
|---|---|---|
| | AVE. | ± SD. |
| **0** | 100.00 | 4.64 |
| **0.01** | 99.69 | 7.21 |
| **0.1** | 96.27 | 5.65 |
| **1** | 88.50 | 5.77 |
| **10** | 83.65 | 8.10 |
| **100** | 14.32 | 0.22 |

MCF-7 Log curve of **3c**

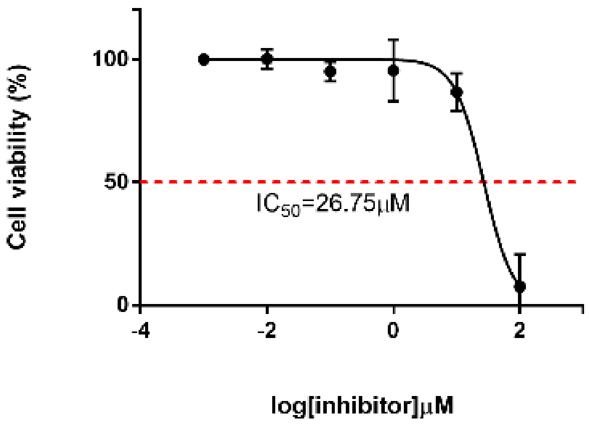


T47D Log curve of 3c

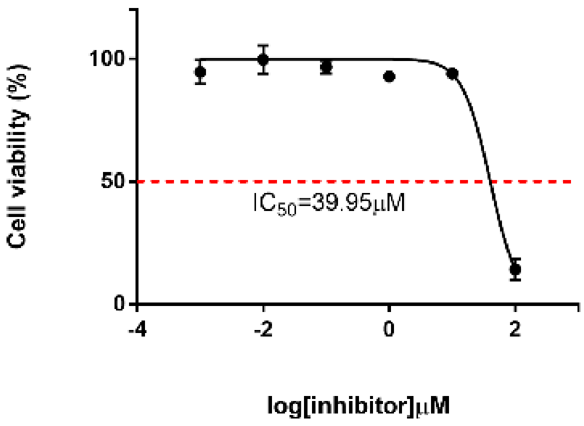


BT474 Log curve of 3c

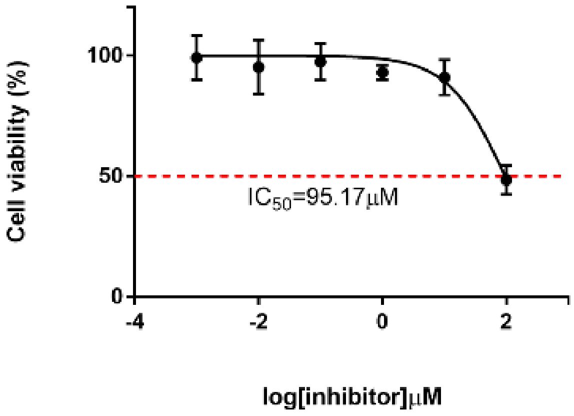


SK-BR-3 log curve of 3c

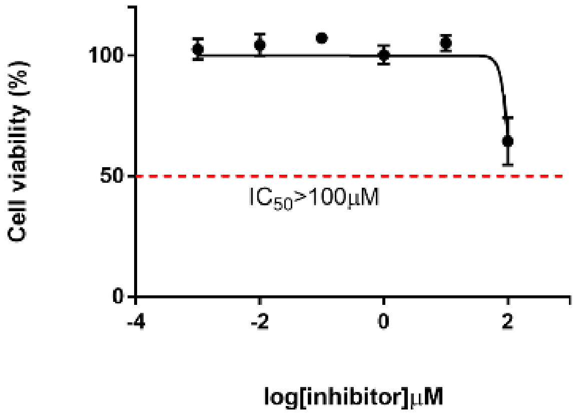


MCF-10A log curve of 3c

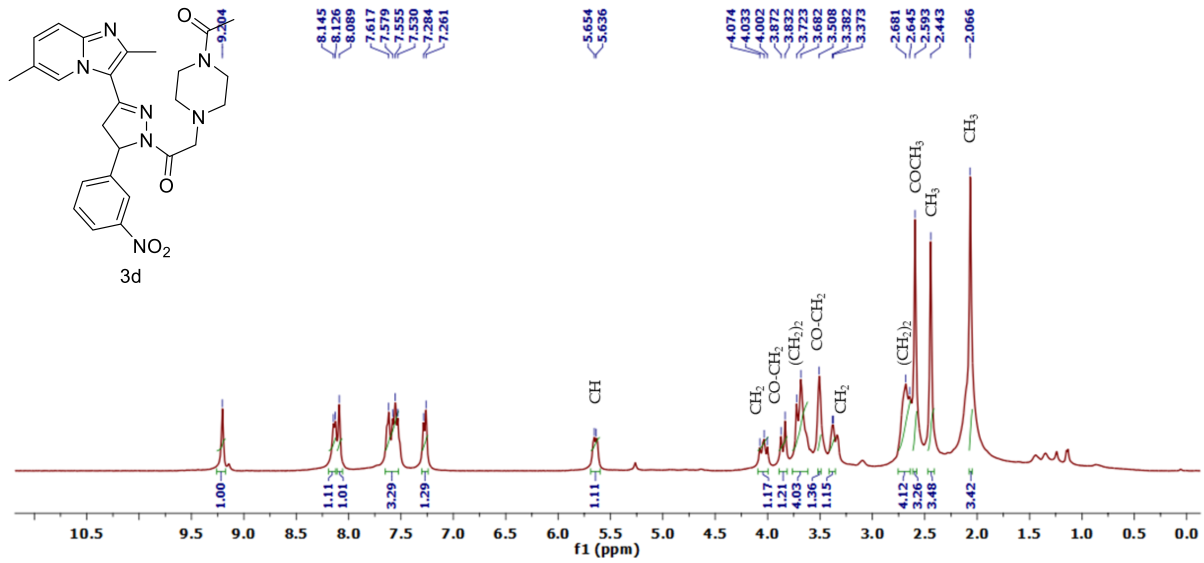


$^{1}H$ NMR of **3d**

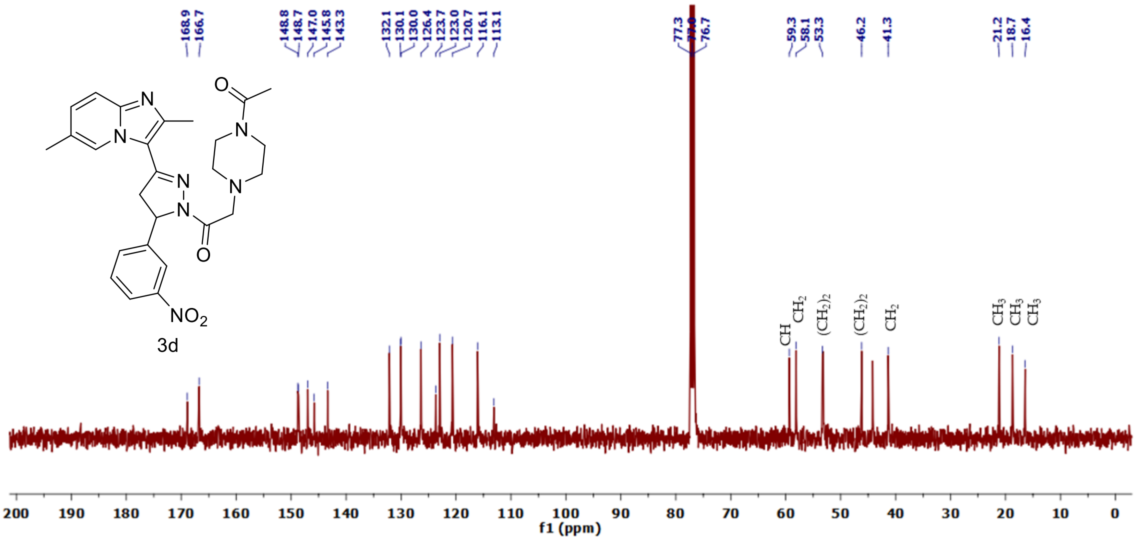


$^{13}C$ NMR of **3d**

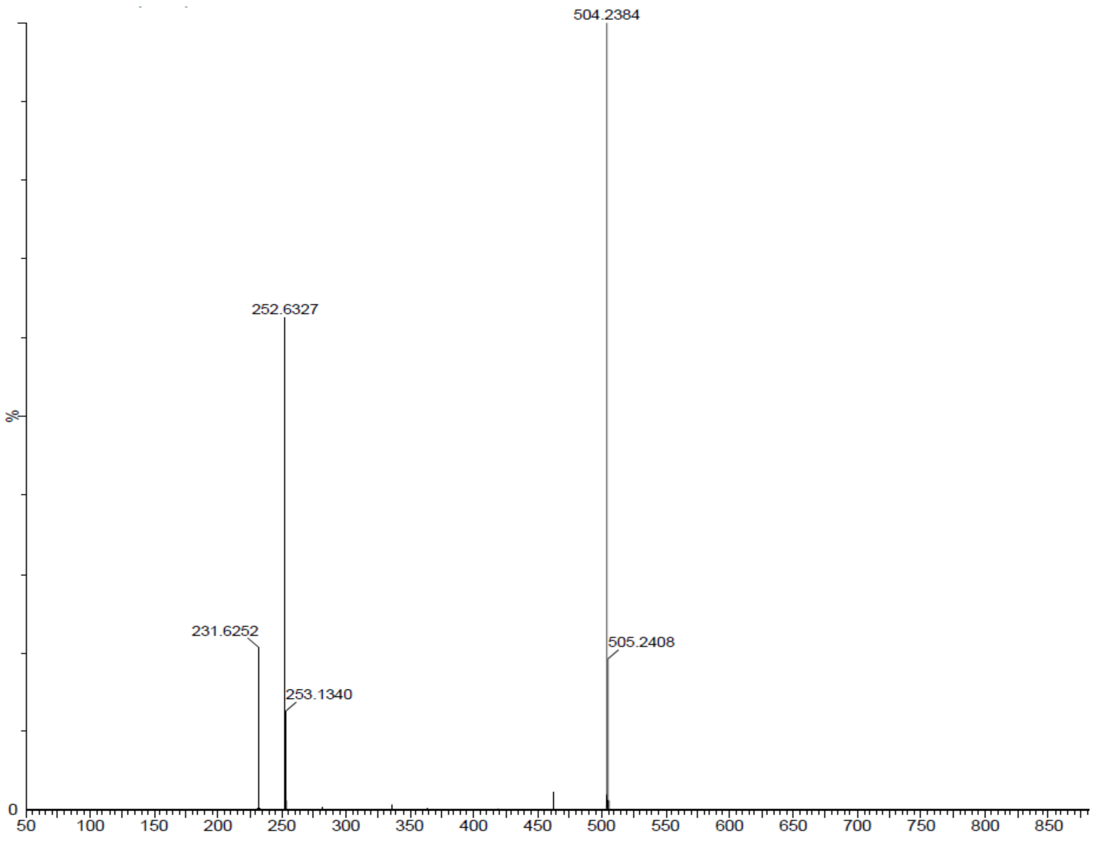


Mass spectra of **3d**

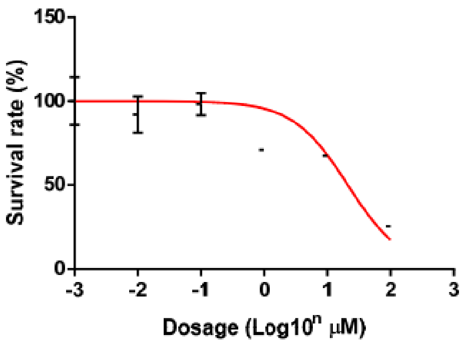


$IC_{50}$ (μM) = 21.06

Log $IC_{50}$ (μM) = 1.32

- Cell line: MCF7 (2000 cells/per well[96])
- Treated time: 72hrs
- Assay: MTT (90mins incubated)
- Data: 3d

| Conc. (μM) | Viability | |
|---|---|---|
| | AVE. | ± SD. |
| **0** | 100.00 | 10.10 |
| **0.01** | 91.97 | 10.73 |
| **0.1** | 98.15 | 6.70 |
| **1** | 70.95 | 1.91 |
| **10** | 67.40 | 0.86 |
| **100** | 25.48 | 0.05 |

MCF-7 Log curve of **3d**

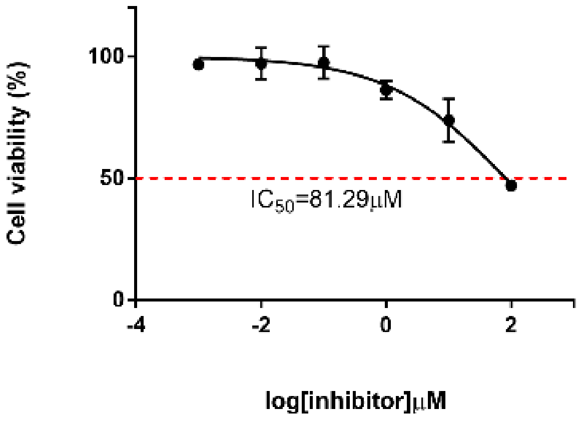


T47D Log curve of 3d

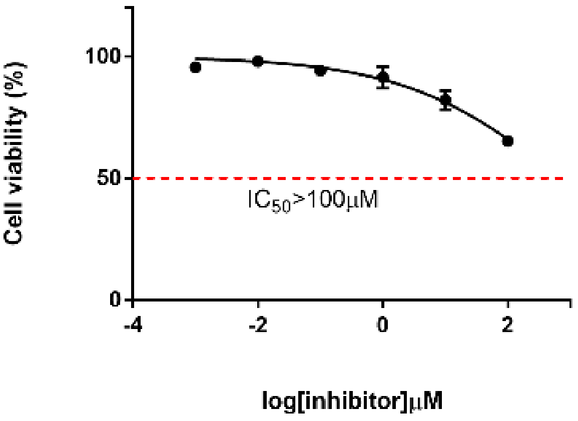


BT474 Log curve of 3d

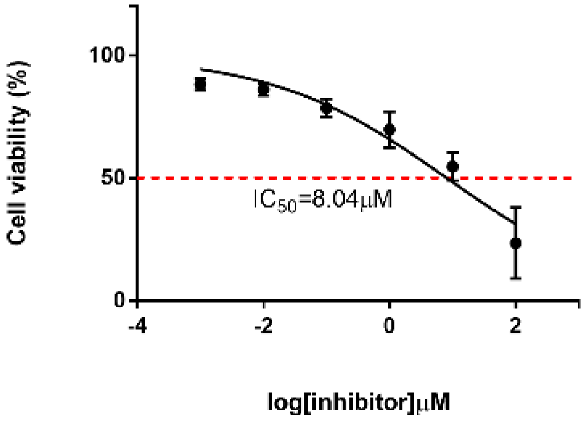


SK-BR-3 log curve of 3d

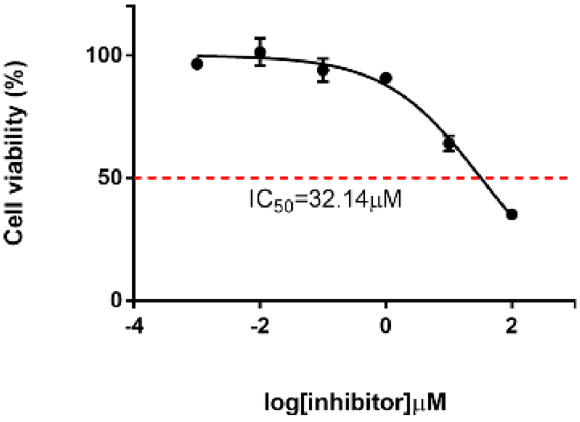


MCF-10A log curve of 3d

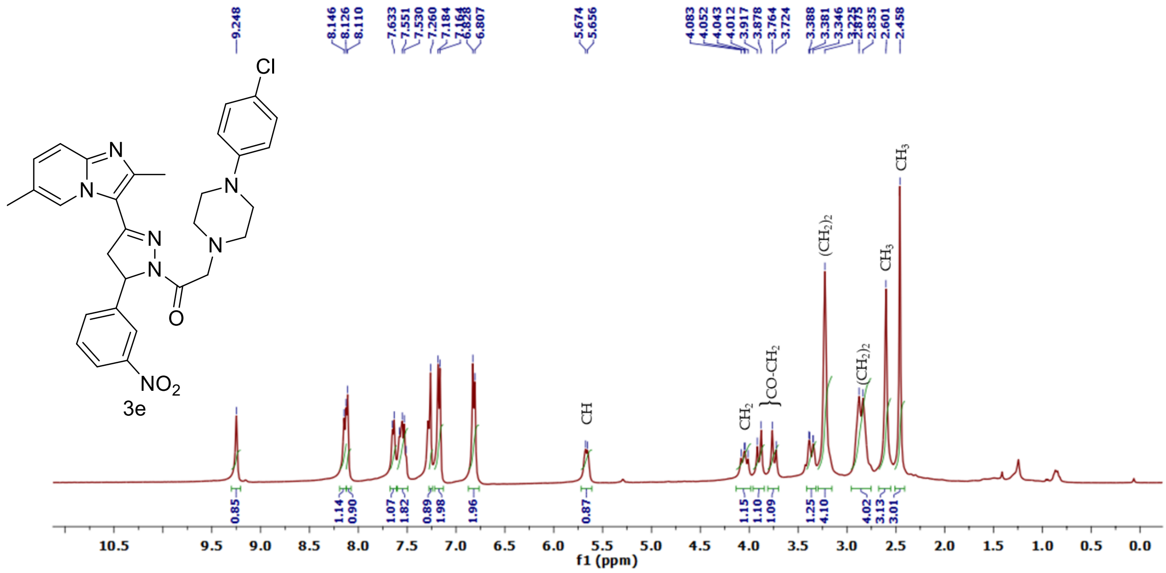


$^{1}$H NMR of **3e**

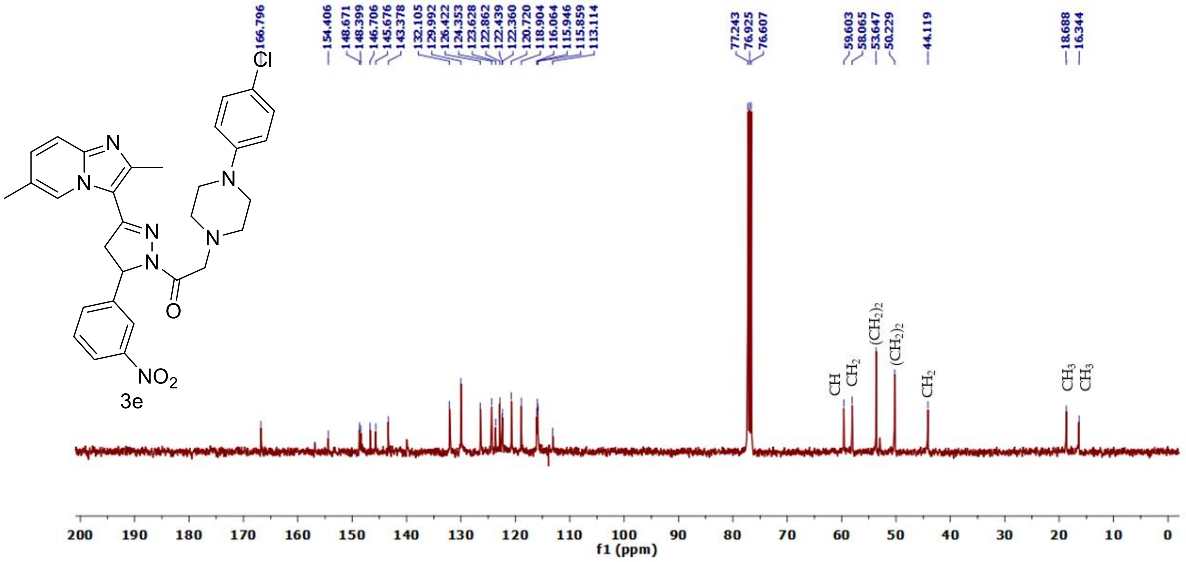


$^{13}C$ NMR of **3e**

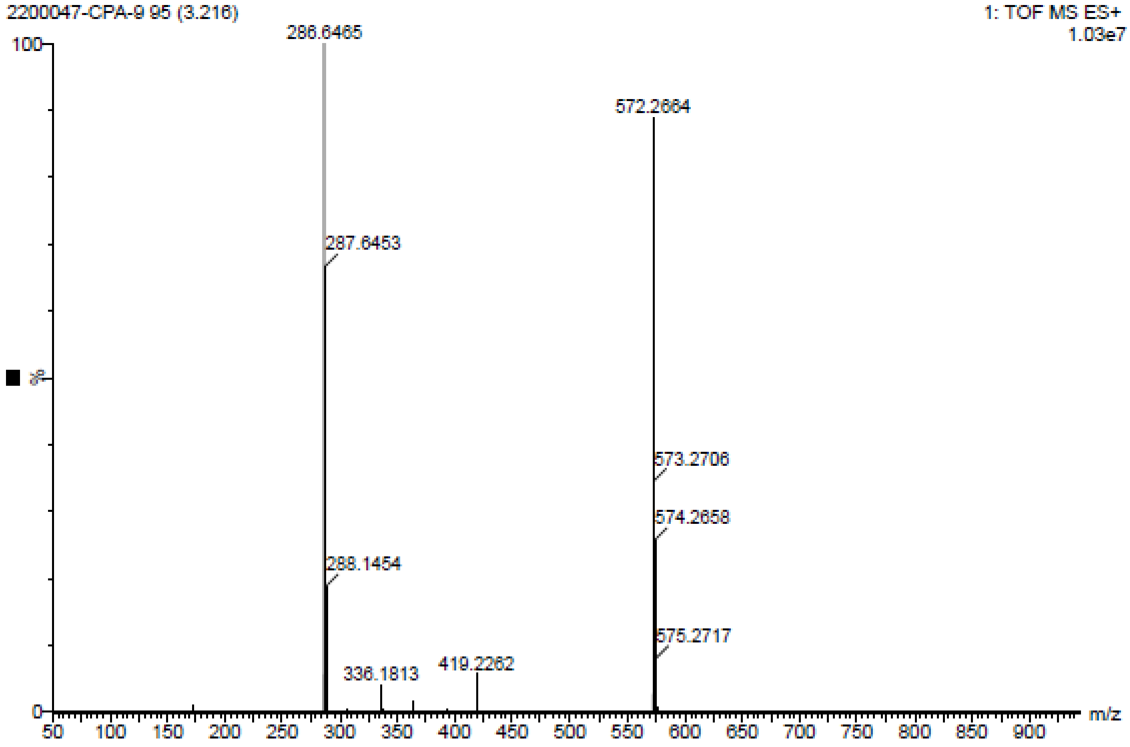


Mass spectra of compound **3e**

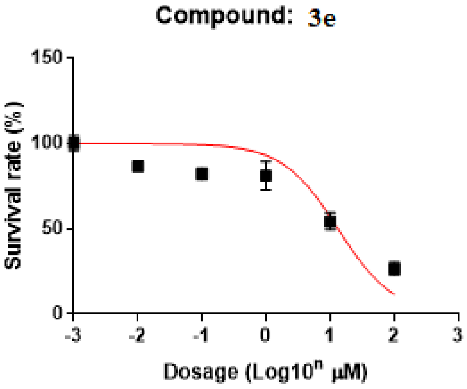


$IC_{50}$ (μM) = 13.24
Log $IC_{50}$ (μM) = 1.12

- Cell line: MCF7 (2000 cells/per well[96])
- Treated time: 72hrs
- Assay: alamarBlue (4hrs incubated)
- Data: 3e

| Conc. (μM) | Viability | |
|---|---|---|
| | AVE. | ± SD. |
| 0 | 100.00 | 4.64 |
| 0.01 | 86.60 | 1.67 |
| 0.1 | 82.25 | 3.61 |
| 1 | 81.12 | 8.26 |
| 10 | 54.43 | 4.91 |
| 100 | 26.54 | 3.76 |

MCF-7 Log curve of **3e**

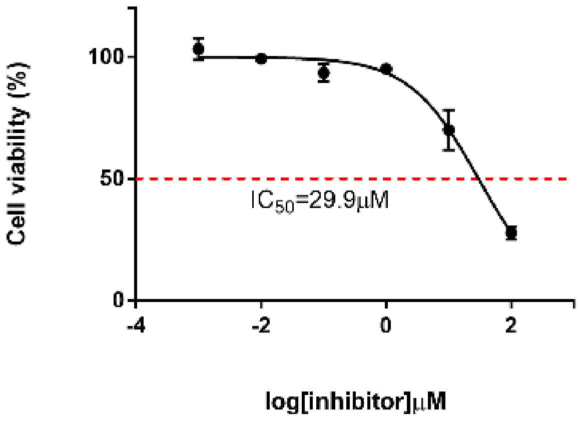


T47D Log curve of 3e

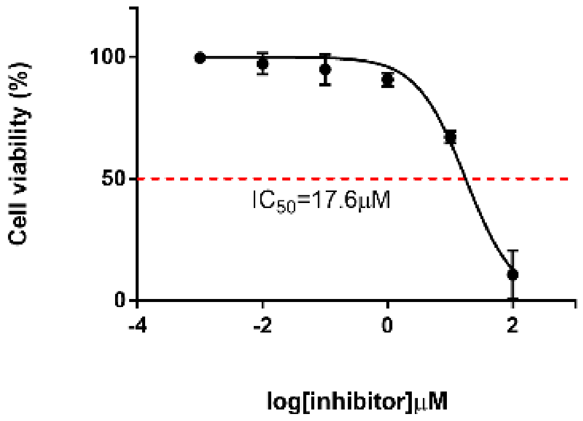


BT474 Log curve of 3e

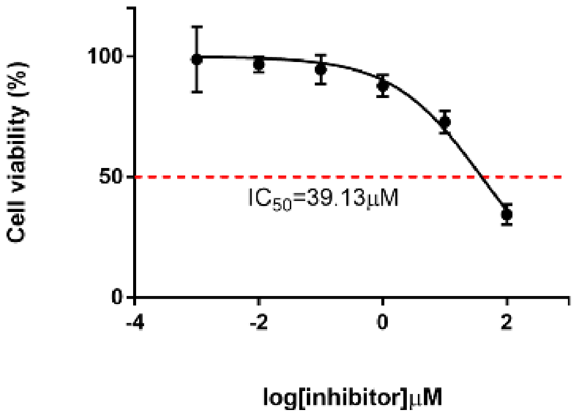


SK-BR-3 log curve of 3e

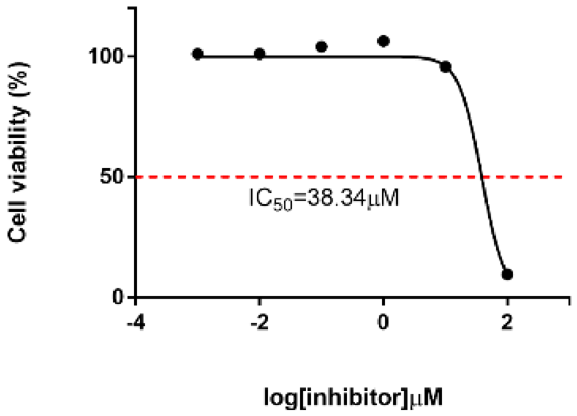


MCF-10A log curve of 3e

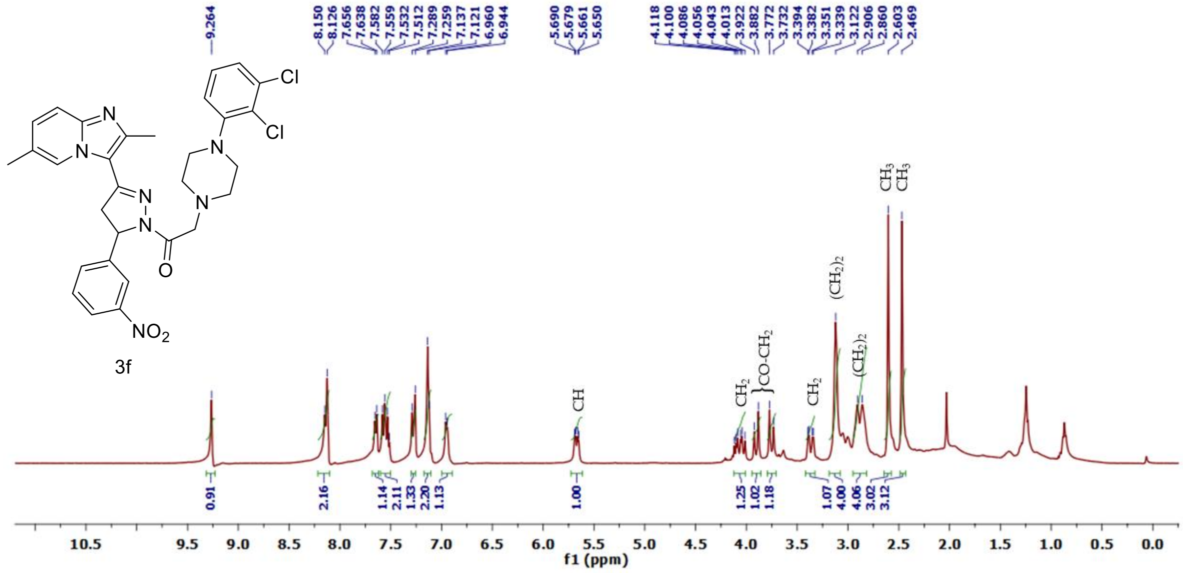

$^1$H NMR of **3f**

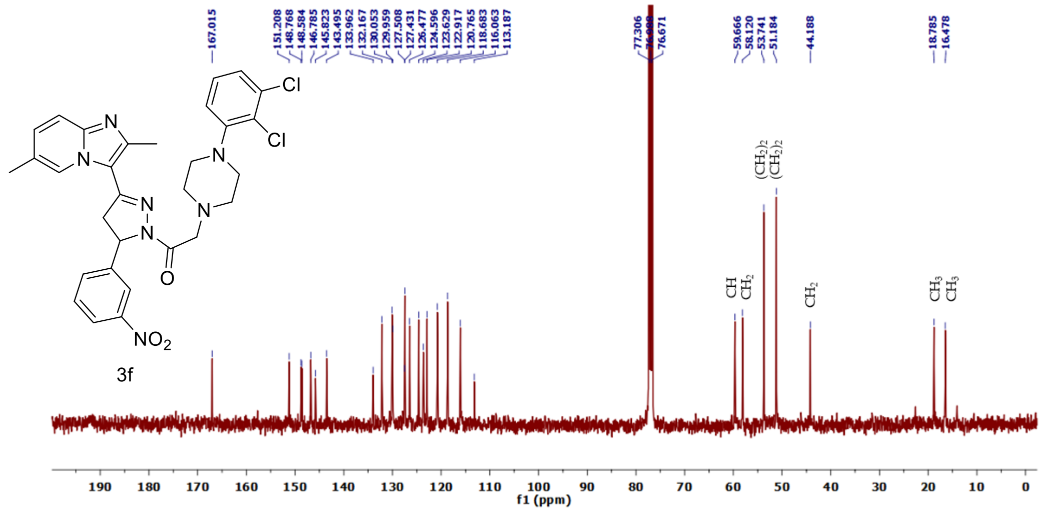

$^{13}$C NMR of **3f**

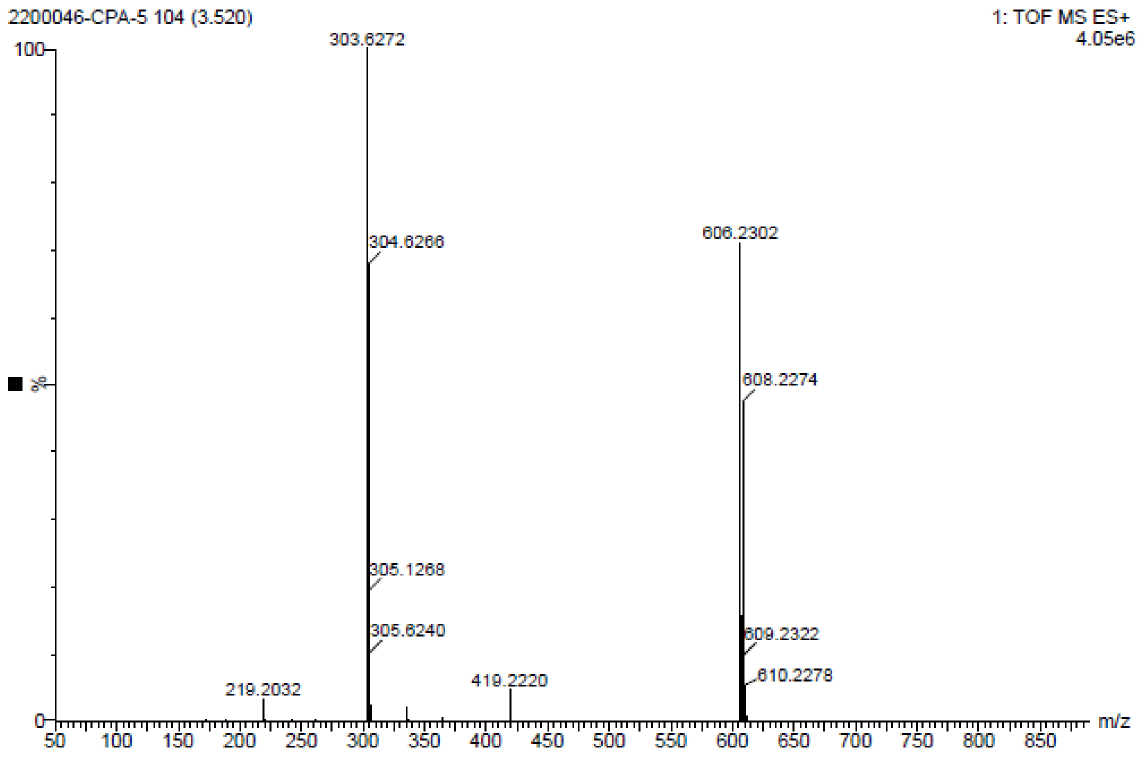


Mass spectra of **3f**

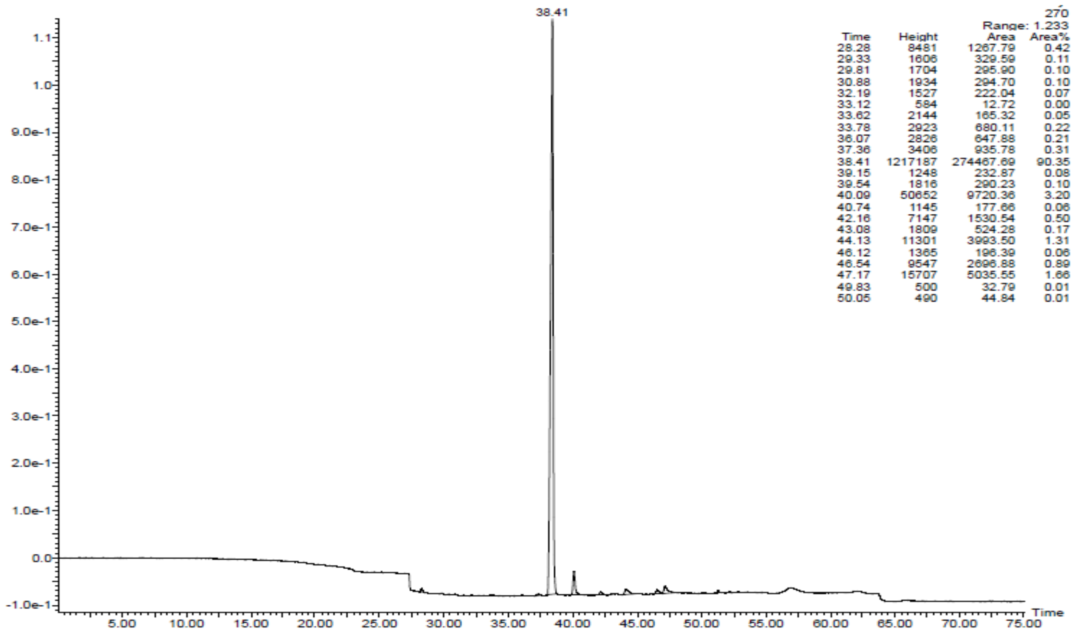


HPLC of **3f**

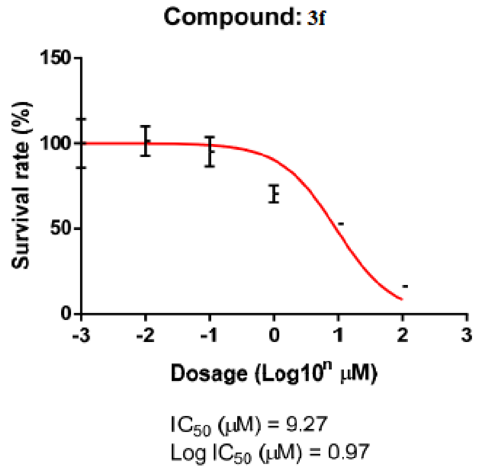


- Cell line: MCF7 (2000 cells/per well[96])
- Treated time: 72hrs
- Assay: MTT (90mins incubated)
- Data: 3f

| Conc. (μM) | Viability | |
|---|---|---|
| | AVE. | ± SD. |
| 0 | 100.00 | 10.10 |
| 0.01 | 101.30 | 8.59 |
| 0.1 | 95.19 | 8.64 |
| 1 | 70.46 | 5.03 |
| 10 | 52.84 | 1.47 |
| 100 | 16.14 | 0.59 |

MCF-7 Log curve of **3f**

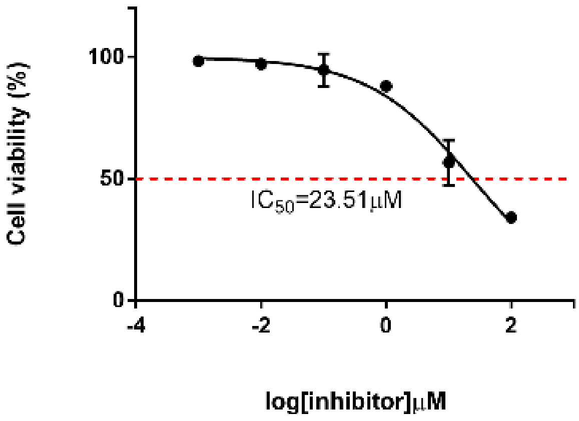


T47D Log curve of 3f

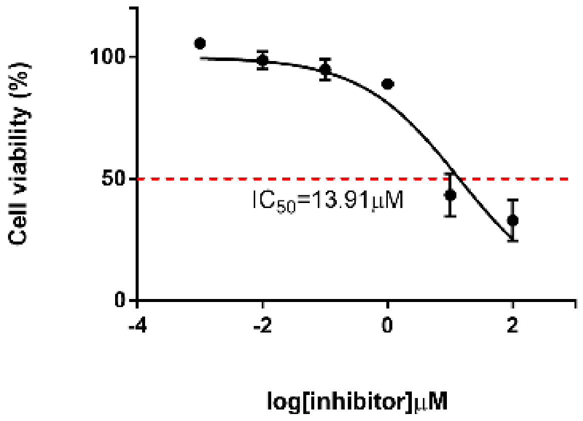


BT474 Log curve of 3f

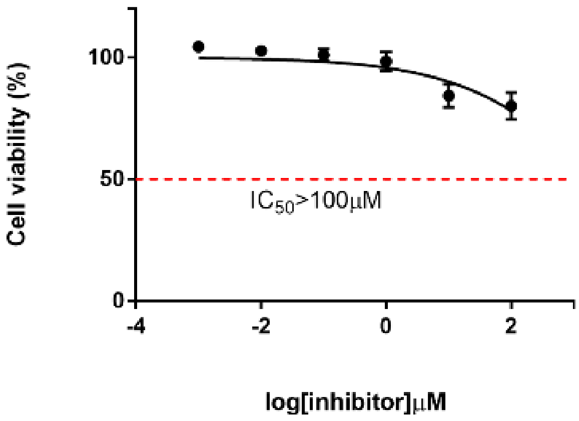


SK-BR-3 log curve of 3f

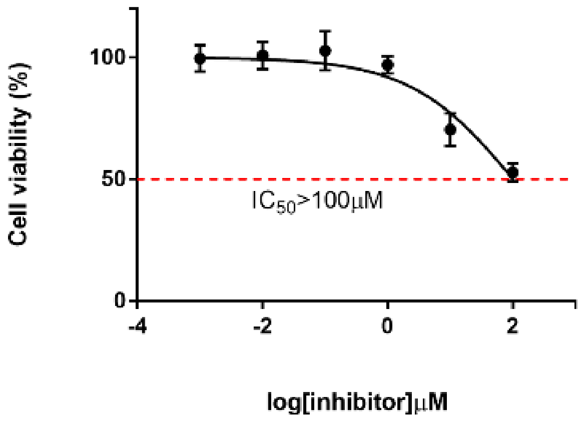


MCF-10A log curve of 3f

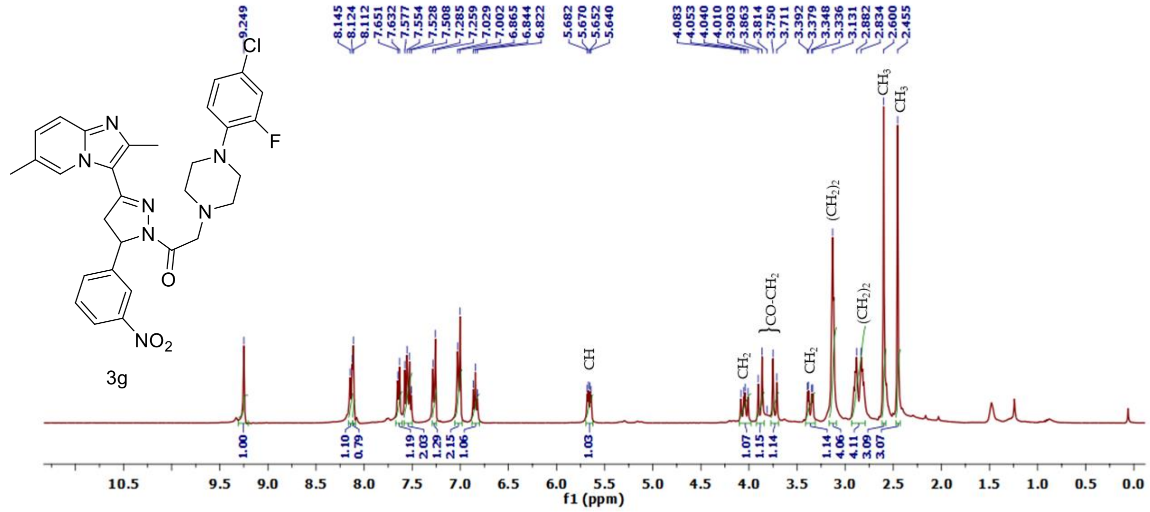

$^{1}H$ NMR of **3g**

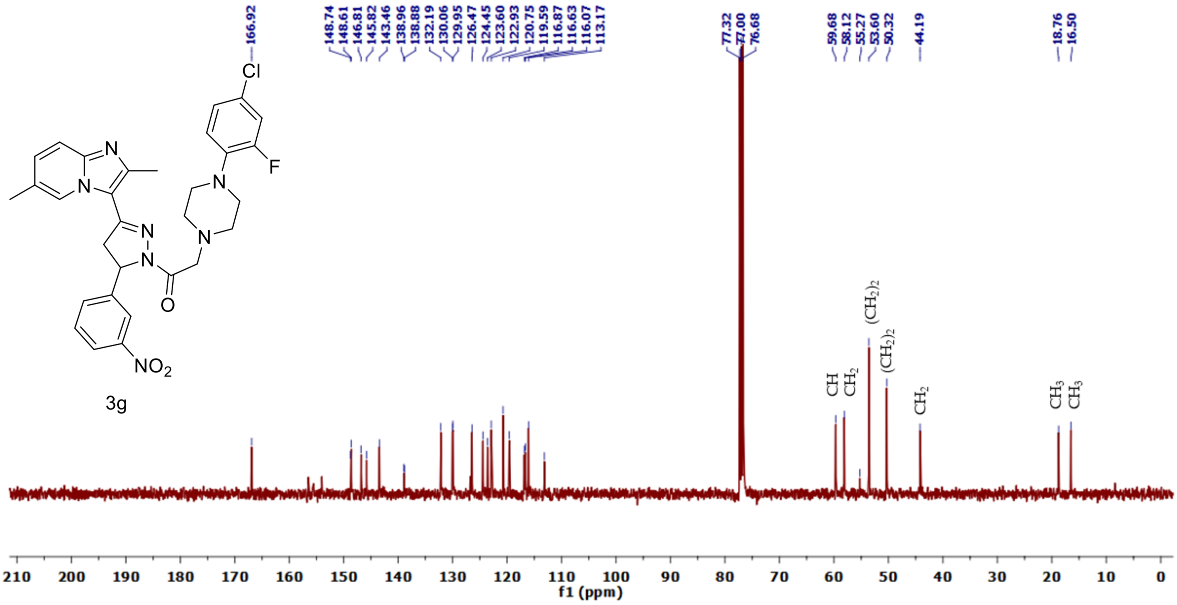

$^{13}C$ NMR of **3g**

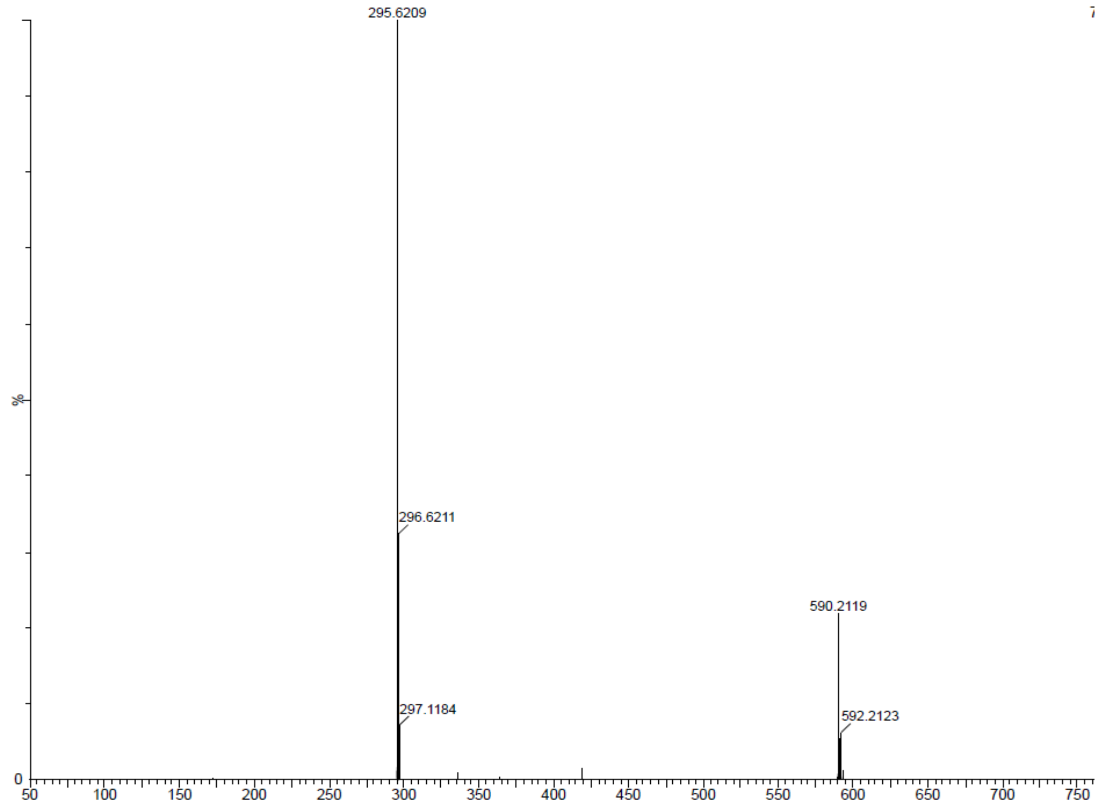


Mass spectra of **3g**

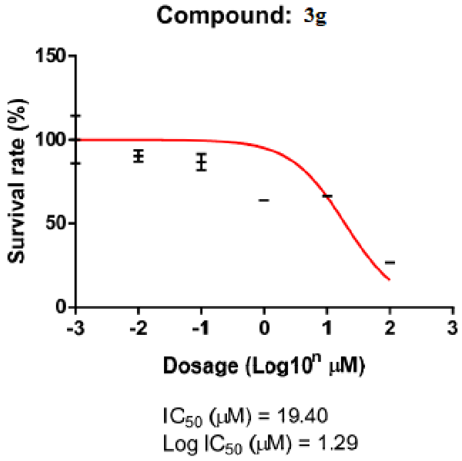


- Cell line: MCF7 (2000 cells/per well[96])
- Treated time: 72hrs
- Assay: MTT (90mins incubated)
- Data: 3g

| Conc. (μM) | Viability | |
|---|---|---|
| | AVE. | ± SD. |
| 0 | 100.00 | 10.10 |
| 0.01 | 90.30 | 3.35 |
| 0.1 | 86.63 | 4.82 |
| 1 | 63.90 | 0.27 |
| 10 | 66.48 | 1.19 |
| 100 | 26.64 | 0.08 |

MCF-7 Log curve of **3g**

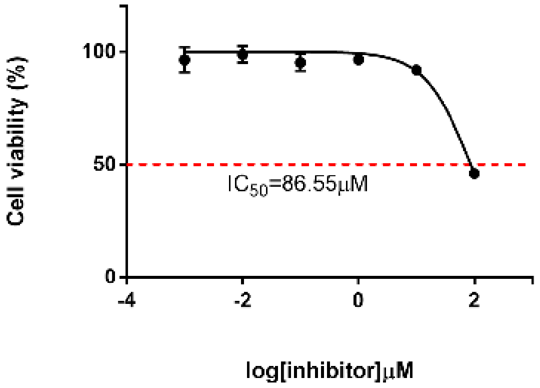


T47D Log curve of 3g

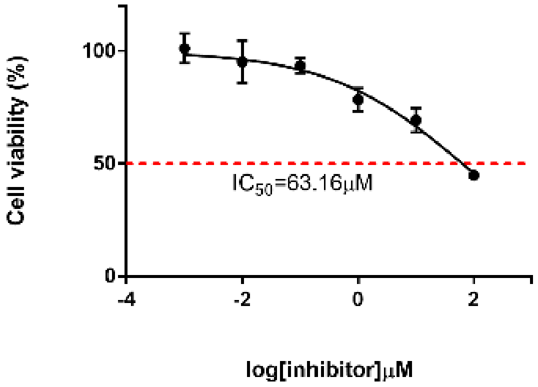


BT474 Log curve of 3g

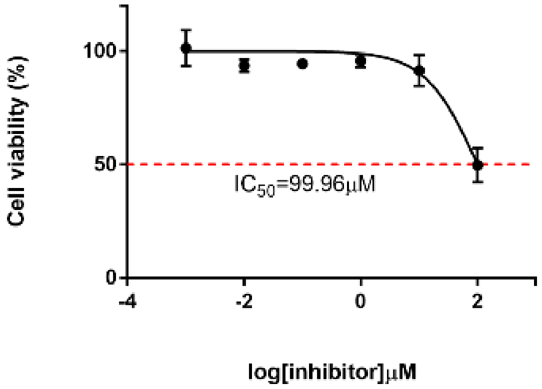


SK-BR-3 log curve of 3g

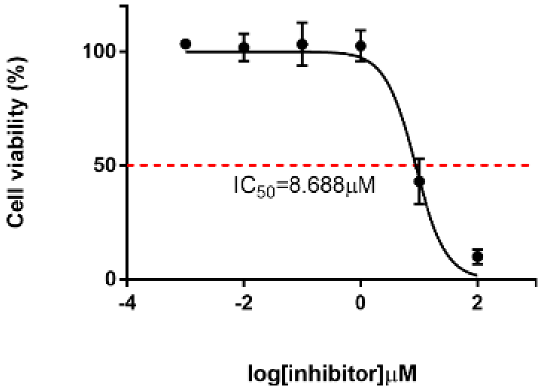


MCF-10A log curve of 3g

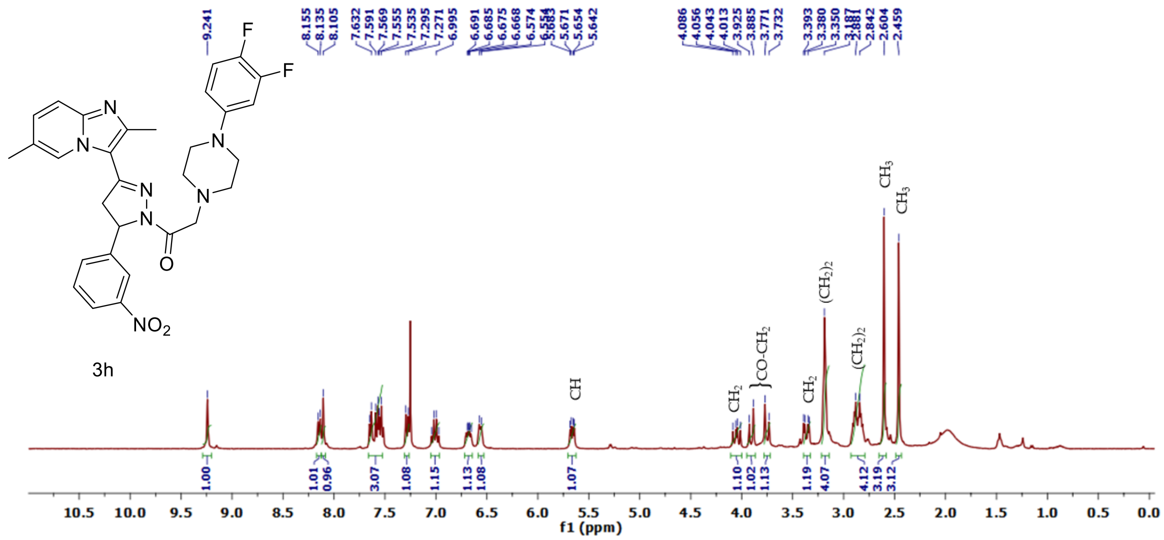

$^{1}H$ NMR of **3h**

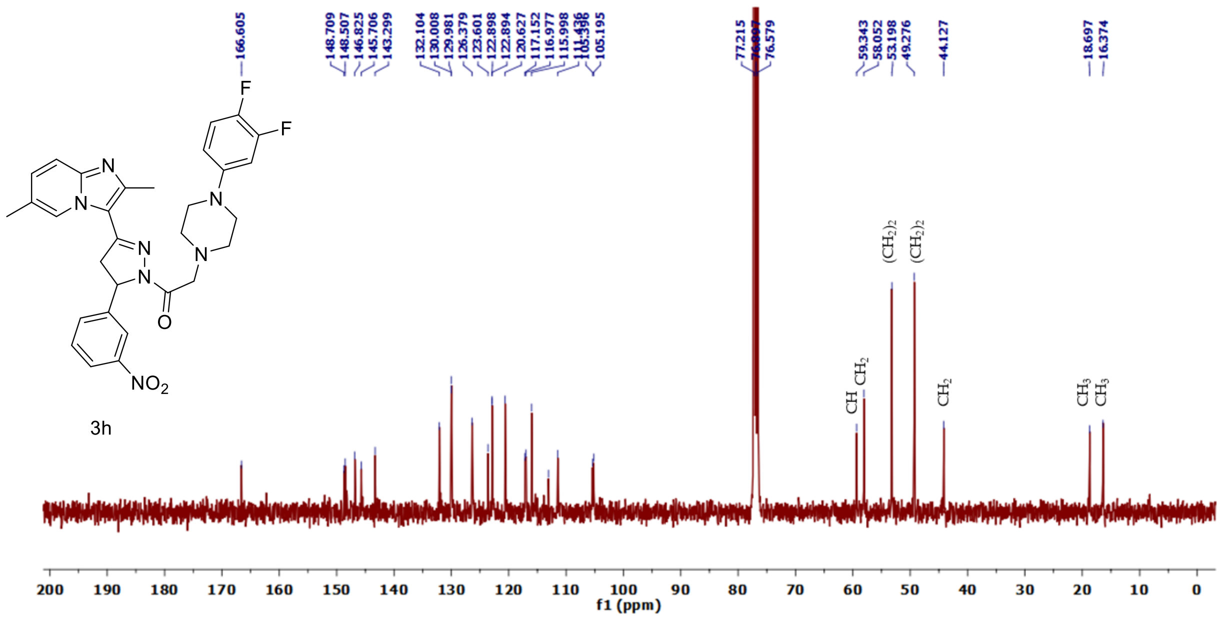

$^{13}C$ NMR of **3h**

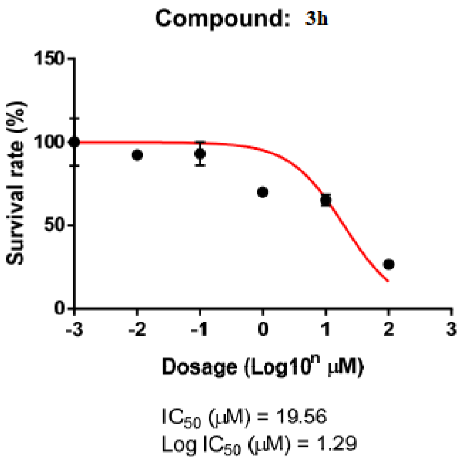


- Cell line: MCF7 (2000 cells/per well[96])
- Treated time: 72hrs
- Assay: MTT (90mins incubated)
- Data: 3h

| Conc. (μM) | Viability | |
|---|---|---|
| | AVE. | ± SD. |
| 0 | 100.00 | 10.10 |
| 0.01 | 92.34 | 0.73 |
| 0.1 | 93.00 | 7.07 |
| 1 | 70.06 | 1.19 |
| 10 | 65.18 | 3.20 |
| 100 | 26.74 | 1.54 |

MCF-7 Log curve of **3h**

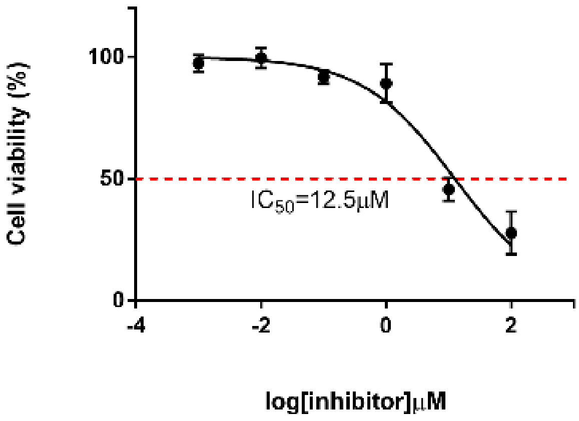


T47D Log curve of 3h

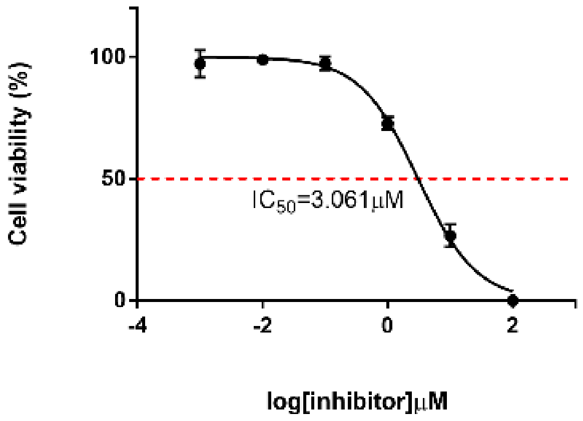


BT474 Log curve of 3h

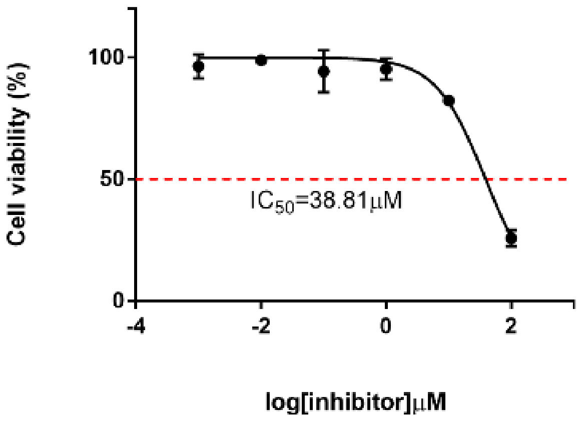


SK-BR-3 log curve of 3h

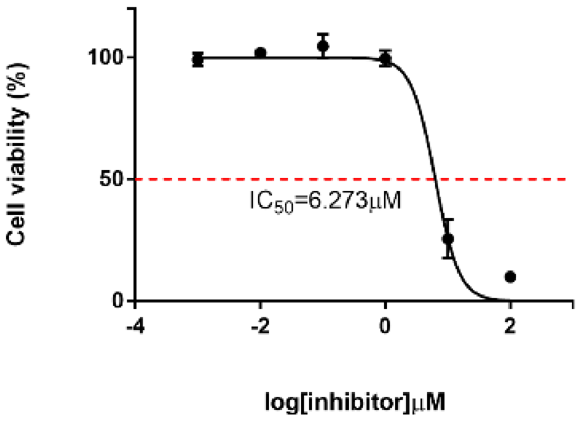


MCF-10A log curve of 3h

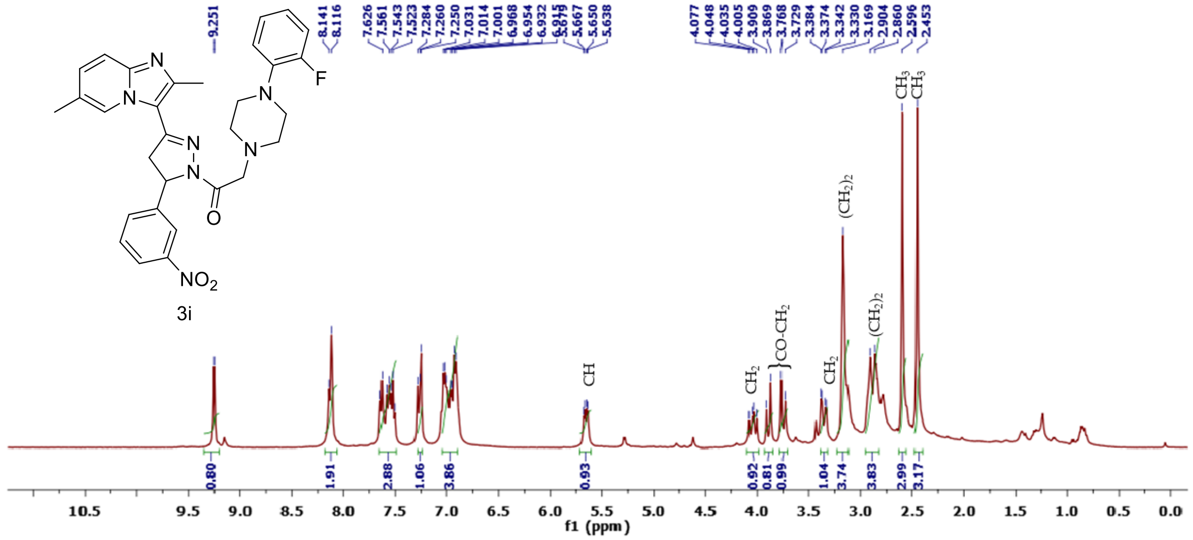


$^{1}$H NMR of **3i**

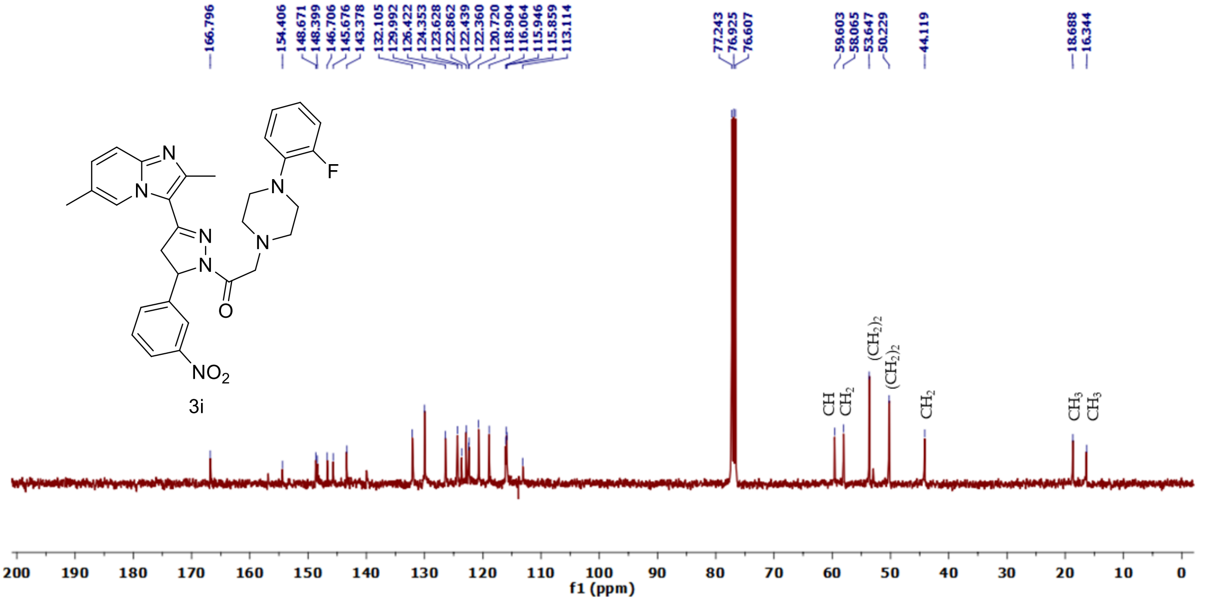


$^{13}$C NMR of **3i**

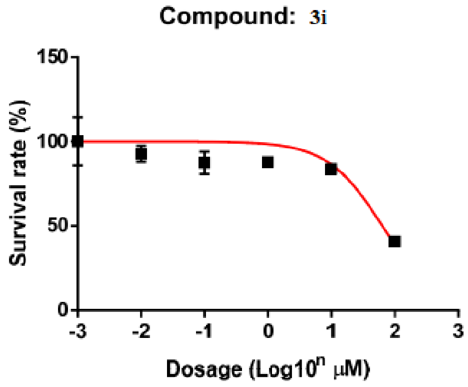


IC$_{50}$ (μM) = 63.69
Log IC$_{50}$ (μM) = 1.80

- Cell line: MCF7 (2000 cells/per well[96])
- Treated time: 72hrs
- Assay: MTT (90mins incubated)
- Data: 3i

| Conc. (μM) | Viability | |
|---|---|---|
| | AVE. | ± SD. |
| **0** | 100.00 | 10.10 |
| **0.01** | 92.65 | 4.79 |
| **0.1** | 87.45 | 6.60 |
| **1** | 87.89 | 1.99 |
| **10** | 83.77 | 1.75 |
| **100** | 40.98 | 0.58 |

MCF-7 Log curve of **3i**

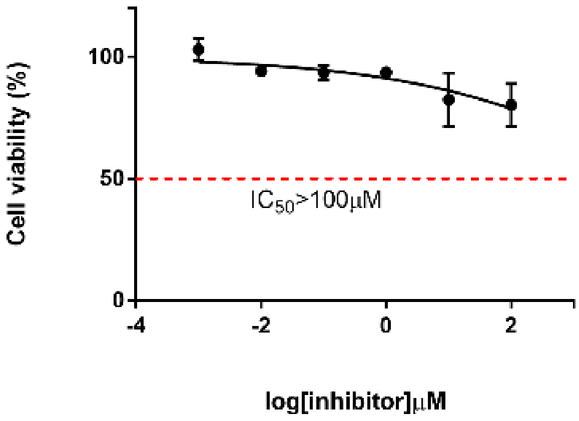


T47D Log curve of 3i

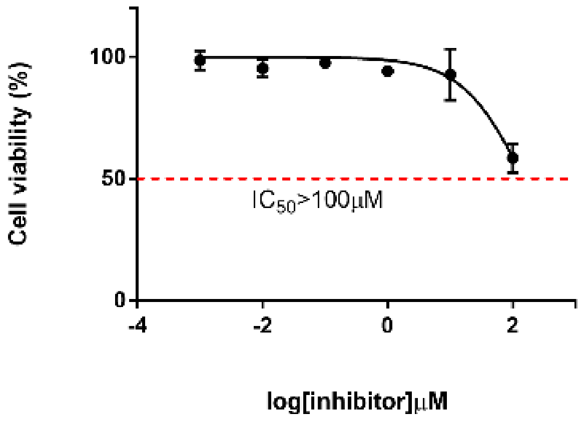


BT474 Log curve of 3i

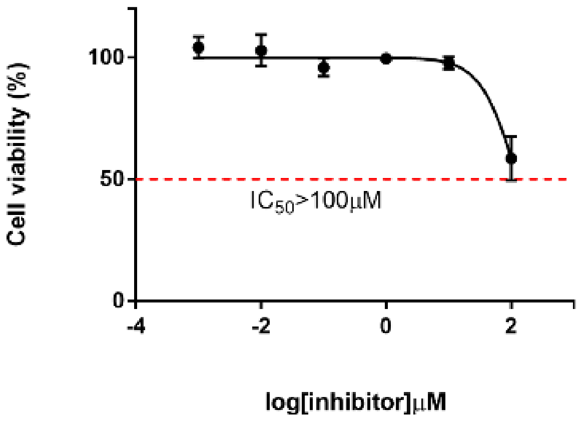


SK-BR-3 log curve of 3i

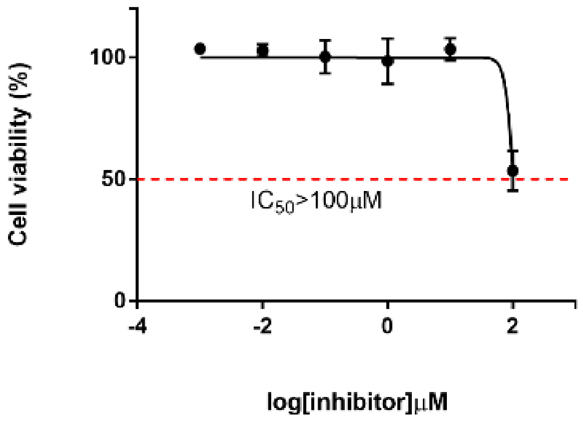


MCF-10A log curve of 3i

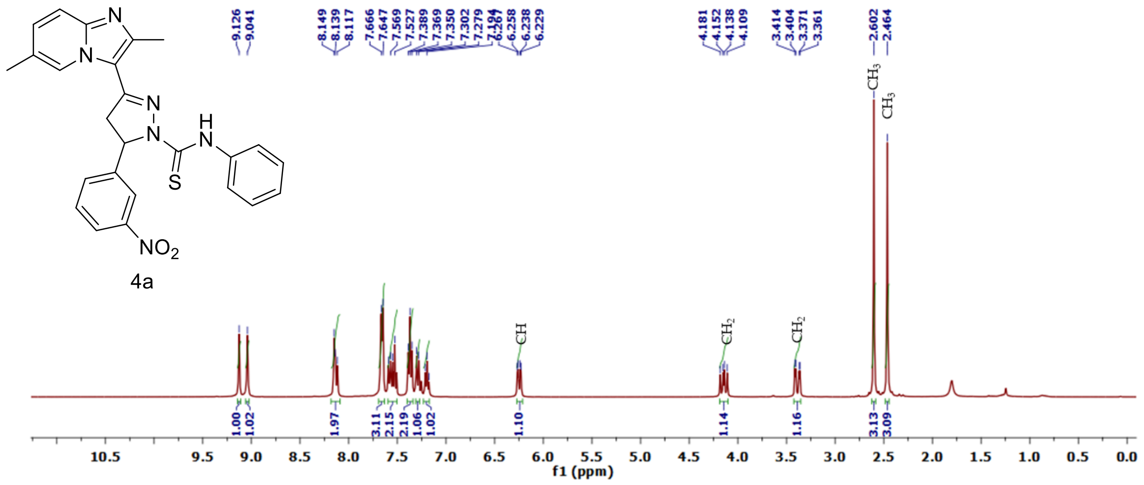


$^{1}$H NMR of **4a**

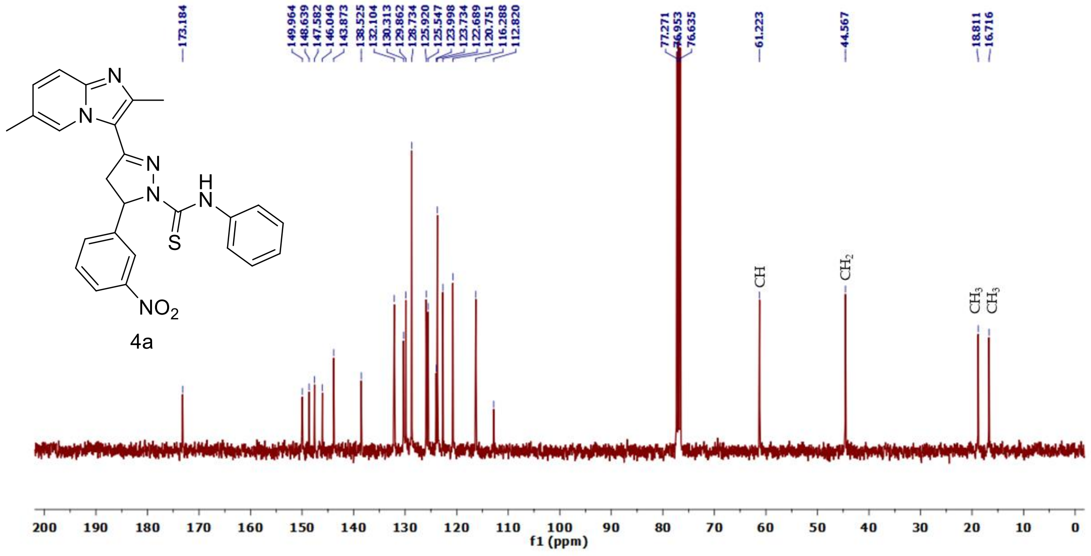


$^{13}$C NMR of **4a**

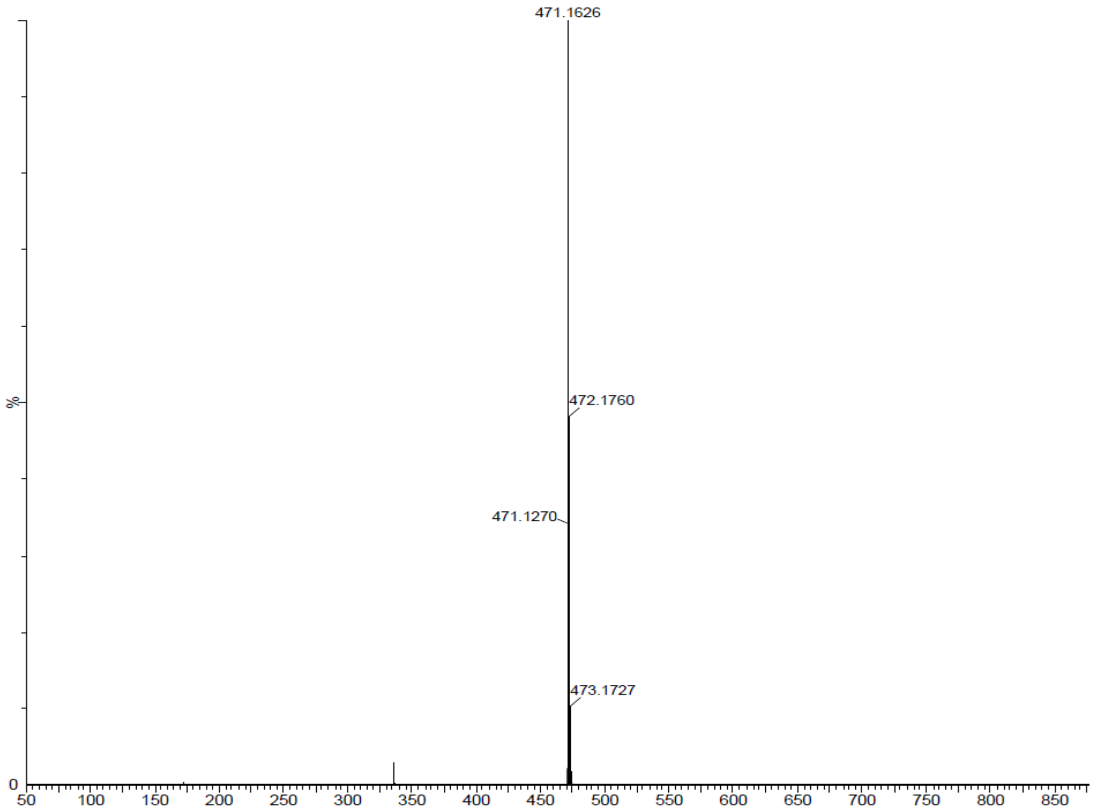


Mass spectra of **4a**

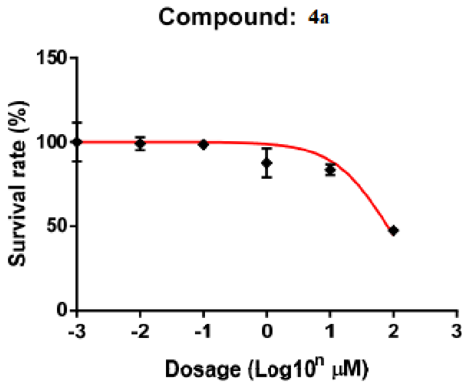


$IC_{50}$ (μM) = 81.37
Log $IC_{50}$ (μM) = 1.91

- **Cell line: MCF7 (2000 cells/per well[96])**
- **Treated time: 72hrs**
- **Assay: MTT (90mins incubated)**
- **Data: 4a**

| Conc. (μM) | Viability | |
|---|---|---|
| | AVE. | ± SD. |
| **0** | 100.00 | 8.15 |
| **0.01** | 99.09 | 3.95 |
| **0.1** | 98.50 | 1.23 |
| **1** | 87.69 | 8.66 |
| **10** | 83.51 | 3.17 |
| **100** | 47.59 | 0.37 |

MCF-7 Log curve of **4a**

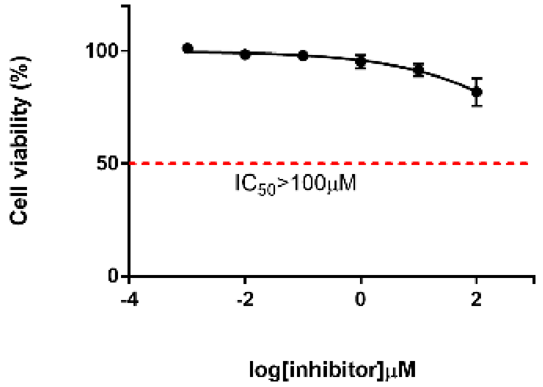


T47D Log curve of 4a

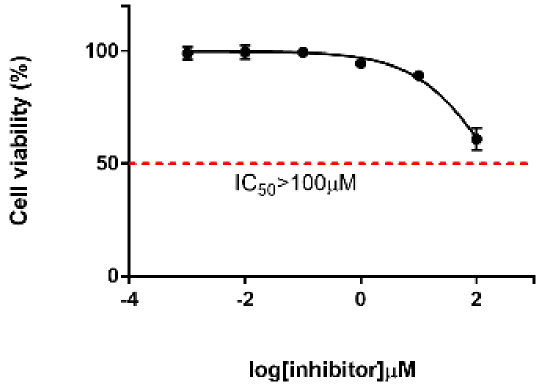


BT474 Log curve of 4a

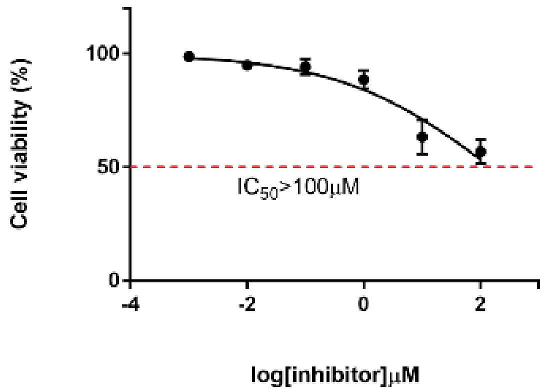


SK-BR-3 log curve of 4a

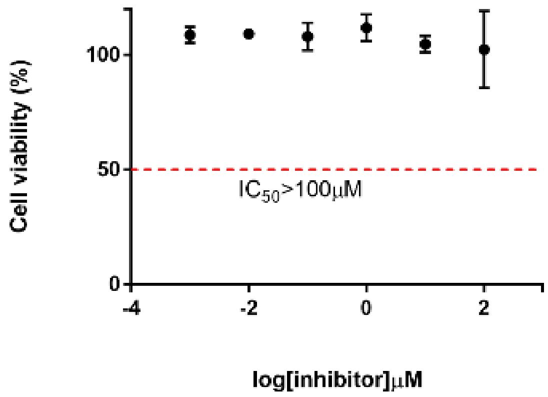


MCF-10A log curve of 4a

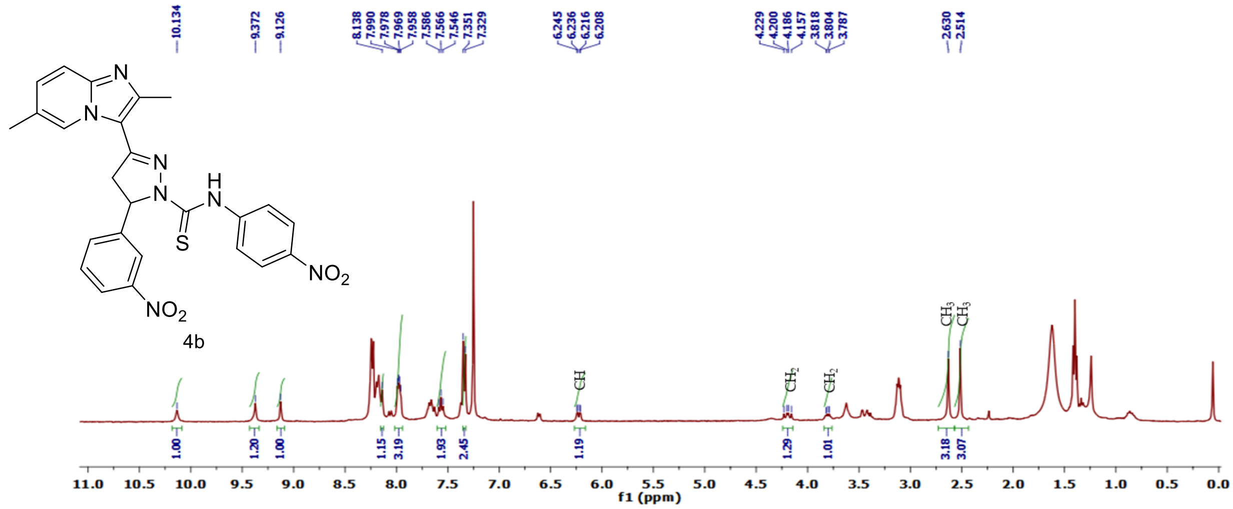


$^{1}$H NMR of **4b**

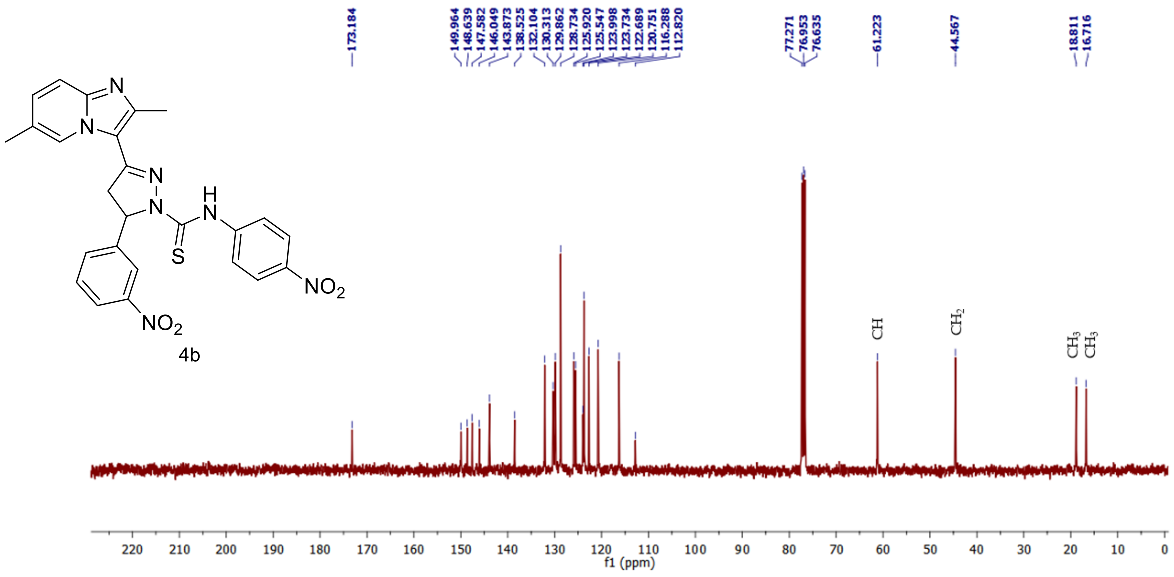


$^{13}$C NMR of **4b**

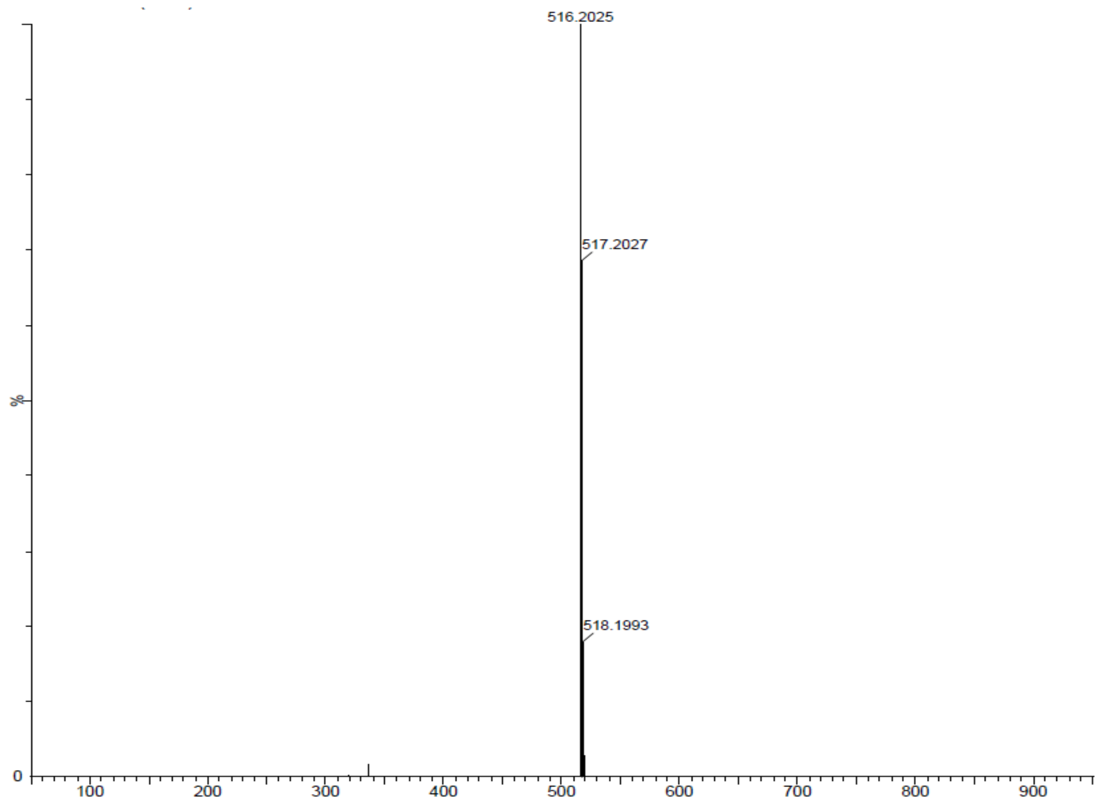


Mass spectra of **4b**

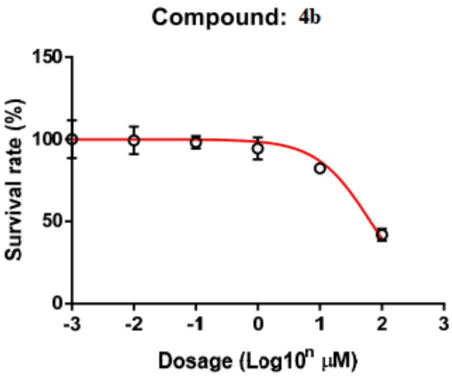


$IC_{50}$ (μM) = 65.36
Log $IC_{50}$ (μM) = 1.82

- Cell line: MCF7 (2000 cells/per well[96])
- Treated time: 72hrs
- Assay: MTT (90mins incubated)
- Data: 4b

| Conc. (μM) | Viability AVE. | Viability ± SD. |
|---|---|---|
| 0 | 100.00 | 8.15 |
| 0.01 | 99.30 | 8.37 |
| 0.1 | 98.13 | 3.79 |
| 1 | 94.47 | 6.67 |
| 10 | 82.35 | 0.89 |
| 100 | 41.89 | 3.66 |

MCF-7 Log curve of **4b**

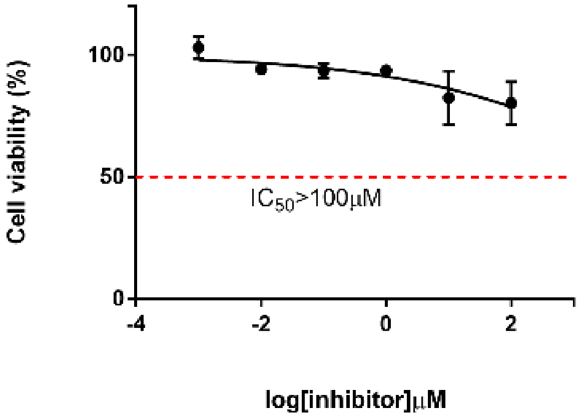


T47D Log curve of 4b

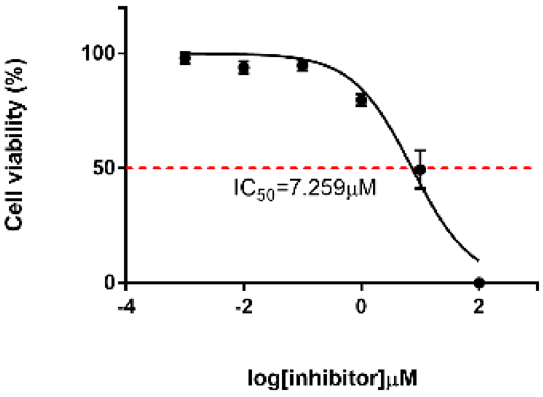


BT474 Log curve of 4b

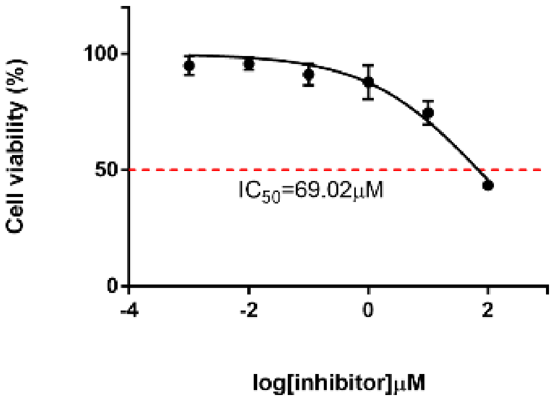


SK-BR-3 log curve of 4b

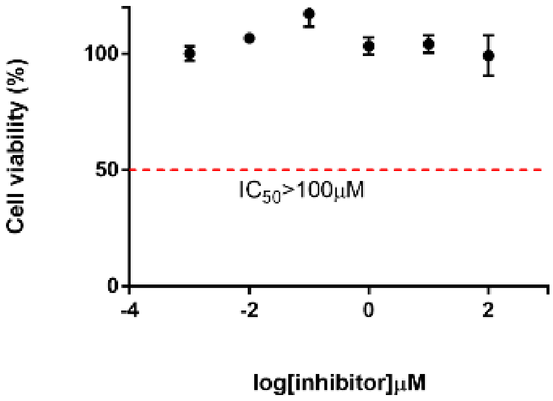


MCF-10A log curve of 4b

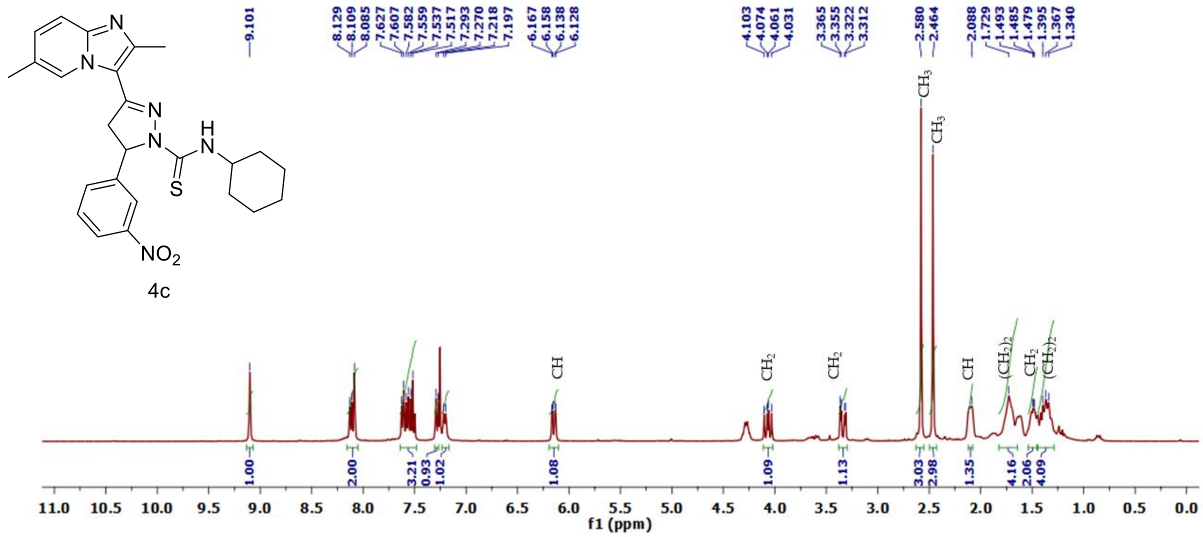

$^{1}$H NMR of 4c

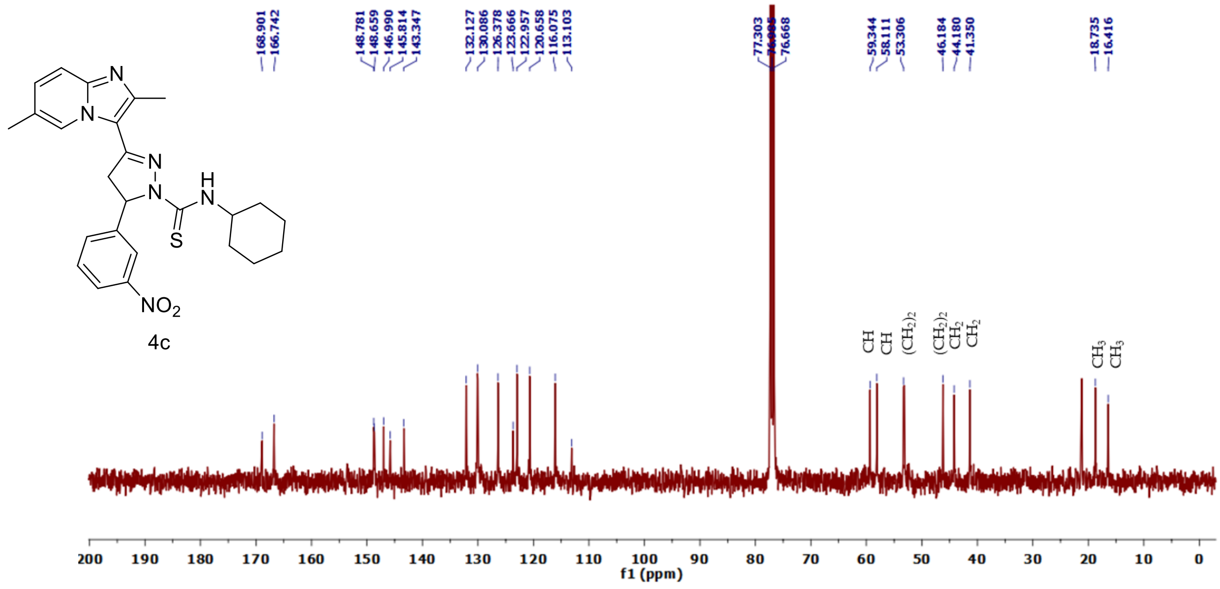

$^{13}$C NMR of 4c

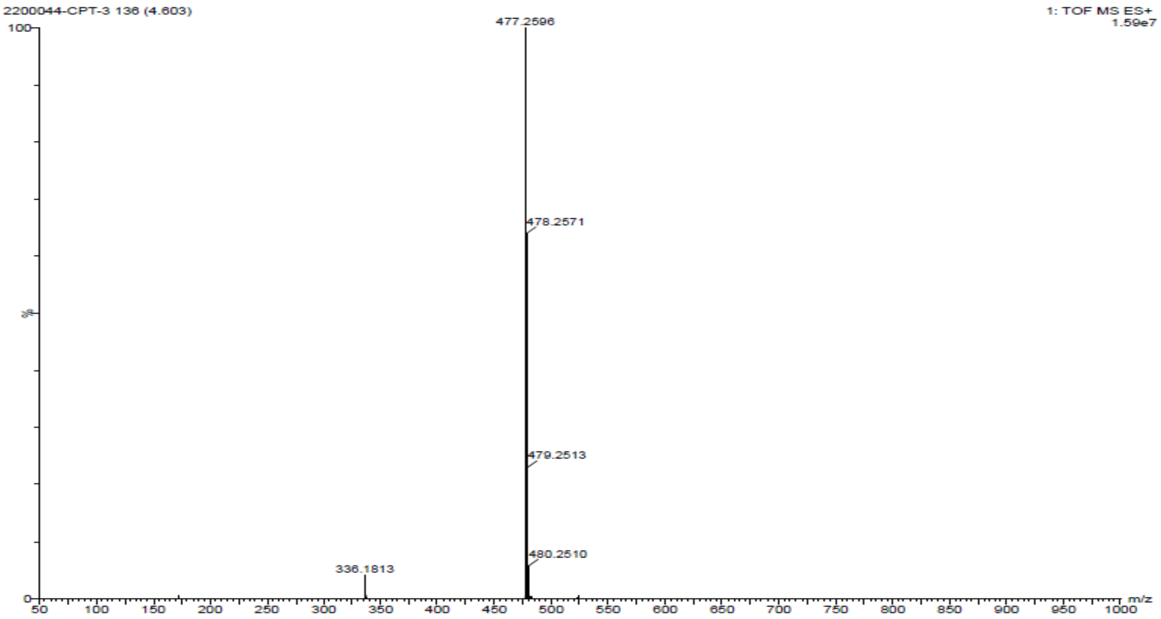


Mass spectra of compound 4c

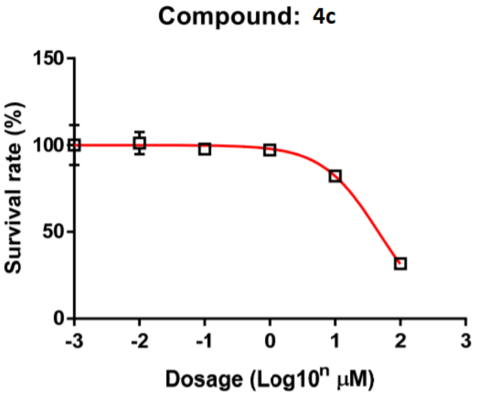


$IC_{50}$ (μM) = 46.08
Log $IC_{50}$ (μM) = 1.66

- **Cell line: MCF7 (2000 cells/per well[96])**
- **Treated time: 72hrs**
- **Assay: MTT (90mins incubated)**
- **Data: 4c**

| **Conc. (μM)** | **Viability** | |
|---|---|---|
| | **AVE.** | **± SD.** |
| **0** | 100.00 | 8.15 |
| **0.01** | 101.22 | 6.44 |
| **0.1** | 97.69 | 1.91 |
| **1** | 97.19 | 0.16 |
| **10** | 82.14 | 1.65 |
| **100** | 31.64 | 0.94 |

MCF-7 Log curve of **4c**

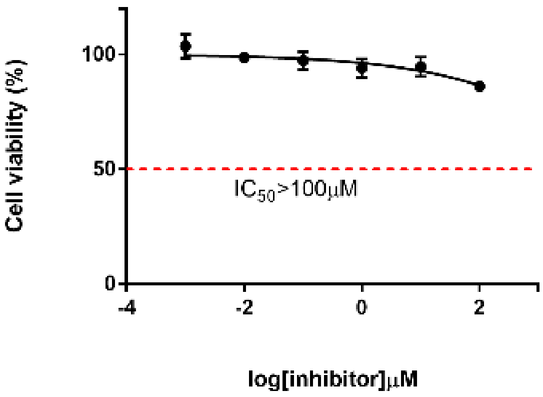


T47D Log curve of 4c

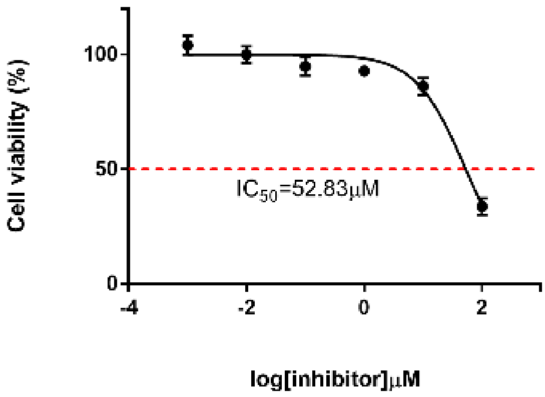


BT474 Log curve of 4c

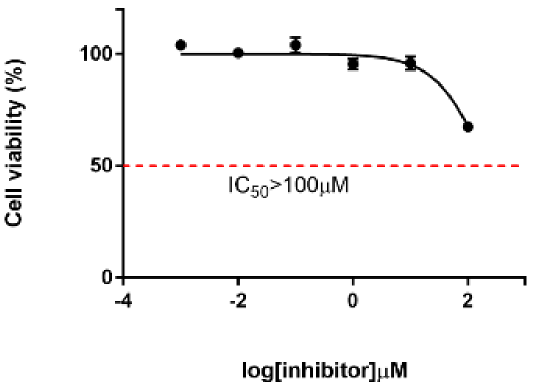


SK-BR-3 log curve of 4c

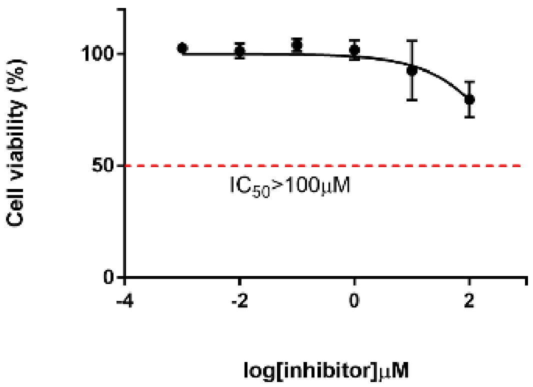


MCF-10A log curve of 4c

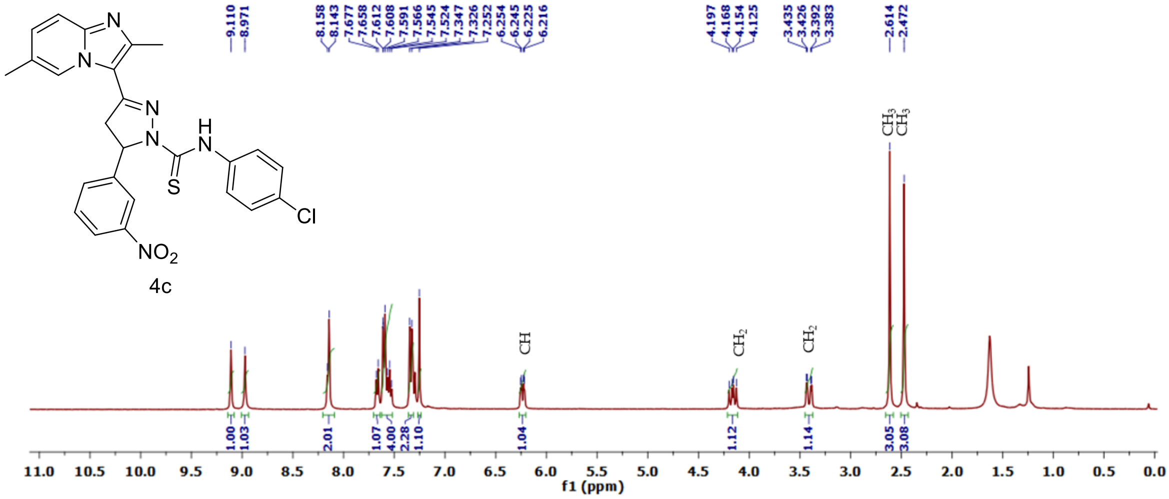

$^1H$ NMR of **4d**

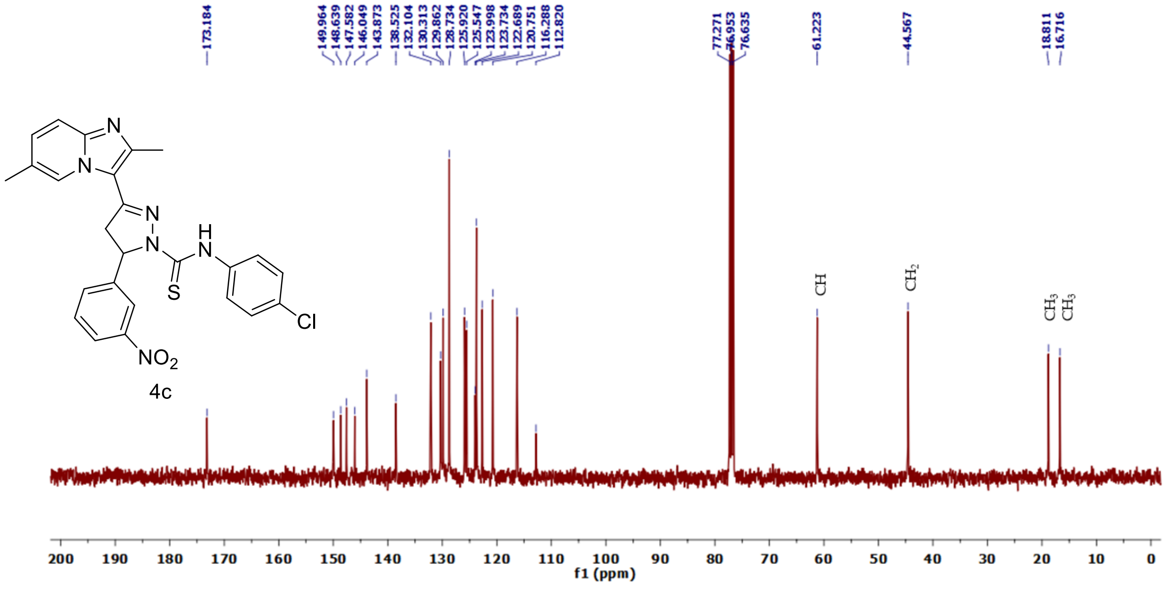

$^{13}C$ NMR of **4d**

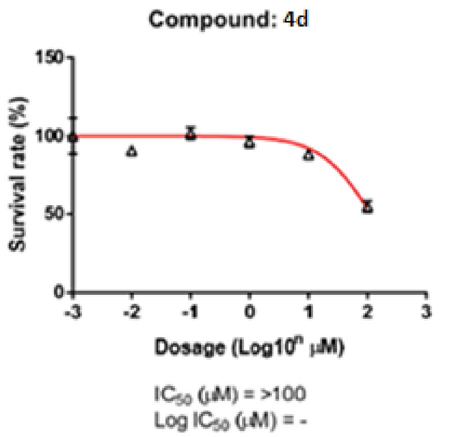


- Cell line: MCF7 (2000 cells/per well[96])
- Treated time: 72hrs
- Assay: MTT (90mins incubated)
- Data: 4d

| Conc. (μM) | Viability | |
|---|---|---|
| | AVE. | ± SD. |
| 0 | 100.00 | 8.15 |
| 0.01 | 90.64 | 0.94 |
| 0.1 | 101.68 | 3.79 |
| 1 | 96.32 | 3.43 |
| 10 | 88.34 | 1.94 |
| 100 | 54.96 | 3.56 |

MCF-7 Log curve of **4d**

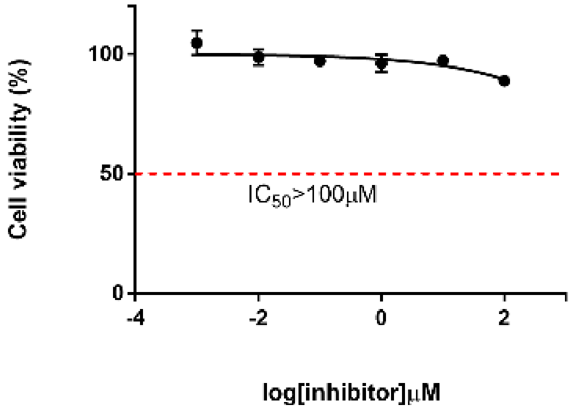


T47D Log curve of 4d

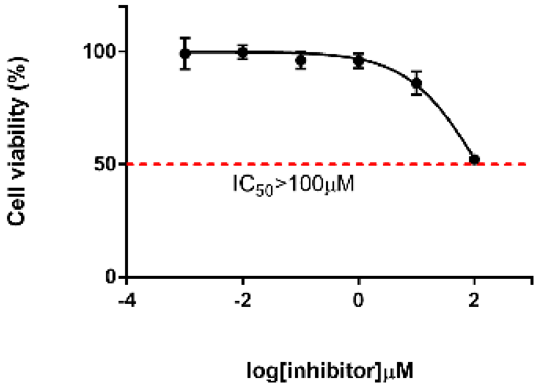


BT474 Log curve of 4d

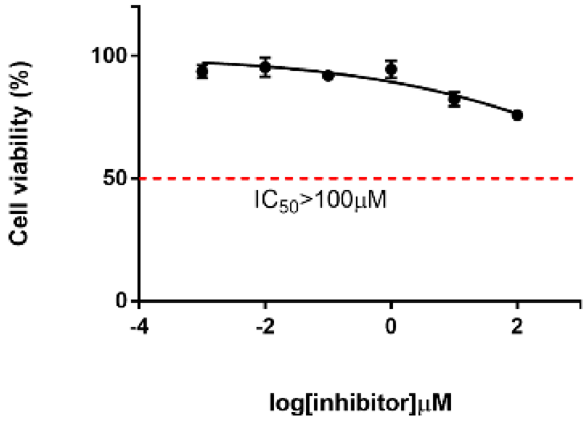


SK-BR-3 log curve of 4d

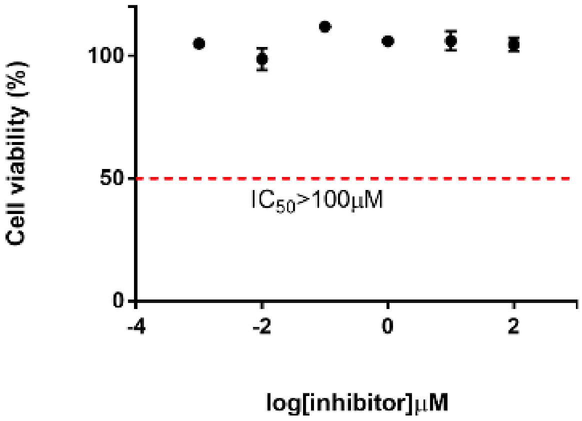


MCF-10A log curve of 4d

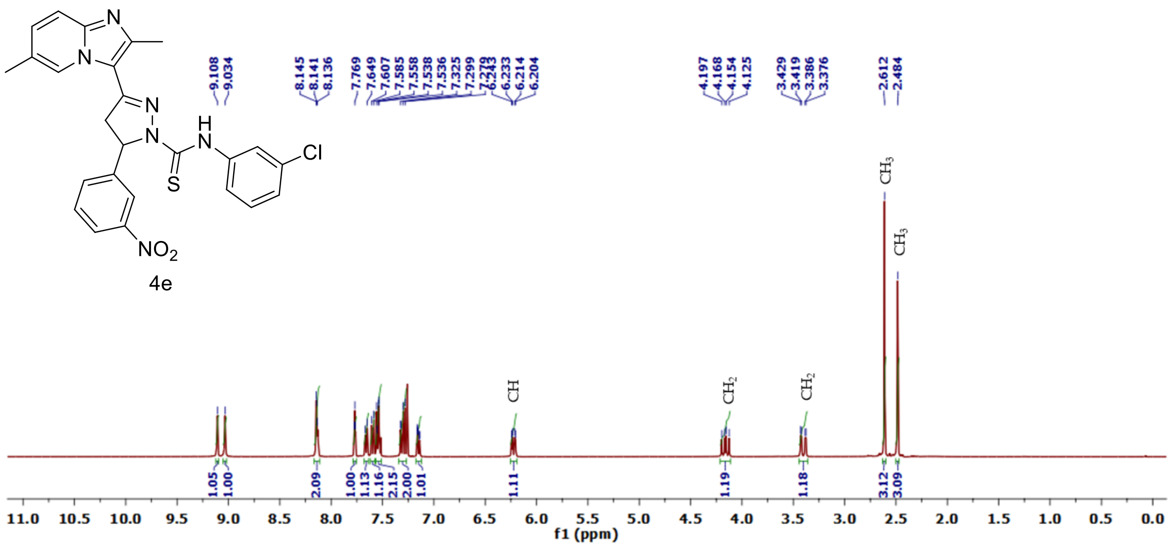

$^{1}$H NMR of 4e

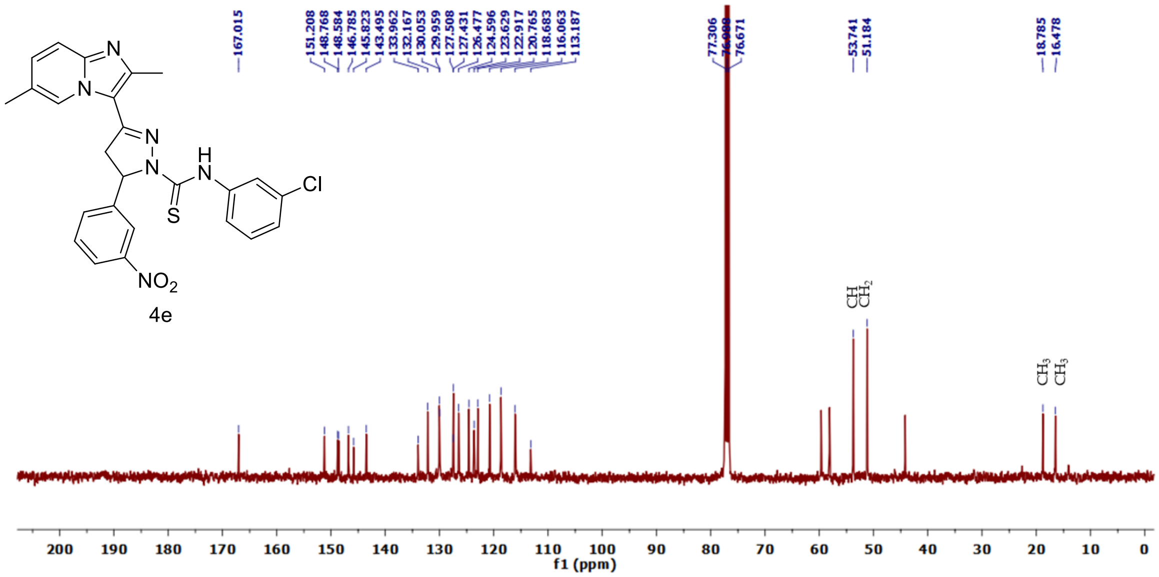

$^{13}$C NMR of 4e

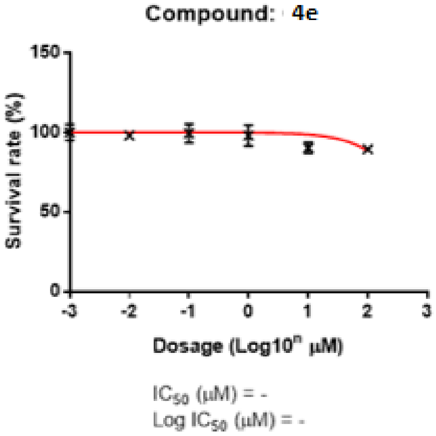


- Cell line: MCF7 (2000 cells/per well[96])
- Treated time: 72hrs
- Assay: alamarBlue (4hrs incubated)
- Data: 4e

| Conc. (μM) | Viability | |
|---|---|---|
| | AVE. | ± SD. |
| 0 | 100.00 | 5.15 |
| 0.01 | 98.04 | 0.80 |
| 0.1 | 99.43 | 5.84 |
| 1 | 98.11 | 6.52 |
| 10 | 90.29 | 3.55 |
| 100 | 89.47 | 2.21 |

MCF-7 Log curve of **4e**

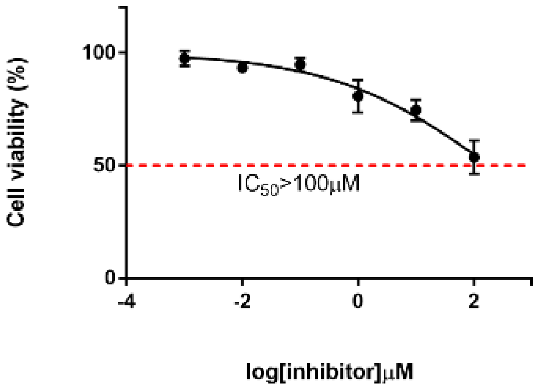


T47D Log curve of 4e

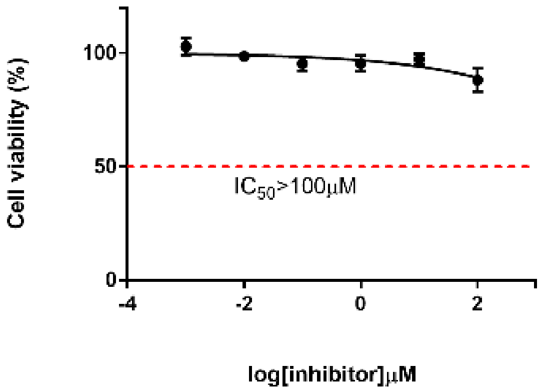


BT474 Log curve of 4e

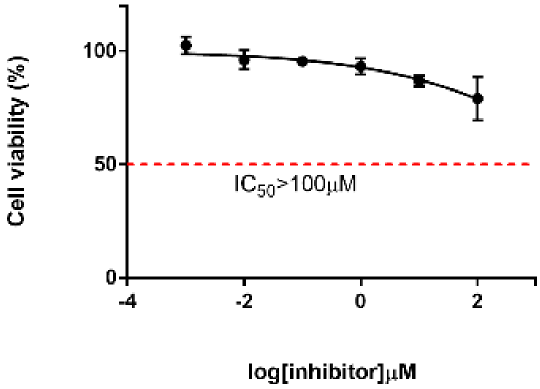


SK-BR-3 log curve of 4e

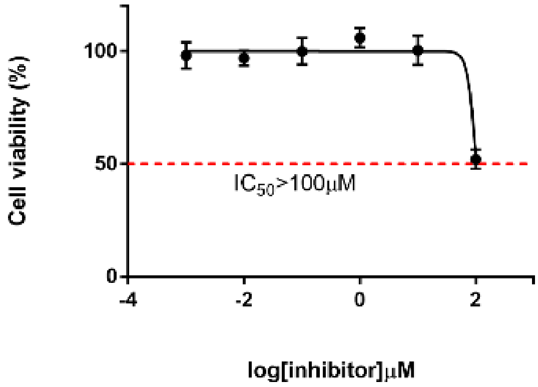


MCF-10A log curve of 4e

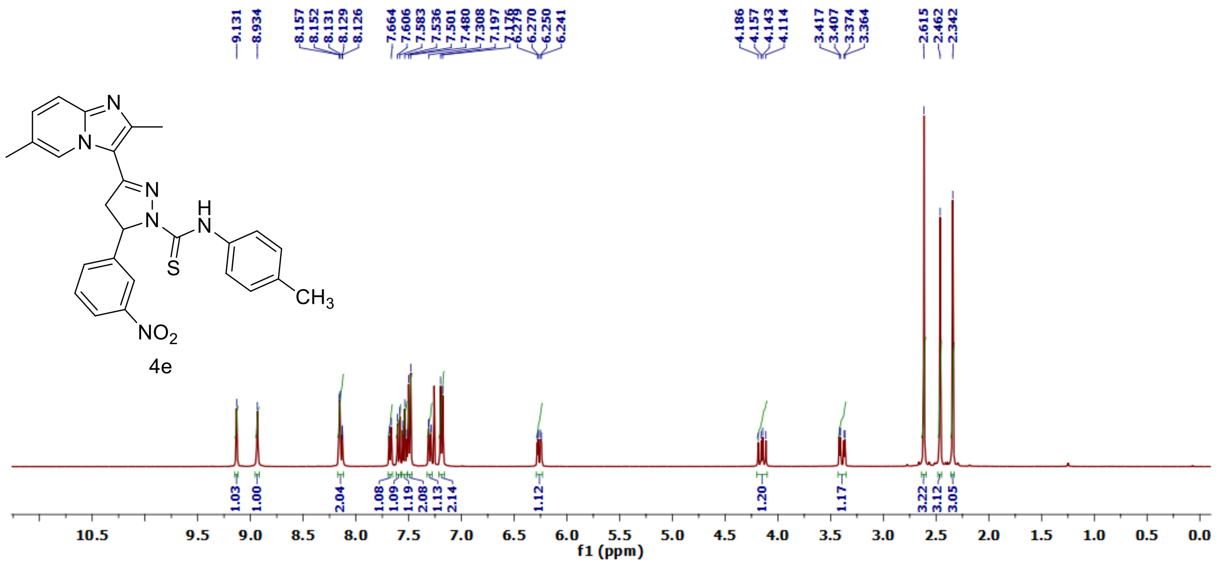

$^{1}H$ NMR of **4f**

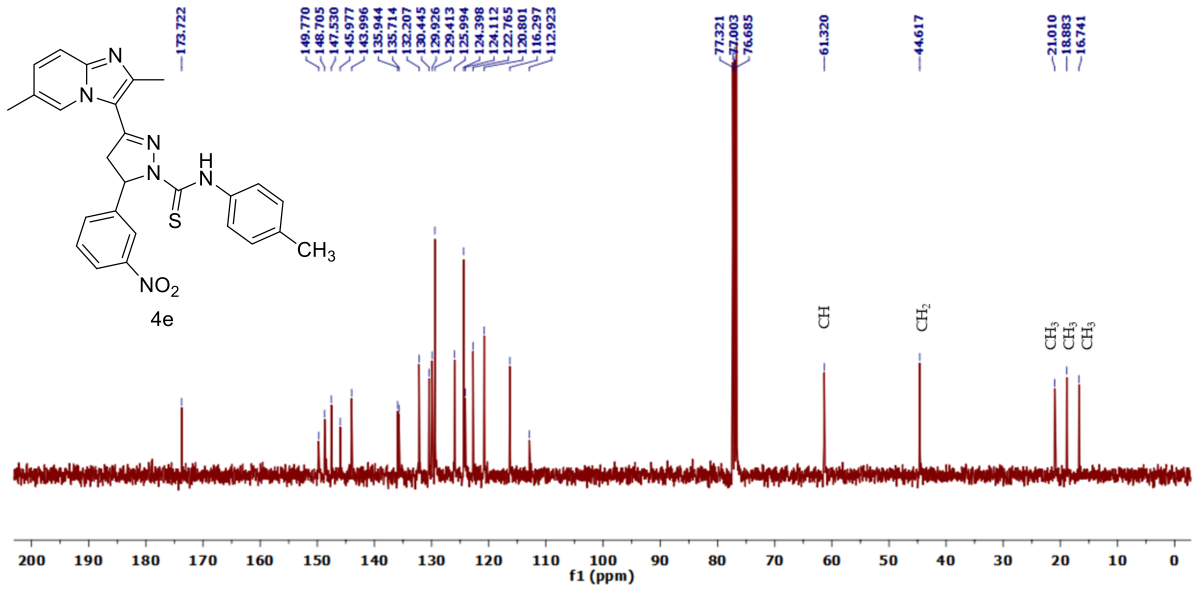

$^{13}C$ NMR of **4f**

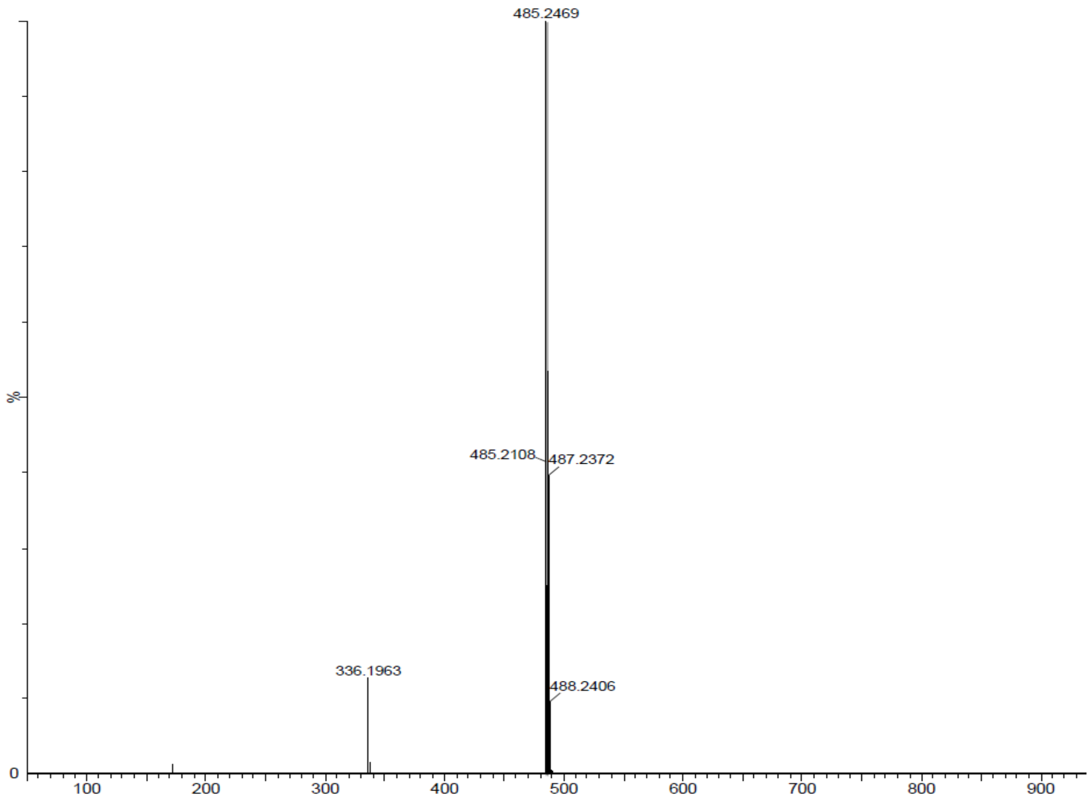


Mass spectra of **4f**

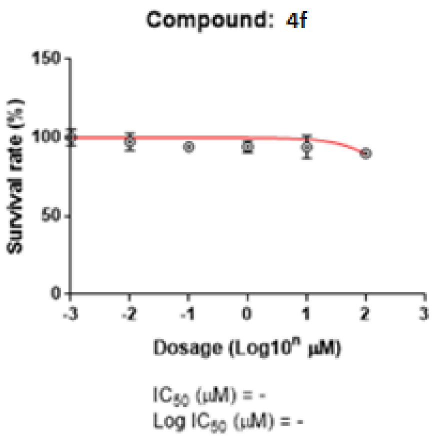


- Cell line: MCF7 (2000 cells/per well[36])
- Treated time: 72hrs
- Assay: alamarBlue (4hrs incubated)
- Data: **4f**

| Conc. (μM) | Viability | |
|---|---|---|
| | AVE. | ± SD. |
| **0** | 100.00 | 5.15 |
| **0.01** | 97.29 | 5.46 |
| **0.1** | 94.01 | 2.88 |
| **1** | 94.16 | 3.55 |
| **10** | 93.92 | 7.15 |
| **100** | 89.99 | 2.97 |

MCF-7 Log curve of **4f**

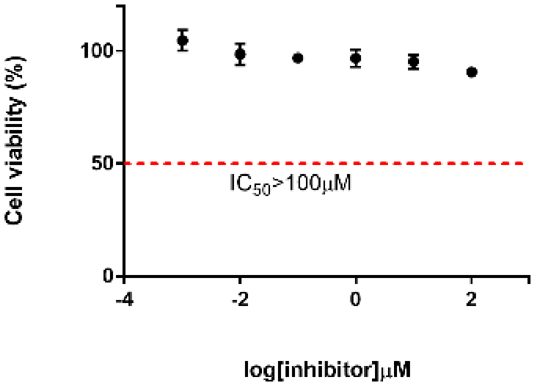


T47D Log curve of 4f

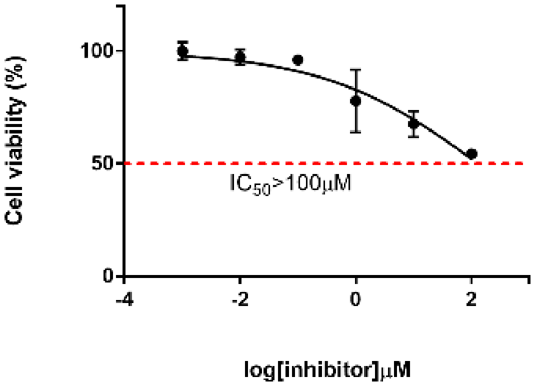


BT474 Log curve of 4f

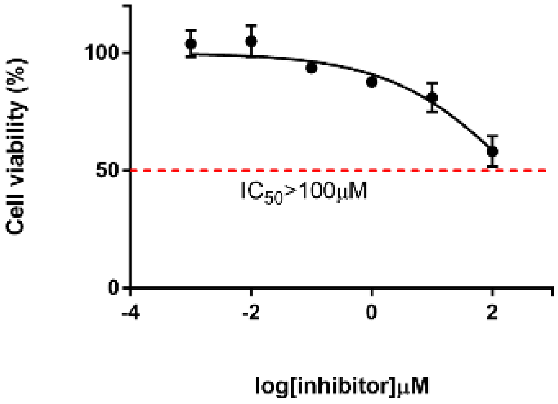


SK-BR-3 log curve of 4f

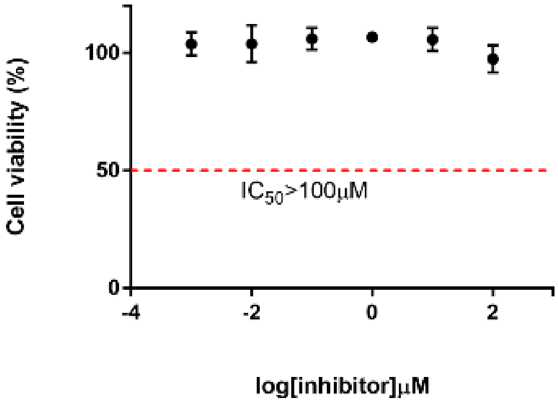


MCF-10A log curve of 4f

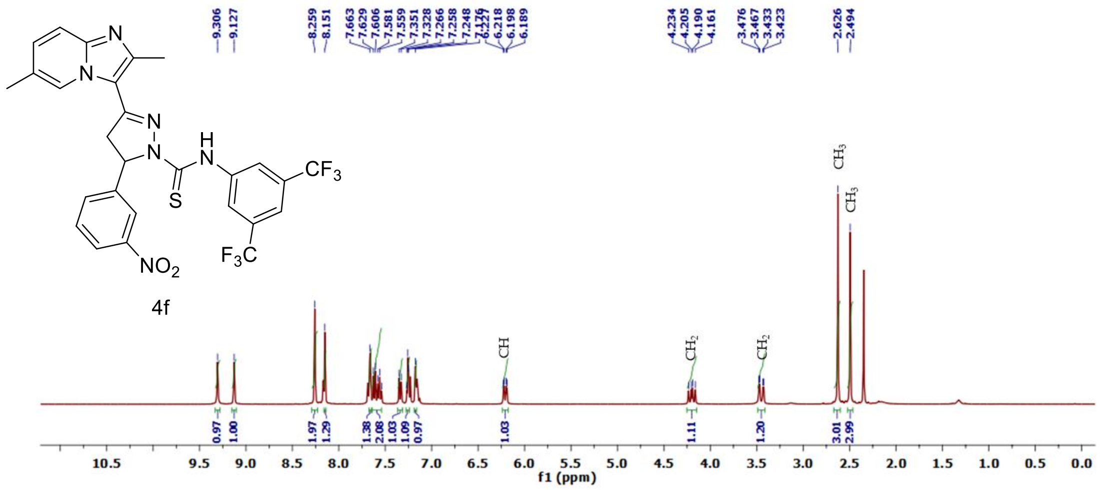


$^{1}H$ NMR of **4g**

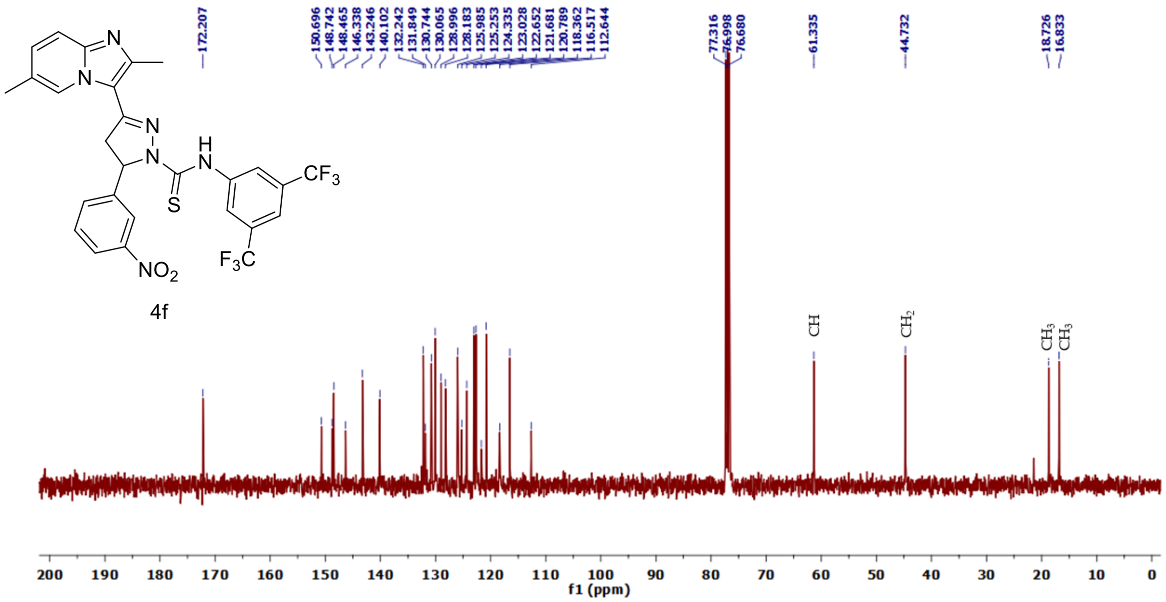


$^{13}$C NMR of **4g**

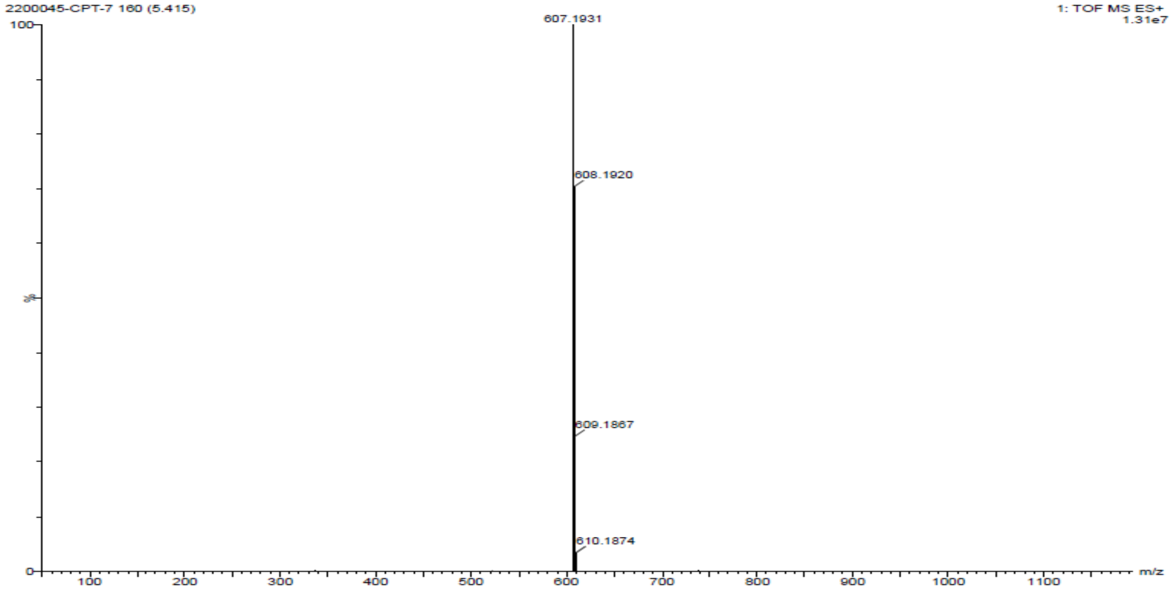


Mass spectra of compound **4g**

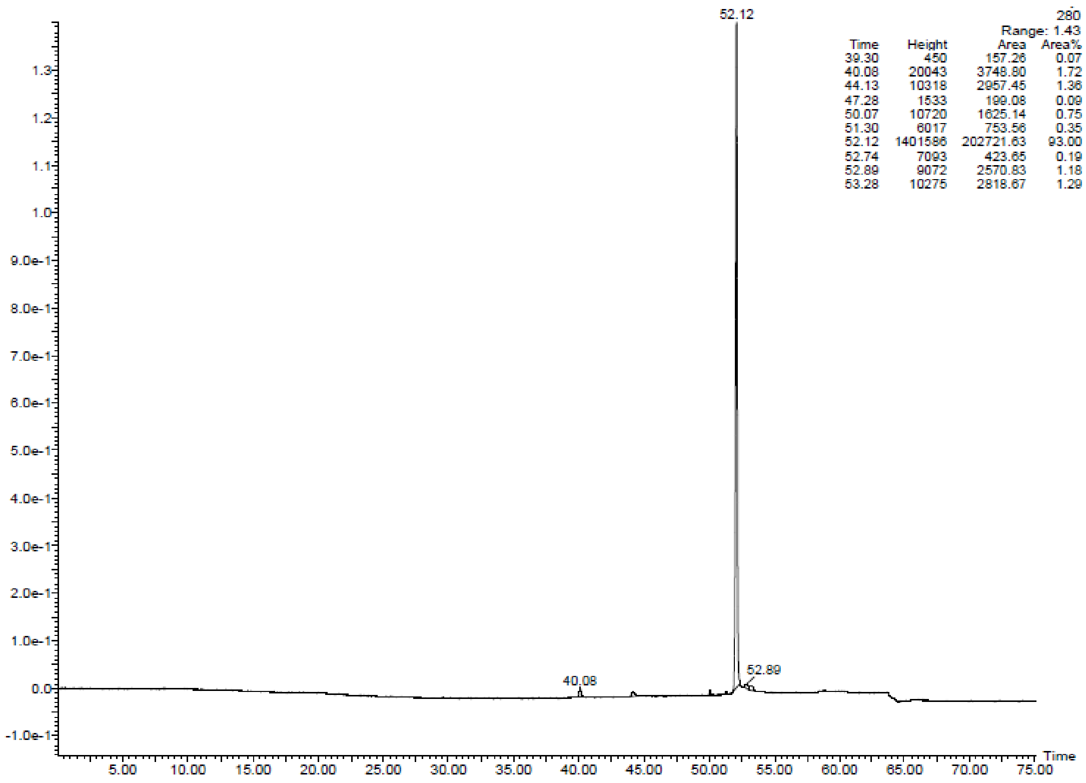


| Time | Height | Area | Area% |
|---|---|---|---|
| 39.30 | 450 | 157.26 | 0.07 |
| 40.08 | 20043 | 3748.80 | 1.72 |
| 44.13 | 10318 | 2957.45 | 1.36 |
| 47.28 | 1533 | 199.08 | 0.09 |
| 50.07 | 10720 | 1625.14 | 0.75 |
| 51.30 | 6017 | 753.56 | 0.35 |
| 52.12 | 1401586 | 202721.63 | 93.00 |
| 52.74 | 7093 | 423.65 | 0.19 |
| 52.89 | 9072 | 2570.83 | 1.18 |
| 53.28 | 10275 | 2818.67 | 1.29 |

HPLC of **4g**

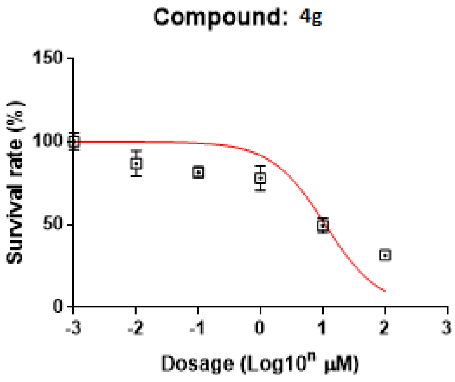


$IC_{50}$ (μM) = 10.90
Log $IC_{50}$ (μM) = 1.04

- Cell line: MCF7 (2000 cells/per well[96])
- Treated time: 72hrs
- Assay: alamarBlue (4hrs incubated)
- Data: 4g

| Conc. (μM) | Viability | |
|---|---|---|
| | AVE. | ± SD. |
| 0 | 100.00 | 5.15 |
| 0.01 | 86.63 | 7.66 |
| 0.1 | 81.43 | 3.26 |
| 1 | 77.91 | 7.47 |
| 10 | 49.40 | 4.47 |
| 100 | 31.40 | 1.95 |

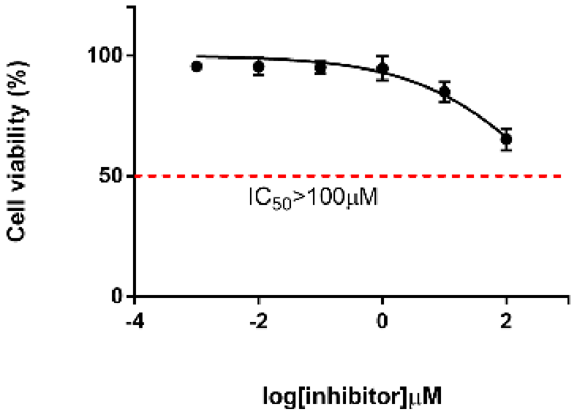


T47D Log curve of 4g

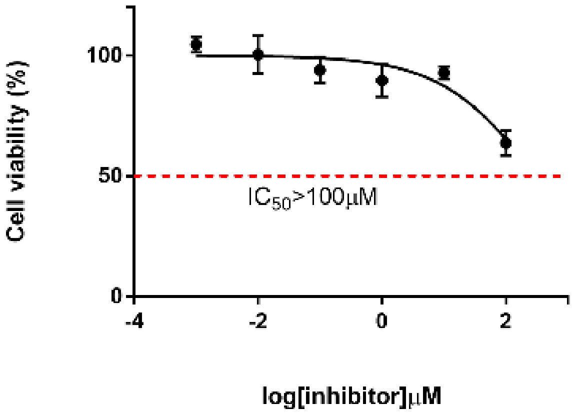


BT474 Log curve of 4g

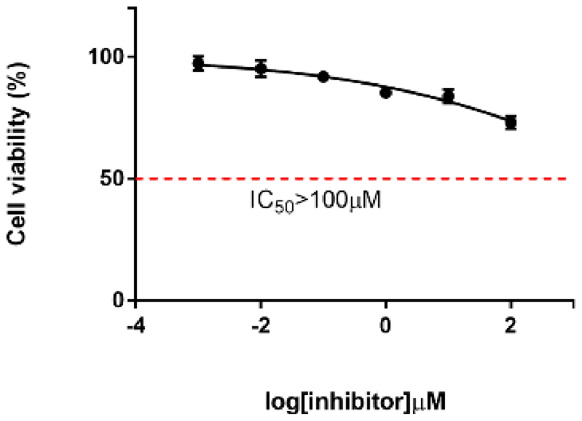


SK-BR-3 log curve of 4g

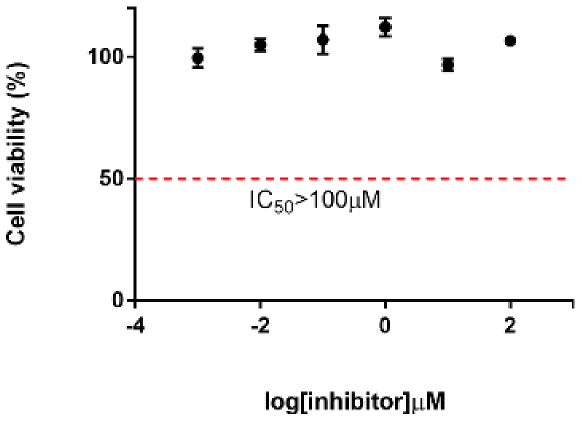


MCF-10A log curve of 4g

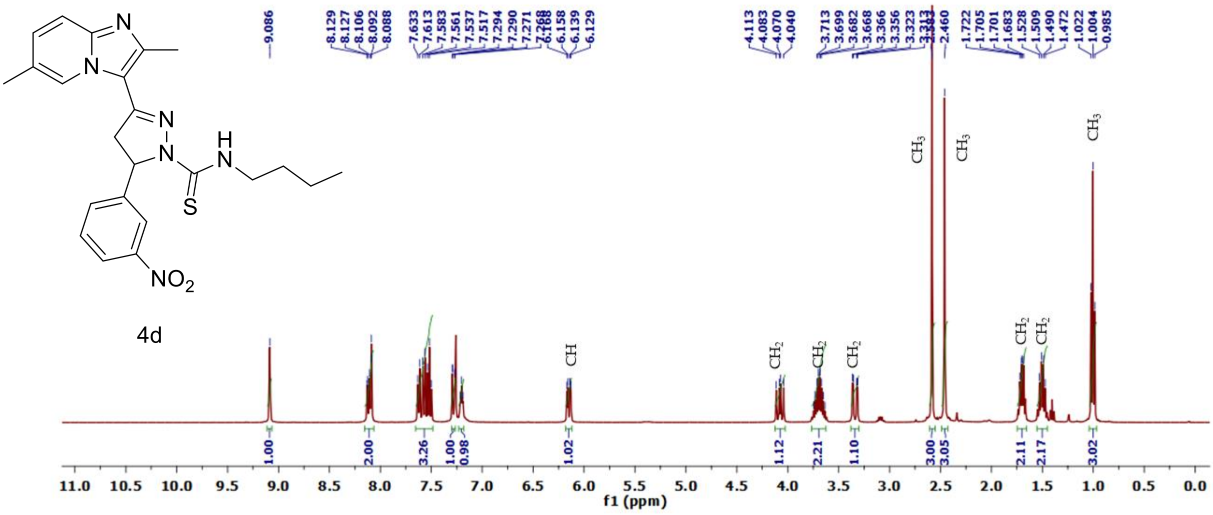


$^{1}$H NMR of **4h**

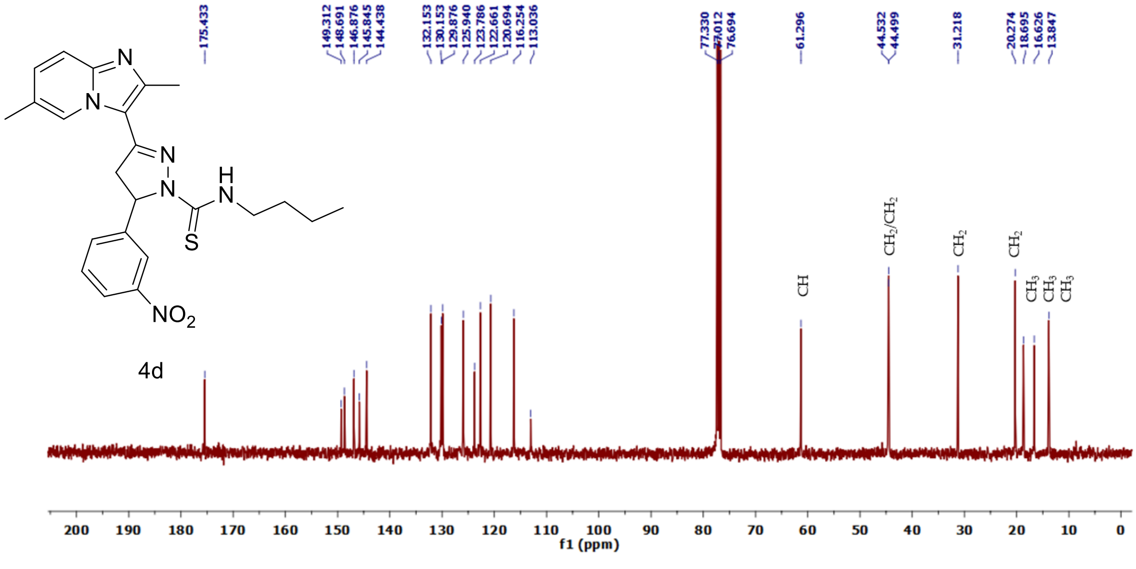


$^{13}$C NMR of **4h**

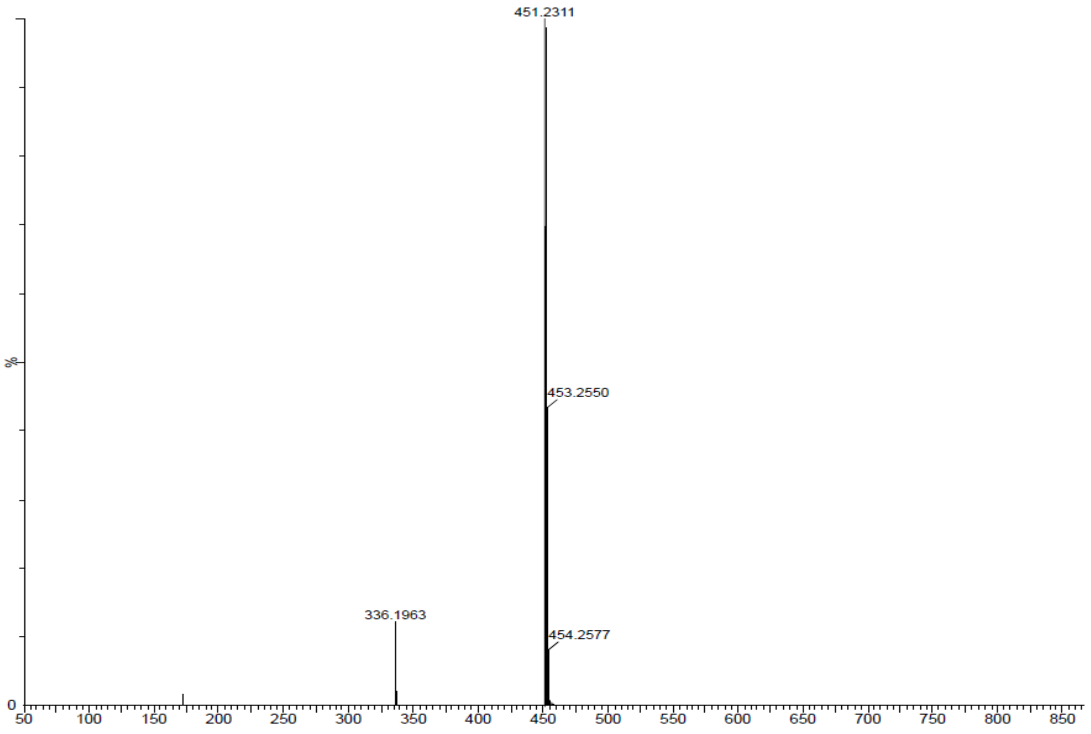


Mass spectra of **4h**

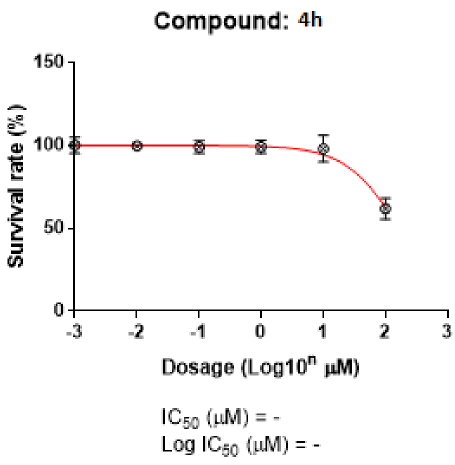


- Cell line: MCF7 (2000 cells/per well[96])
- Treated time: 72hrs
- Assay: alamarBlue (4hrs incubated)
- Data: 4h

| Conc. (μM) | Viability | |
|---|---|---|
| | AVE. | ± SD. |
| 0 | 100.00 | 4.95 |
| 0.01 | 99.75 | 2.11 |
| 0.1 | 99.10 | 3.87 |
| 1 | 99.22 | 4.08 |
| 10 | 97.99 | 8.08 |
| 100 | 61.79 | 6.23 |

MCF-7 Log curve of **4h**

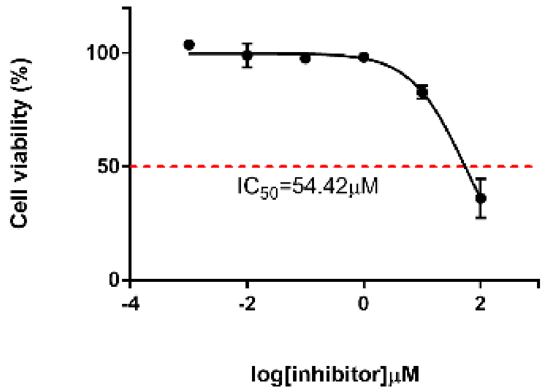


T47D Log curve of 4h

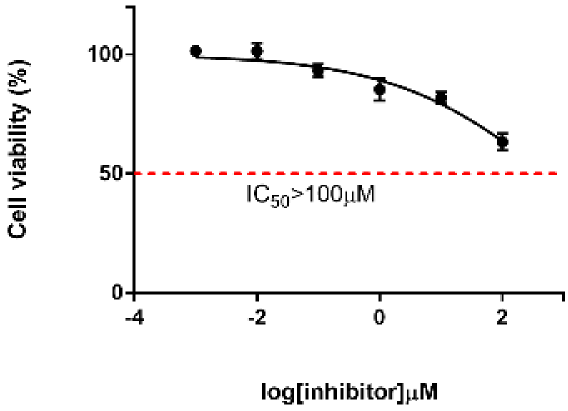


BT474 Log curve of 4h

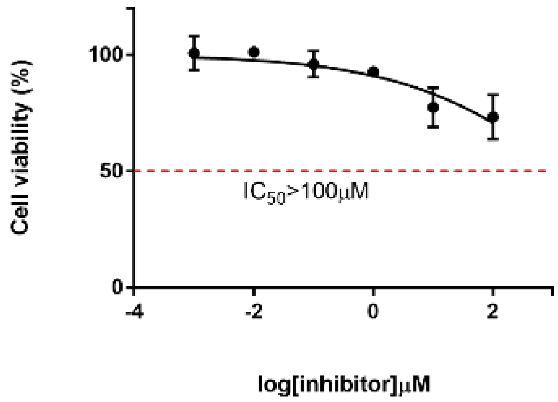


SK-BR-3 log curve of 4h

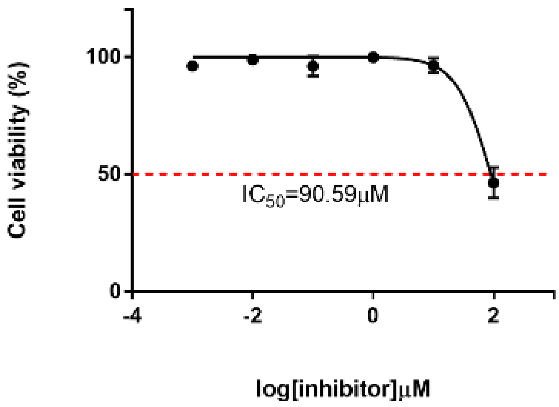


MCF-10A log curve of 4h
